# Surveillance of severe acute respiratory infections associated with SARS-CoV-2, influenza virus and RSV using ICD-10 codes: a case definition accuracy study across five European countries, 2021 to 2023

**DOI:** 10.2807/1560-7917.ES.2025.30.27.2400748

**Published:** 2025-07-10

**Authors:** Miguel Angel Sanchez Ruiz, Diogo FP Marques, Frederikke Kristensen Lomholt, Lasse Skafte Vestergaard, Susana Monge, Marcos Lozano Álvarez, Gudrun Aspelund, Marianna Thordardottir, Ausra Dziugyte, John-Paul Cauchi, Tjarda M Boere, Irene K Veldhuijzen, Elina Seppälä, Håkon Bøås, Trine Hessevik Paulsen, ESURE SARI group, Ausenda Machado, Ana Paula Rodrigues, Mariette Hooiveld, Luis Alves de Sousa, Ana Torres, Carlos Carvalho, Baltazar Nunes, ESURE SARI group, Pello Latasa Zamalloa, Fernando Gonzalez Carril, Liher Imaz Goienetxea, Mikel Ogueta Lana, Eduardo Inchausti Artesero, Aitziber Etxagibel Galdo, Jon Eziolaza Sainz Trapaga, Alba Moya Garcés, Carlota Ruiz de Porras, Luca Basile, Jacobo Mendioroz, María-Teresa Otero-Barrós, Olaia Pérez-Martínez, Ana Sofia Lameiras Azevedo, Miriam López Torrijos, Anthony Nardone

**Affiliations:** 1 Epiconcept, Paris, France; 2 Department of Infectious Disease Epidemiology and Prevention, Statens Serum Institut, Copenhagen, Denmark; 3 Department of Communicable Diseases, National Centre of Epidemiology, Institute of Health Carlos III, Madrid, Spain; 4 Consortium for Biomedical Research on Infectious Diseases (CIBERINFEC), Madrid, Spain; 5 Consortium for Biomedical Research on Epidemiology and Public Health (CIBERESP), Madrid, Spain.; 6 Centre for Health Security and Communicable Disease Control, Directorate of Health, Reykjavik, Iceland; 7 Infectious Disease Prevention and Control Unit (IDCU), Health Promotion and Disease Prevention, Msida, Malta; 8 Faculty of Medicine & Dentistry (Malta Campus), Queen Mary University of London, VCT 2520, Victoria, Gozo, Malta; 9 Centre for Infectious Disease Control, National Institute for Public Health and the Environment (RIVM), Bilthoven, the Netherlands; 10 Department of Infection Control and Vaccines, Norwegian Institute of Public Health, Oslo, Norway; 11 The members of the ESURE SARI group are listed under Collaborators; 12 Epidemiology Department, National Institute of Health Doctor Ricardo Jorge, Lisbon, Portugal; 13 Nivel, Utrecht, the Netherlands; 14 European Centre for Disease Prevention and Control, Stockholm, Sweden

**Keywords:** Surveillance, Electronic Health Records, International Classification of Diseases, SARS-CoV-2, Influenza – Human, Respiratory Syncytial Virus – Human

## Abstract

**BACKGROUND:**

Surveillance of severe acute respiratory infections (SARI) using ICD-10 codes from electronic health records (EHR) lacks consensus on optimal case-defining codes.

**AIM:**

We determined codes that maximise sensitivity (Se) and positive predictive value (PPV) for SARI associated with severe acute respiratory syndrome coronavirus 2 (SARS-CoV-2), influenza virus and respiratory syncytial virus (RSV) in Denmark, Iceland, Malta, Norway and Spain.

**METHODS:**

We included hospitalisations from week 21/2021 to 39/2023, with ICD-10 diagnostic codes for respiratory disease (three-character codes J00–J99) or COVID-19 (U07.1, U07.2, country-specific codes for Denmark). We assessed Se and PPV of individual codes against laboratory results. Based on Se and PPV rank-sum, we selected the top 10 codes and combined them into 10 sets per pathogen. We identified sets that maximised the clinical utility index (CUI = Se × PPV), categorised as excellent (≥ 0.81), good (0.64–0.80), satisfactory (0.49–0.63) and poor (< 0.49).

**RESULTS:**

We assessed 395,163 hospitalisations for SARI-SARS-CoV-2, 313,418 for SARI-influenza and 192,936 for SARI-RSV, all tested. For SARI-SARS-CoV-2, code U07.1 (B34.2A, B97.2A for Denmark) had excellent utility in Denmark, Malta, Norway, Spain (≥ 0.82), and good utility in Iceland (0.79). For SARI-influenza, J09, J10 and J11 performed excellently in Denmark, Norway, Spain (≥ 0.83), satisfactorily in Malta (0.52), and poorly in Iceland (0.43). For SARI-RSV, J12, J20 and J21 achieved highest CUI but had poor utility (0.17–0.34).

**CONCLUSIONS:**

COVID-19- and influenza-specific three-character ICD-10 codes accurately identified SARI associated with SARS-CoV-2 and influenza virus. For SARI-RSV, four-character codes should be explored. We recommend context-specific assessments in countries adopting EHR-based surveillance.

**Key public health message**

**What did you want to address in this study and why?**

European countries are developing surveillance systems based on electronic health records (EHR) originally collected for clinical or administrative use. These records typically include diagnostic codes from the ICD-10 classification. We intended to find out which ICD-10 codes can be used to identify severe acute respiratory infections (SARI) associated with high-impact pathogens: SARS-CoV-2, influenza virus and respiratory syncytial virus (RSV).

**What have we learnt from this study?**

Across the participating countries (Denmark, Iceland, Malta, Norway, Spain), we found that similar diagnostic codes were the most suitable for identifying SARI associated with the three pathogens. The identified codes effectively detected SARI associated with SARS-CoV-2 and influenza virus, but were less successful in identifying RSV, highlighting the need to further explore codes for this pathogen.

**What are the implications of your findings for public health?**

The identified diagnostic codes can be used in the participating countries to monitor trends or understand the burden of SARI associated with specific pathogens, thereby informing public health decisions. We applied a common methodology to identify ICD-10 codes for detecting SARI associated with SARS-CoV-2, influenza virus and RSV. This can be replicated by other countries currently implementing EHR-based SARI surveillance in Europe.

## Introduction

Among pathogens causing severe acute respiratory infections (SARI), severe acute respiratory syndrome coronavirus 2 (SARS-CoV-2), influenza and respiratory syncytial virus (RSV) have the largest impact in terms of morbidity, mortality, and absenteeism from work or education [1].

The surveillance of SARI enables the simultaneous monitoring of several respiratory pathogens using case definitions that traditionally rely on symptoms. The World Health Organization (WHO) defines a SARI as an acute respiratory infection with a history of fever or measured temperature of ≥ 38 °C, and cough, with onset within the last 10 days, that requires hospitalisation [2].

Since 2020, most countries in the European Region have established SARI surveillance systems which exhibit considerable heterogeneity [3-14]. Some countries rely on electronic health records (EHR) [9-13] collected and stored for clinical or reimbursement purposes, which may include diagnosis codes such as the 10^th^ edition of the International Classification of Disease codes (ICD-10) [15]. When symptom data are unavailable or not collected in a standardised manner, ICD-10 diagnosis codes may serve as a proxy for identifying SARI associated with specific pathogens [9-13]. However, more studies are required to determine the most suitable ICD-10 codes that define SARI for surveillance purposes and to assess whether common definitions can be used across countries. The absence of this information hampers the implementation of EHR-based SARI surveillance, which complicates the estimation, comparison and pooling of national incidence rates across Europe.

We conducted a study to determine ICD-10 code-based case definitions that maximise the sensitivity (Se) and positive predictive value (PPV) in identifying SARI associated with one specific pathogen (SARS-CoV-2, influenza virus or RSV) and SARI associated with any of the three. This was assessed overall, by age group, and considering national-level SARI activity.

## Methods

### Study design

The study was a retrospective assessment of the accuracy of ICD-10 code-based case definitions to identify SARI associated with SARS-CoV-2, influenza virus or RSV. The reference standard was a pathogen-specific laboratory test result, primarily determined by PCR, with rapid antigen testing (RAT) used alone in 10% or fewer of the samples.

### Study population and study setting

Data were extracted from SARI surveillance systems in five countries and analysed using a common protocol. Data items included the week of hospital admission, ICD-10 diagnosis codes, age group, and pathogen-specific laboratory testing. We did not disaggregate by sex as this is not expected to enhance case identification. Denmark, Iceland, Malta and Norway used nationwide EHR-based surveillance databases with nearly complete coverage of their populations. In contrast, Spain used data from sentinel hospitals in four regions (the Basque Country, Catalonia, the Valencian Community and Galicia) representing 8.3% of the national population.

### Inclusion criteria

We selected records through a two-step process. Firstly, countries identified hospitalisation episodes from patients of all ages who had been routinely assigned at least one three-character ICD-10 diagnosis code for respiratory disease (J00 to J99) or a four-character COVID-19 ICD-10 code (U07.1, U07.2, Denmark used country-specific codes listed in the Supplement). Countries used principal and secondary diagnosis codes assigned mostly at discharge. In Iceland, while the database included both admission and discharge codes, the available information did not allow determining whether a code corresponded to admission or discharge diagnoses. Countries used ICD-10 versions in their national languages. Secondly, we retained those of the identified hospitalisation episodes in which patients had been tested for SARS-CoV-2, influenza viruses (A or B) and/or RSV. The use of PCR (primarily) or RAT, and the time window for laboratory results to be linked to a hospitalisation episode, varied according to country-specific surveillance standards. The details of the inclusion process and definitions per country are described in Table 1. Supplementary Table S1 outlines the testing practices.

**Table 1:** Study population according to SARI surveillance systems, Denmark, Iceland, Malta, Norway, Spain, week 21/2021–39/2023

Country	Patients included	Definitions		ICD-10 codes		Laboratory considerations	
		Hospital admission	Readmission	Accessible	Code class^a^	Pathogens included and type of test	Time window for laboratory confirmation
Denmark	All hospitalisation episodes meeting inclusion criteria	Hospitalised for at least 12 h	Subsequent hospital stays within 14 days from discharge	J00–J99 + country-specific SARS-CoV-2 codes**^b^**	Discharge	SARS-CoV-2, influenza virus, RSV (PCR)	From 10 days before admission to 3 days after admission
Iceland	All hospitalisation episodes meeting inclusion criteria	Admission does not depend on length, can be hours or a day	Subsequent hospital stays within 2 days from discharge	J00–J99 + U07.1, U07.2	Admission and discharge	SARS-CoV-2, influenza (PCR)	From 10 days before admission to 2 days after admission
Malta	All hospitalisation episodes meeting inclusion criteria	Hospitalised for more than 24 h	No use of readmissions, any new contact with a hospital is considered a new admission	J00–J99 + U07.1, U07.2	Discharge	SARS-CoV-2 (RAT and PCR); influenza virus and RSV (PCR)	From 14 days before admission to 3 days after (3 days is a proxy to 48 h within hospital)
Norway	All hospitalisation episodes meeting inclusion criteria	Hospital admissions are identified by a specific variable that categorises the patient contacts as polyclinical, day treatment, or admission	Subsequent hospital stays with no more than 1 day in between	J00–J22, J80 + U07.1, U07.2, J30–J99 excluding J80 are grouped and referred to as J-non-SARI	Discharge	SARS-CoV-2 (RAT and PCR), influenza virus and RSV (PCR)	From 14 days before admission to 2 days after discharge
Spain	Hospitalisation episodes selected as per the national SARI sentinel surveillance protocol and meeting the study inclusion criteria**^c^**	Hospitalised for more than 24 h, exclusions**^d^**	Subsequent hospital stays within 14 days from discharge	J00–J99 + U07	Discharge	SARS-CoV-2, influenza virus and RSV (RAT and PCR)	Sample collection is recommended as soon as possible after admission, and preferably within 7 days of symptom onset

ICD-10: 10th edition of the International Classification of Disease codes; RAT: rapid antigen test; RSV: respiratory syncytial virus; SARI: severe acute respiratory infection; SARS-CoV-2: severe acute respiratory syndrome coronavirus 2.

^a^ Both principal and secondary ICD-10 codes were included in all countries. In Denmark, the surveillance system does not classify codes as either admission or discharge, instead, codes are updated as information becomes available. Since this was a retrospective study, the extracted codes are expected to correspond to discharge ones mostly. In Norway, diagnostic codes are typically registered at discharge, however, in some cases, codes may be recorded as soon as the diagnosis is confirmed, potentially before discharge. Malta and Spain used discharge codes. Iceland used a mix of admission and discharge codes, but these could not be distinguished from one another.

^b^ Denmark used country-specific codes for COVID-19, as follows: B97.2A2A: COVID-19 severe acute respiratory syndrome B34.2A2A: COVID-19, not elsewhere classified, Z038PA1: observation due to suspicion of COVID-19 infection.

^c^ As per the Spanish sentinel SARI surveillance protocol, all participating regions but the Basque Country pre-selected admissions compatible with SARI (according to locally defined clinical criteria or at the clinician’s discretion) from specific weekdays (1 day or 2 days a week) and subsequently applied the study inclusion criteria (i.e. hospitalisation episodes having a respiratory disease or COVID-19-specific ICD-10 code were retained for the study). In the Basque Country, the study inclusion criteria were directly applied to all patients hospitalised.

^d^ Exclusions applied in Spain for the definition of hospital admissions: patients under observation in the emergency room, day hospital, haemodialysis sessions, outpatient surgery, or transfers between the hospital's services are not considered hospital admissions.

### Study period

We included hospitalisation episodes between week 21/2021 and week 39/2023. In Malta, patients younger than 18 years were only included in the SARI surveillance system from week 6/2023. National surveillance teams extracted data in or after December 2023.

### Data analysis

#### Descriptive analysis

We described the study population and testing patterns according to age group and national-level SARI activity (i.e. high/low weekly SARI notification rates; for a full list of practices by country see Supplementary Table S2). We described testing patterns as the proportion of hospitalisation episodes in which patients were tested for SARS-CoV-2, influenza virus and/or RSV, along with the proportion of pathogen-specific positive tests.

#### Accuracy assessment

The reference standard for SARI associated with a specific pathogen included hospitalisation episodes from patients with respiratory disease or COVID-19 diagnosis codes and laboratory results for that pathogen (primarily PCR, but also RATs). Laboratory confirmation defined true positive pathogen-specific SARI. For SARI associated with any considered pathogens, true positives were defined by laboratory confirmation of SARS-CoV-2, influenza or RSV in patients tested for all three pathogens during the same hospitalisation. All available codes per patient, whether assigned at discharge or admission (the latter available only in Iceland), corresponding to principal or secondary diagnosis, were included in the analysis. This approach aimed to enhance case definition sensitivity and ensure the identification of patients who may not have been assigned a respiratory disease or COVID-19 code as their principal diagnosis.

We assessed the accuracy of the various code sets or case definitions following a three-step process:

#####  -1- Building case definitions based on single-code accuracy

We calculated the Se and PPV of each ICD-10 code (J00-J99, U07.1, U07.2, Danish COVID-19 codes) for identifying a specific pathogen among SARS-CoV-2, influenza virus and RSV, or any of the three. The Se of an ICD-10 code ***a*** for identifying hospitalisations with laboratory confirmation of pathogen ***p*** (p could equal SARS-CoV-2, influenza virus, RSV or any of the three), was given by


$${Se}_{ap}=\frac{n_{ap}}{n_{.p}}$$

Here, *n_ap_* represents the number of true positive cases (TP), i.e. hospitalisation episodes from patients with the relevant ICD-10 code ***a*** and laboratory confirmation of pathogen ***p****,* and *n_.p_* denotes the total number of hospitalisations with laboratory confirmation of pathogen ***p***. The PPV of an ICD-10 code ***a*** for identifying hospitalisations positive for pathogen ***p*** was given by


$${PPV}_{ap}=\frac{n_{ap}}{n_{a}.}$$

Here, *n_a._* refers to all hospitalisations for which a specific ICD-10 code ***a*** had been assigned, regardless of their laboratory results. Both Se and PPV were expressed as percentages (Table 2).

**Table 2:** Two-by-two table for assessing the accuracy of single-code and code combinations against a laboratory-defined reference standard, SARI surveillance, Denmark, Iceland, Malta, Norway, Spain, week 21/2021–39/2023

ICD-10 code(s) under assessment	Reference standard		Total
	Hospitalisations positive for pathogen p (*p*) (pathogen present)	Hospitalisations negative for pathogen p (*p’*) (pathogen absent)	
Hospitalisations with ICD-10 code(s) under assessment (case definition a (*a*) met)	*n_ap_*	*n_ap’_*	*n_a. = _n_ap + _n_ap’_*
Hospitalisations without ICD-10 code(s) under assessment (case definition a (*a’*) unmet)	*n_a’p_*	*n_a’p’_*	*n_a’. = _n_a’p + _n_a’p’_*
**Total**	*n_.p = _n_ap + _n_a’p_*	*n_.p’ = _n_ap’ + _n_a’p’_*	*n_a. + _n_a’. = _n_.p + _n_.p’_*

*a’* and *p’* represent the complementary of *a* and *p*, respectively. *n_ap_*: true positive cases, *n_ap’_*: false positive cases, *n_a’p_*: false negative cases, *n_a’p’_*: true negative cases; *n_a._*: sum of *n_ap_* and *n_ap’_*; *n_a’_*_._: sum of *n_a’p_* and *n_a’p’_*_,_ *n_.p_*: sum of *n_ap_* and *n_a’p_*, *n_.p’_*: sum of *n_ap’_* and *n_a’p’_*.

For each single ICD-10 code ***a***, we assigned Se and PPV ranks for the identification of pathogen ***p***. Codes with higher Se received lower Se ranks *R(Se_ap_)*, and codes with higher PPV received lower PPV ranks *R(PPV_ap_)*, and vice versa. Then, for each ICD-10 code ***a,*** we calculated a score based on the rank sums of Se and PPV to identify pathogen ***p***, defined as


$${Score}_{ap}=R\left({Se}_{ap}\right)+R\left({PPV}_{ap}\right)$$

We considered that the lower the Score_ap,_ the higher the ability of a code to maximise Se and PPV for identifying pathogen ***p***. If two codes achieved the same score, the code associated with the higher *n_ap_* was prioritised.

We then determined the 10 ICD-10 codes with the lowest Score*_ap_* for each pathogen ***p***. These were sequentially combined according to their rank order to formulate 10 cumulative pathogen-specific SARI case definitions (SARI-*p*) consisting of one to 10 diagnosis codes. The first SARI-*p* case definition included the single ICD-10 code ***a*** with the lowest score, and the last SARI-*p* case definition included the 10 ICD-10 codes with the lowest scores.

#####  -2- Case-definition accuracy assessment

We calculated the Se and PPV of the 10 ICD-10 code-based case definitions developed as described above to identify a specific pathogen or any of the three. In a similar manner as with single codes, the Se and PPV of case definitions were given by


$${Se}_{ap}=\frac{n_{ap}}{n_{.p}\;\;}\;\;and\;\;{PPV}_{ap}=\frac{n_{ap}}{n_{a}.}$$

However, here n_ap_ or TP represent hospitalisation episodes containing at least one of the codes in the case definition ***a*** with laboratory confirmation, and *n_a._* refers to all hospitalisations assigned at least one of the codes in the case definition ***a***, regardless of their laboratory results (Table 2).

#####  -3- Case-definition selection

We selected case definitions that maximised the clinical utility index (CUI = Se × PPV). We categorised the CUI based on the score as excellent (≥ 0.81), good (0.64–0.80), satisfactory (0.49–0.63) or poor (< 0.49) utility [16,17]. In instances where two case definitions achieved the same CUI, the one incorporating fewer codes was chosen.

We based our analysis on the Se and PPV due to the following considerations: the Se and specificity (Sp) are measures of accuracy concerning the occurrence of TP and true negative (TN) cases, respectively. The Se quantifies the capability of a case definition to detect a condition or pathogen when it is indeed present. Meanwhile, the PPV and the negative predictive value (NPV) are accuracy measures of discrimination that help ascertain the actual status (laboratory result) of, respectively, cases and non-cases classified by the case definition. The PPV provides the proportion of TP among cases identified by the case definitions. Therefore, case definitions with high Se and PPV enhance the detection of TP cases while reducing the number of FP and are preferable for case finding. Consequently, we considered that the higher the CUI, the higher the suitability of a code set for finding SARI cases associated with specific pathogens [16,18].

#### Other considerations

Denmark, Malta, Norway and Spain completed the above analysis. However, Iceland's data did not include RSV testing results, which limited their analysis to SARS-CoV-2 and influenza virus.

Stratification was performed to identify heterogeneity due to patient characteristics (age groups: 0–4, 5–14, 15–64 and ≥ 65 years), and high or low SARI activity. The period of high SARI activity included all weeks where the number of SARI admissions per 100,000 population exceeded the 66th percentile of the admissions within the study period, as defined by each country. Weeks with admissions below this threshold formed the low-activity period. A description of periods by SARI activity and country is appended in Supplementary Table S2.

## Results

### Study population and testing results

#### Study population

A total of 564,584 hospitalisation episodes met the ICD-10 code selection criteria and were therefore included. Norway and Denmark contributed the largest number of hospitalisations, representing 48.0% (n = 271,097) and 42.0% (n = 237,208) of the total, respectively. Spain, Malta and Iceland provided smaller proportions, contributing 6.2% (n = 35,161), 2.5% (n = 14,066) and 1.2% (n = 7,052) of the hospitalisation episodes, respectively (Table 3).

**Table 3:** Testing status and testing results for hospitalisations with respiratory disease and COVID-19 ICD-10 codes, Denmark, Iceland, Malta, Norway, Spain, week 21/2021–week 39/2023

Country	Hospitalisations meeting inclusion criteria^a^	n tested for at least one pathogen^b^	SARS-CoV-2				Influenza virus				RSV				SARS-CoV-2, influenza, RSV			
			Tested (among hospitalised)		Test-positive (among tested)		Tested (among hospitalised)		Test-positive (among tested)		Tested (among hospitalised)		Test-positive (among tested)		Tested for all three (among hospitalised)		Test-positive for at least one (among tested)	
			n	%	n	%	n	%	n	%	n	%	n	%	n	%	n	%
Denmark	237,208	179,051	176,319	74.3	32,832	18.6	134,842	56.8	6,522	4.8	71,563	30.2	6,680	9.3	68,594	28.9	19,237	28.0
Iceland	7,052	4,428	4,268	60.5	1,632	38.2	1,834	26.0	179	9.8	NA				NA			
Malta	14,066	11,967	9,503	67.6	2,226	23.4	6,950	49.4	403	5.8	6,950	49.4	321	4.6	4,486	31.9	711	15.9
Norway	271,097	177,884	176,593	65.1	37,918	21.5	159,354	58.8	8,908	5.6	112,802	41.6	8,453	7.5	111,687	41.2	34,301	30.7
Spain	35,161	28,976	28,480	81.0	6,435	22.6	10,438	29.7	1,677	16.1	1,621	4.6	280	17.3	1,584	4.5	649	41.0
**Total**	**564,584**	**402,306**	**395,163**	**70.0**	**81,043**	**20.5**	**313,418**	**55.5**	**17,689**	**5.6**	**192,936**	**34.2**	**15,734**	**8.2**	**186,351**	**33.0**	**54,898**	**29.5**

ICD-10: 10th edition of the International Classification of Disease codes; NA: not available; RSV: respiratory syncytial virus; SARS-CoV-2: severe acute respiratory syndrome coronavirus 2.

^a^ Hospitalisations with respiratory disease ICD-10 codes: J00–J99 or COVID-19 ICD-10 codes: U07.1, U07.2, and Danish codes B34.2A, B97.2A, Z03.8PA1.

^b^ Number tested for at least one of SARS-CoV-2, influenza virus or RSV.

#### Proportion tested

Among all 564,584 hospitalisations, 71.3% (n = 402,306) had a test reported for at least one of the pathogens under consideration, 70.0% (n = 395,163) had a test reported for SARS-CoV-2, 55.5% (n = 313,418) had a test for influenza virus, 34.2% (n = 192,936) for RSV, and 33.0% (n = 186,351) for all three pathogens. The proportion tested for SARS-CoV-2 ranged from 60.5% (Iceland) to 81.0% (Spain), while the proportion tested for influenza virus ranged from 26.0% (Iceland) to 58.8% (Norway), and for RSV from 4.6% (Spain) to 49.4% (Malta). The proportion tested for all three viruses ranged from 4.5% (Spain) to 41.2% (Norway) (Table 3). In Supplementary Table S3, we append additional information on the proportion tested and the test positivity by SARI activity and age group.

#### Test positivity

Among hospitalisations with reported laboratory tests, 20.5% (81,043/395,163), 5.6% (17,689/313,418) and 8.2% (15,734/192,936) tested positive for SARS-CoV-2, influenza virus, and RSV, respectively. For SARS-CoV-2, the test positivity ranged from 18.6% (Denmark) to 38.2% (Iceland), while for influenza virus the test positivity ranged from 4.8% (Denmark) to 16.1% (Spain), and for RSV from 4.6% (Malta) to 17.3% (Spain). Among hospitalisations from patients tested for all three pathogens, 29.5% (54,898/186,351) had laboratory confirmation for at least one of the pathogens, ranging from 15.9% (Malta) to 41.0% (Spain) (Table 3). 

### Case definition accuracy assessment

Tables 4 and 5 show overall and stratified accuracy results for case definitions that achieved the highest CUI, respectively. Supplementary Table S4 provides the ICD-10 codes selected to build pathogen-specific SARI case definitions, along with the Se, PPV and CUI of the 10 case definitions assessed for each pathogen and stratum.

**Table 4:** Accuracy of the ICD-10 code-based case definitions that maximise the sensitivity and positive predictive value in identifying SARI associated with SARS-CoV-2, influenza virus, RSV or any of the three viruses, Denmark, Iceland, Malta, Norway, Spain, week 21/2021–week 39/2023

All ages	ICD-10^a^							TP + FN	TP + FP	Se (%)^b^	PPV (%)^c^	CUI^d^	CUI score^e^
	U07.1	J09	J10	J11	J12	J20	J21						
	B34.2A+B97.2A												
SARI-SARS-CoV-2													
Denmark	✓	✗						32,832	31,027	90.2	95.4	0.86	E
Iceland	✓							1,632	1,943	96.7	81.2	0.79	G
Malta	✓							2,226	2,246	91.1	90.2	0.82	E
Norway	✓							37,918	33,973	88.4	98.7	0.87	E
Spain	✓							6,435	6,482	95.7	95.0	0.91	E
SARI-influenza													
Denmark	✗	✓	✓	✓	✗			6,522	5,612	84.5	98.1	0.83	E
Iceland		✓	✓	✓				179	214	71.5	59.8	0.43	P
Malta		✗	✓	✓				403	240	55.6	93.3	0.52	S
Norway		✓	✓	✓				8,908	8,449	91.6	96.5	0.88	E
Spain		✓	✓	✗				1,677	1,599	93.7	98.3	0.92	E
SARI-RSV													
Denmark	✗				✓	✓	✓	6,680	6,805	58.9	57.8	0.34	P
Malta					✗	✗	✓	321	228	34.6	48.7	0.17	P
Norway					✗	✗	✓	8,453	4,550	38.2	71.1	0.27	P
Spain					✗	✗	✓	280	238	49.6	58.4	0.29	P
SARI-any of the three pathogens													
Denmark	✓	✓	✓	✗	✓	✓	✓	19,237	18,105	79.5	84.5	0.67	G
Malta	✓	✗	✓	✓	✗	✗	✓	711	637	69.8	77.9	0.54	S
Norway	✓	✗	✓	✓	✓	✗	✓	34,301	33,442	83.5	85.6	0.71	G
Spain	✓	✗	✓	✗	✓	✗	✓	649	638	78.9	80.3	0.63	S

✓: The ICD-10 code maximises the sensitivity and positive predictive value in identifying SARI associated with the pathogen under assessment; ✗: The ICD-10 code does not maximise the sensitivity and positive predictive value in identifying SARI associated with the pathogen under assessment; CUI: clinical utility index; FN: false negative; FP: false positive; ICD-10: 10th edition of the International Classification of Disease; PPV: positive predictive value; RSV: respiratory syncytial virus; SARI: severe acute respiratory infection; SARS-CoV-2: severe acute respiratory syndrome coronavirus 2; Se: sensitivity; TP: true positive.

^a^ ICD-10 codes; U07.1: COVID-19, virus identified. B34.2A: COVID-19, not elsewhere classified (Denmark-specific code). B97.2A: COVID-19 severe acute respiratory syndrome (Denmark-specific code). J09: Influenza due to identified zoonotic or pandemic influenza virus. J10: Influenza due to identified seasonal influenza virus. J11: Influenza due to unidentified influenza virus. J12: Viral pneumonia, not elsewhere classified. J20: Acute bronchitis. J21: Acute bronchiolitis.

^b^ Se(%) = TP/(TP + FN) × 100.

^c^ PPV(%) = TP/(TP + FP) × 100.

^d^ CUI = Se × PPV.

^e^ CUI score utilises the following qualitative categories; E: excellent utility; G: good utility; S: satisfactory utility; P: poor utility.

SARI-pathogen refers to SARI associated with a specific pathogen (SARS-CoV-2, influenza virus, RSV or any of the three).

**Table 5:** Accuracy of the ICD-10 code-based case definitions that maximise the sensitivity and positive predictive value in identifying SARI cases associated with SARS-CoV-2, influenza virus, RSV or any of the three pathogens, by SARI activity and age group, Denmark, Iceland, Malta, Norway, Spain, week 21/2021–week 39/2023

Country	SARI-SARS-CoV-2					SARI-influenza					SARI-RSV					SARI-any of the three pathogens				
	ICD-10^a^	Se^b^ (%)	PPV^c^ (%)	CUI^d^		ICD-10^a^	Se^b^ (%)	PPV^c^ (%)	CUI^d^		ICD-10^a^	Se^b^ (%)	PPV^c^ (%)	CUI^d^		ICD-10^a^	Se^b^ (%)	PPV^c^ (%)	CUI^d^	
Period of high SARI activity																				
Denmark	B34,B97	91.3	95.3	0.87	E	J09, J10	85.1	98.5	0.84	E	J12, J20, J21	55.5	61.0	0.34	P	B34, B97, J09, J10, J12, J20, J21	79.4	86.4	0.69	G
Iceland	U07.1	97.1	78.4	0.76	G	J09, J10, J11	77.0	60.5	0.47	P	NA					NA				
Malta	U07.1	92.4	91.3	0.84	E	J10, J11	60.5	94.9	0.57	S	J21	17.3	26.0	0.05	P	U07.1, J10, J11	70.5	86.6	0.61	S
Norway	U07.1	88.5	98.8	0.87	E	J09, J10, J11	92.4	97.6	0.90	E	J12, J20, J21	82.8	46.3	0.38	P	U07.1, J10, J12, J21	82.4	90.5	0.75	G
Spain	U07.1	96.5	95.0	0.92	E	J09, J10	94.7	97.8	0.93	E	J21	51.7	68.2	0.35	P	U07.1, J10, J12, J21	78.4	84.4	0.66	G
Period of low SARI activity																				
Denmark	B34,B97	88.5	95.6	0.85	E	J09, J10, J11	82.7	98.1	0.81	E	J12, J20, J21	63.4	54.6	0.35	P	B34, B97, J09, J12, J21	72.7	92.8	0.67	G
Iceland	U07.1	95.9	86.9	0.83	E	J09, J10, J11	54.5	57.1	0.31	P	NA					NA				
Malta	U07.1	90.7	90.0	0.82	E	J10, J11	53.4	92.5	0.49	S	J21	39.8	55.1	0.22	P	U07.1, J10, J11, J21	68.5	76.9	0.53	S
Norway	U07.1	88.4	98.6	0.87	E	J09, J10, J11	89.7	94.1	0.84	E	J12, J21	64.3	20.7	0.13	P	U07.1, J10, J12, J21	83.0	78.4	0.65	G
Spain	U07.1	94.7	95.1	0.90	E	J09, J10, J11	95.4	98.7	0.94	E	J21	39.6	30.6	0.12	P	U07.1, J10, J12	69.6	88.1	0.61	S
Age 0–4 years																				
Denmark	B34	82.9	97.8	0.81	E	J09, J10	74.5	95.2	0.71	G	J12, J20, J21, J96	81.3	55.4	0.45	P	B34, J09, J10, J12, J21	62.5	82.8	0.52	S
Iceland	U07.1	95.6	95.6	0.91	E	U07.1, J09, J10, J91	81.0	51.5	0.42	P	NA					NA				
Malta	U07.1	95.4	96.9	0.93	E	J10	46.2	80.0	0.37	P	J21	71.9	51.0	0.37	P	U07.1, J21	76.2	60.6	0.46	P
Norway	U07.1	86.4	98.6	0.85	E	J09, J10, J11	88.0	89.2	0.79	G	J12, J21	79.8	63.7	0.51	S	U07.1, J10, J21	74.2	80.8	0.60	S
Spain	U07.1	89.6	89.6	0.80	G	J10	91.0	98.4	0.90	E	J12, J21	67.1	60.4	0.41	P	U07.1, J10, J12, J21	72.6	67.1	0.49	P
Age 5–14 years																				
Denmark	B34,B97	86.4	92.5	0.80	G	J09, J10	78.5	95.2	0.75	G	J21	11.9	66.7	0.08	P	J09, B34, J10, B97	66.8	93.2	0.62	S
Iceland	U07.1	95.6	95.6	0.91	E	J03, J04, J10, J11	71.4	62.5	0.45	P	NA					NA				
Malta	U07.1	100	100	1.0	E	J10, J11	57.9	100.0	0.58	S	J06, J18	75.0	14.3	0.11	P	U07.1, J06, J10, J11, J21, J18	87.5	63.6	0.56	S
Norway	U07.1	84.5	98.0	0.83	E	J09, J10, J11	91.3	96.9	0.89	E	J12, J20, J21	61.0	24.1	0.15	P	U07.1, J10, J11	71.0	97.4	0.69	G
Spain	U07.1	83.3	71.4	0.60	S	J10	81.8	94.7	0.78	G	J12	50.0	50.0	0.25	P	U07.1, J10, J12, J95, J99	64.3	90.0	0.58	S
Age 15–64 years																				
Denmark	B34,B97	92.4	89.3	0.83	E	J09, J10	82.4	98.9	0.82	E	J12, J21	23.1	55.4	0.13	P	J09, B34, B97, J10, J12	80.5	95.1	0.77	G
Iceland	U07.1	97.4	86.5	0.84	E	J09, J10, J11	70.2	62.3	0.44	P	NA					NA				
Malta	U07.1	92.6	92.8	0.86	E	J10, J11	58.1	94.4	0.55	S	J21	10.3	50.0	0.05	P	U07.1, J10, J11	71.6	85.0	0.61	S
Norway	U07.1	91.1	98.5	0.90	E	J09, J10, J11	92.5	96.7	0.90	E	J12, J20, J21	68.2	18.5	0.13	P	U07.1, J10	77.3	98.1	0.76	G
Spain	U07.1	96.9	93.1	0.90	E	J09, J10, J11	95.0	99.0	0.94	E	J98	50.0	29.4	0.15	P	U071, J10, J12	85.4	88.6	0.76	G
Age ≥ 65 years																				
Denmark	B34,B97	89.6	98.2	0.88	E	J09, J10	86.4	99.0	0.86	E	J12, J20, J21	27.1	58.9	0.16	P	B34, B97, J09, J10, J12	78.3	96.6	0.76	G
Iceland	U07.1	96.3	77.7	0.75	G	J09, J10, J11	74.0	59.7	0.44	P	NA					NA				
Malta	U07.1	89.7	88.3	0.79	G	J10, J11	54.8	93.6	0.51	S	J14, J20, J21, J22, J40, J44, J45, J60	73.5	4.1	0.03	P	U07.1, J10, J11	66.3	83.0	0.55	S
Norway	U07.1	87.4	98.9	0.86	E	J09, J10, J11	91.5	97.7	0.89	E	J12, J20, J21	74.1	22.0	0.16	P	U07.1, J10, J12	82.0	90.7	0.74	G
Spain	U07.1	95.6	95.8	0.92	E	J09, J10	94.9	98.2	0.93	E	J98	46.9	15.5	0.07	P	U071, J10, J12	83.0	93.5	0.78	G

CUI: clinical utility index; FN: false negative; FP: false positive; ICD-10: 10th edition of the International Classification of Disease codes; PPV: positive predictive value; RSV: respiratory syncytial virus; SARI: severe acute respiratory infection; SARS-CoV-2: severe acute respiratory syndrome coronavirus 2; Se: sensitivity; TP: true positive.

^a^ ICD-10 codes; U07.1: COVID-19, virus identified. B34.2A: COVID-19, not elsewhere classified (Denmark-specific code). B97.2A: COVID-19 severe acute respiratory syndrome (Denmark-specific code). J03: Acute tonsillitis. J04: Acute laryngitis and tracheitis. J06: Acute upper respiratory infections of multiple and unspecified sites. J09: Influenza due to identified zoonotic or pandemic influenza virus. J10: Influenza due to identified seasonal influenza virus. J11: Influenza due to unidentified influenza viruses. J12: Viral pneumonia, not elsewhere classified. J14: Pneumonia due to *Haemophilus influenzae*. J15: Bacterial pneumonia, not elsewhere classified. J16: Chlamydial pneumonia. J18: Pneumonia, unspecified organism. J20: Acute bronchitis. J21: Acute bronchiolitis. J22: Unspecified acute lower respiratory infection. J40: Bronchitis, not specified as acute or chronic. J44: Other chronic obstructive pulmonary disease. J45: Asthma. J60: Coalworker's pneumoconiosis. Asthma. J91: Pleural effusion in conditions classified elsewhere. J95: Postprocedural respiratory disorders, not elsewhere classified. J96: Respiratory failure, not elsewhere classified. J98: Other respiratory disorders. J99: Respiratory disorders in diseases classified elsewhere.

^b^ Se (%) = TP/(TP + FN) × 100.

^c^ PPV (%) = TP/(TP + FP)  × 100.

^d^ CUI = Se × PPV; results are provided both quantitatively and qualitatively, using the following categories: E: excellent utility, G: good utility, S: satisfactory utility, P: poor utility.

#### SARI associated with SARS-CoV-2

The best accuracy for identifying SARI-SARS-CoV-2 cases was achieved by a case definition that included only U07.1 (‘COVID-19, virus identified’) in all countries, except Denmark, where B34.2A (‘COVID-19, not elsewhere classified’) and B97.2A (’COVID-19 severe acute respiratory syndrome’) were the best. These codes yielded excellent utility across most countries and strata (Se ≥ 88%, PPV ≥ 90%, CUI ≥ 0.82 for the all-ages group in Denmark, Malta, Norway and Spain). Good utility was reported for U07.1 in the ≥ 65-year-old group, during periods of high SARI activity, and the all-ages group in Iceland. In Denmark, B34.2A and B97.2A also achieved good utility for the 5–14-year-old group. In Spain, U07.1 yielded good and satisfactory utility for the 0–4-year-old and the 5–14-year-old groups, respectively.

#### SARI associated with influenza virus

Case definitions including codes J09 (‘Influenza due to identified zoonotic or pandemic influenza virus’), J10 (‘Influenza due to identified seasonal influenza virus’), and J11 (‘Influenza, virus not identified’) yielded the best accuracy results for SARI-influenza case finding across all countries and strata. For the all-ages group, the key codes were J09 and J10 in Spain, J10 and J11 in Malta, and J09, J10 and J11 in Denmark, Norway and Iceland. This suggests that admissions with at least one of these codes correspond to SARI-influenza. In three countries (Denmark, Norway and Spain), the utility achieved across strata was generally excellent (Se ≥ 85%, PPV ≥ 97%, CUI ≥ 0.83 in the all-ages group), with some exceptions among patients younger than 15 years, where the utility was good. Lower accuracy was observed in Iceland (poor utility across all strata) and Malta (poor utility for the 0–4-year-old group and satisfactory utility for the rest of strata).

#### SARI associated with RSV

For SARI-RSV, the most accurate case definitions across countries included J12 (‘Viral pneumonia, not elsewhere classified’), J20 (‘Acute bronchitis’), J21 (‘Acute bronchiolitis’), and J98 (‘Other respiratory disorders’). For the all-ages group, the key codes were J21 in Malta, Norway and Spain, and J12, J20 and J21 in Denmark. Other relevant case definitions for specific strata included J96 (0–4 age group in Denmark), J98 (‘Other respiratory disorders’) for the 15–64 and the ≥ 65 age groups in Spain, and J14 (‘Pneumonia due to *Haemophilus influenzae*’), J22 (‘Unspecified acute lower respiratory infection’) and J45 (‘Asthma’) for the ≥ 65 age group in Malta. However, the utility of all code combinations across countries and strata was generally poor (Se: 35–59%, PPV: 49–71%, CUI: 0.17–0.34 in the all-ages group), except for codes J12 and J21 among children aged 0–4 years in Norway, which achieved satisfactory utility.

#### SARI associated with any of the three pathogens

For SARI associated with SARS-CoV-2, influenza virus or RSV, the most accurate case definition included U07.1, B34.2A, B97.2A (previously identified for SARS-CoV-2), J09, J10, J11 (identified for influenza virus), and J12, J20, J21 (for RSV). The specific combinations of these codes varied by country. Overall, the utility ranged from satisfactory (Se ≥ 70%, PPV ≥ 78%, CUI ≥ 0.54 for the all-ages group in Malta and Spain) to good (Se ≥ 80%, PPV ≥ 85%, CUI ≥ 0.67 for the all-ages group in Denmark and Norway). However, utility was poor for the 0–4-year-old group in Malta and Spain.

## Discussion

Our accuracy assessment identified similar case definitions across countries, with overall satisfactory to excellent utility for SARI associated with SARS-CoV-2 and influenza virus, while accuracy was lower for SARI associated with RSV. For SARI-SARS-CoV-2, a case definition including the four-character COVID-19-specific U07.1 code (B34.2A and B97.2A in Denmark) assigned at admission and discharge provided good to excellent utility across countries. In Canada, studies demonstrated that U07.1 code at discharge indicated excellent utility when using PCR laboratory results as the reference [19,20]. For SARI-influenza, case definitions combining three-character discharge codes J09, J10 and J11 achieved satisfactory to excellent utility, with J10 common to all countries. However, unlike the other countries, utility was poor in Iceland, which may be due to the inclusion of codes at admission as well as at discharge. A similar combination of discharge codes provided results corresponding to good utility in another Canadian study [21]. For SARI-RSV, the key three-character codes were J12, J20 and J21, with J21 common to all countries. However, the overall utility was poor. In Canada, a combination of four-character RSV-specific respiratory and non-respiratory disease codes provided results corresponding to good utility [21]. Furthermore, a Spanish study found that four-character respiratory disease code combinations, including RSV-specific codes, achieved satisfactory utility [22]. In our study, using three-character codes (i.e. non-RSV specific) probably increased Se and mostly decreased PPV, thereby reducing the CUI. Exploring four-character codes for SARI-RSV is therefore recommended. Finally, for SARI associated with any of the three pathogens, a combination of the codes identified for each one achieved satisfactory to good utility, with countries sharing U07.1 (B34.2A + B97.2A in Denmark), J10 and J21.

Importantly, codes with poor utility may still hold value since the qualitative CUI interpretation has not been verified for surveillance and several CUI classifications exist [16,18]. One of them considers CUI values in the range 0.41–0.60 to be of moderate utility, meaning that Iceland’s results for SARI-influenza would correspond to moderate rather than poor utility. Furthermore, Davis et al. [23] found that the accuracy of the WHO’s 2014 SARI case definition [2] against PCR-confirmed influenza virus and RSV among hospitalised patients yielded accuracy results that corresponded to poor utility [2,23]. To understand the suitability of the identified codes for trend monitoring, we recommend assessing whether weekly rates of pathogen-specific SARI can identify epidemic trends and periods, as defined by weekly test positivity from an external data source.

Cross-country accuracy variations could be attributed to differences in surveillance systems, which we have reported here. These include differences in definitions of hospital admission and readmission, the use of ICD-10 codes assigned at admission or discharge, record selection processes, time windows for laboratory confirmation, and testing practices. Collectively, these factors contribute to the heterogeneity of national surveillance systems, impacting study participants across countries. This encompasses the characteristics of the reference standard groups, defined by specific ICD-10 codes and available laboratory test results. For instance, since patients without laboratory testing were excluded from the accuracy assessment, settings testing only the most severe cases may exhibit higher test positivity, leading to higher PPV and CUI. Moreover, the availability of laboratory results for influenza virus has been shown to improve the accuracy of ICD-10 coding [24]. Since admission codes are less likely to be informed by laboratory results, they may yield lower accuracy than discharge codes, potentially explaining Iceland's notably lower accuracy for SARI-influenza. The overall effect of these factors on achieved accuracy is unknown, adding complexity to cross-country data interpretation. However, similar codes maximised the Se and PPV across countries.

A limitation of our analysis was that data were retrospectively extracted. As a result, the retained codes primarily represent consolidated discharge diagnoses, which may differ from admission diagnosis codes that could be available and used for real-time trend monitoring [25,26]. Conducting this analysis using weekly data extractions or timestamped information on when codes were assigned would help assess the accuracy of ICD-10 code-based case definitions for real-time surveillance. The lack of consistently structured symptom data in EHR databases impeded their use for defining a SARI reference standard. Instead, we used hospitalisation episodes with respiratory disease or COVID-19 ICD-10 codes and laboratory results for SARS-CoV-2, influenza virus or RSV. True positive SARI pathogen-specific cases were those with laboratory confirmation. This definition was deemed the closest approximation to SARI associated with the pathogens under assessment as it integrates laboratory and clinical information. However, patients not experiencing clinically defined SARI may have been misclassified as true positive pathogen-specific SARI cases (e.g. patients with SARS-CoV-2 confirmation and an unrelated respiratory disease). In addition, we may have missed SARI patients who did not receive respiratory disease or COVID-19 codes but instead were given other codes, such as those for related complications (e.g. hospitalisations with a cardiovascular diagnosis code associated with influenza infection) [11]. Nevertheless, we assume that including non-respiratory disease codes would be unlikely to improve our results due to their expected low PPV. We employed a simplified process to build case definitions, which may be improved by other approaches such as combining more than 10 codes per pathogen, although these may be more burdensome. A Spanish study examined all possible code combinations of a more restricted set of ICD-10 codes for COVID-19 and respiratory diseases presenting seasonal variability, which yielded results similar to ours, notably for SARI-SARS-CoV-2 and SARI-influenza [22]. Lastly, our study period covered much of the COVID-19 pandemic, which modified the epidemiology of SARI, possibly hospital coding and testing practices, and may have influenced our results [1,11].

## Conclusions

Our study applied a methodology for constructing and assessing case definitions based on ICD-10 codes that maximises the Se and PPV for identifying SARI associated with specific pathogens. We found similar diagnosis code sets for SARI associated with SARS-CoV-2, influenza virus, RSV and any of the three pathogens across five countries, despite variations in accuracy level. We recommend replicating this assessment in countries implementing EHR-based SARI surveillance using ICD-10 codes, preferably during non-pandemic periods related to SARI.

## Data Availability

See Supplementary Table S4. Additional data may be provided upon request.

## Supplementary Data

1338000 Supplement
