# Supplementary material for: Surveillance of severe acute respiratory infections associated with SARS-CoV-2, influenza virus and RSV using ICD-10 codes: a case definition accuracy study across five European countries, 2021 to 2023
Source: Euro Surveill. 2025 Jul 10;30(27):2400748. doi: 10.2807/1560-7917.ES.2025.30.27.2400748 (PMC12262114; doi:10.2807/1560-7917.ES.2025.30.27.2400748)
Supplement: Supplement [file 24-00748_SANCHEZ_RUIZ_Supplement.pdf]

Supplementary materials

This supplementary material is hosted by Eurosurveillance as supporting information alongside the article [Surveillance of severe acute respiratory infections associated with SARS-CoV-2, influenza virus and RSV using ICD-10 codes: a case definition accuracy study across five European countries, 2021 to 2023], on behalf of the authors, who remain responsible for the accuracy and appropriateness of the content. The same standards for ethics, copyright, attributions and permissions as for the article apply. Supplements are not edited by Eurosurveillance and the journal is not responsible for the maintenance of any links or email addresses provided therein.

Supplementary Text 1–ICD-10 (version 2019) codes [1]

COVID-19 specific codes

Codes for special purposes

- U07.1 COVID-19, virus identified
- U07.2 COVID-19, virus not identified

Denmark- specific codes (not available at WHO)

- B97.2A: COVID-19 severe acute respiratory syndrome
- B34.2A: COVID-19, not elsewhere classified
- Z03.8PA1: Observation due to suspicion of COVID-19 infection
    - Note: Denmark-specific codes are not standard WHO ICD-10 codes

Diseases of the respiratory system

Acute upper respiratory infections (J00-J06)

- **J00**: Acute nasopharyngitis [common cold]
- **J01**: Acute sinusitis
    - **J01.0**: Acute maxillary sinusitis
    - **J01.1**: Acute frontal sinusitis
    - **J01.2**: Acute ethmoidal sinusitis
    - **J01.3**: Acute sphenoidal sinusitis
    - **J01.4**: Acute pansinusitis
    - **J01.8**: Other acute sinusitis
    - **J01.9** : Acute sinusitis, unspecified
- **J02**: Acute pharyngitis
    - **J02.0**: Streptococcal pharyngitis
    - **J02.8**: Acute pharyngitis due to other specified organisms
    - **J02.9**: Acute pharyngitis, unspecified
- **J03**: Acute tonsillitis
    - **J03.0**: Streptococcal tonsillitis
    - **J03.8**: Acute tonsillitis due to other specified organisms
    - **J03.9**: Acute tonsillitis, unspecified
- **J04**: Acute laryngitis and tracheitis
    - **J04.0**: Acute laryngitis
    - **J04.1**: Acute tracheitis
    - **J04.2**: Acute laryngotracheitis
- **J05**: Acute obstructive laryngitis [croup] and epiglottitis
    - **J05.0**: Acute obstructive laryngitis [croup]
    - **J05.1**: Acute epiglottitis
- **J06**: Acute upper respiratory infections of multiple and unspecified sites
    - **J06.0**: Acute laryngopharyngitis
    - **J06.8**: Other specified acute upper respiratory infections
    - **J06.9**: Acute upper respiratory infection, unspecified

Influenza and pneumonia (J09-J18)

- **J09**: Influenza due to identified zoonotic or pandemic influenza virus
- **J10**: Influenza due to identified seasonal influenza virus
    - **J10.0**: Influenza with pneumonia, seasonal influenza virus identified
    - **J10.1**: Influenza with other respiratory manifestations, seasonal influenza virus identified
    - **J10.8**: Influenza with other manifestations, seasonal influenza virus identified

[1] World Health Organization. International Statistical Classification of Diseases and Related Health Problems 10th Revision [Internet]. Available from: https://icd.who.int/browse10/2019/en

- **J11**: Influenza, virus not identified
    - **J11.0**: Influenza with pneumonia, virus not identified
    - **J11.1**: Influenza with other respiratory manifestations, virus not identified
    - **J11.8**: Influenza with other manifestations, virus not identified
- **J12**: Viral pneumonia, not elsewhere classified
    - **J12.0**: Adenoviral pneumonia
    - **J12.1**: Respiratory syncytial virus pneumonia
    - **J12.2**: Parainfluenza virus pneumonia
    - **J12.3**: Human metapneumovirus pneumonia
    - **J12.8**: Other viral pneumonia
    - **J12.9**: Viral pneumonia, unspecified
- **J13**: Pneumonia due to Streptococcus pneumoniae
- **J14**: Pneumonia due to Haemophilus influenzae
- **J15**: Bacterial pneumonia, not elsewhere classified
    - **J15.0**: Pneumonia due to Klebsiella pneumoniae
    - **J15.1**: Pneumonia due to Pseudomonas
    - **J15.2**: Pneumonia due to staphylococcus
    - **J15.3**: Pneumonia due to streptococcus, group B
    - **J15.4**: Pneumonia due to other streptococci
    - **J15.5**: Pneumonia due to Escherichia coli
    - **J15.6**: Pneumonia due to other Gram-negative bacteria
    - **J15.7**: Pneumonia due to Mycoplasma pneumoniae
    - **J15.8**: Other bacterial pneumonia
    - **J15.9**: Bacterial pneumonia, unspecified
- **J16**: Pneumonia due to other infectious organisms, not elsewhere classified
    - **J16.0**: Chlamydial pneumonia
    - **J16.8**: Pneumonia due to other specified infectious organisms
- **J17***: Pneumonia in diseases classified elsewhere
    - **J17.0***: Pneumonia in bacterial diseases classified elsewhere
    - **J17.1***: Pneumonia in viral diseases classified elsewhere
    - **J17.2***: Pneumonia in mycoses
    - **J17.3***: Pneumonia in parasitic diseases
    - **J17.8*:** Pneumonia in other diseases classified elsewhere
      Note : Asterisks indicate conditions that are secondary to diseases classified elsewhere in the ICD-10 system.
- **J18**: Pneumonia, organism unspecified
    - **J18.0**: Bronchopneumonia, unspecified
    - **J18.1**: Lobar pneumonia, unspecified
    - **J18.2**: Hypostatic pneumonia, unspecified
    - **J18.8**: Other pneumonia, unspecified
    - **J18.9**: Pneumonia, unspecified

Other acute lower respiratory infections (J20-J22)

- **J20**: Acute bronchitis
    - **J20.0**: Acute bronchitis due to Mycoplasma pneumoniae
    - **J20.1**: Acute bronchitis due to Haemophilus influenzae
    - **J20.2**: Acute bronchitis due to streptococcus
    - **J20.3**: Acute bronchitis due to Coxsackievirus
    - **J20.4**: Acute bronchitis due to parainfluenza virus
    - **J20.5**: Acute bronchitis due to respiratory syncytial virus
    - **J20.6**: Acute bronchitis due to rhinovirus
    - **J20.7**: Acute bronchitis due to echovirus
    - **J20.8**: Acute bronchitis due to other specified organisms
    - **J20.9**: Acute bronchitis, unspecified
- **J21**: Acute bronchiolitis
    - **J21.0**: Acute bronchiolitis due to respiratory syncytial virus
    - **J21.1**: Acute bronchiolitis due to human metapneumovirus
    - **J21.8**: Acute bronchiolitis due to other specified organisms
    - **J21.9**: Acute bronchiolitis, unspecified
- **J22**: Unspecified acute lower respiratory infection

Other diseases of upper respiratory tract (J30-J39)

- **J30**: Vasomotor and allergic rhinitis
    - **J30.0**: Vasomotor rhinitis

[1] World Health Organization. International Statistical Classification of Diseases and Related Health Problems 10th Revision [Internet]. Available from: https://icd.who.int/browse10/2019/en

    - **J30.1**: Allergic rhinitis due to pollen
    - **J30.2**: Other seasonal allergic rhinitis
    - **J30.3**: Other allergic rhinitis
    - **J30.4**: Allergic rhinitis, unspecified
- **J31**: Chronic rhinitis, nasopharyngitis, and pharyngitis
    - **J31.0**: Chronic rhinitis
    - **J31.1**: Chronic nasopharyngitis
    - **J31.2**: Chronic pharyngitis
- **J32**: Chronic sinusitis
    - **J32.0**: Chronic maxillary sinusitis
    - **J32.1**: Chronic frontal sinusitis
    - **J32.2**: Chronic ethmoidal sinusitis
    - **J32.3**: Chronic sphenoidal sinusitis
    - **J32.4**: Chronic pansinusitis
    - **J32.8**: Other chronic sinusitis
    - **J32.9**: Chronic sinusitis, unspecified
- **J33**: Nasal polyp
    - **J33.0**: Polyp of nasal cavity
    - **J33.1**: Polypoid sinus degeneration
    - **J33.8**: Other polyp of sinus
    - **J33.9**: Nasal polyp, unspecified
- **J34**: Other disorders of nose and nasal sinuses
    - **J34.0**: Abscess, furuncle, and carbuncle of nose
    - **J34.1**: Cyst and mucocele of nose and nasal sinus
    - **J34.2**: Deviated nasal septum
    - **J34.3**: Hypertrophy of nasal turbinates
    - **J34.8**: Other specified disorders of nose and nasal sinuses
- **J35**: Chronic diseases of tonsils and adenoids
    - **J35.0**: Chronic tonsillitis
    - **J35.1**: Hypertrophy of tonsils
    - **J35.2**: Hypertrophy of adenoids
    - **J35.3**: Hypertrophy of tonsils with hypertrophy of adenoids
    - **J35.8**: Other chronic diseases of tonsils and adenoids
    - **J35.9**: Chronic disease of tonsils and adenoids, unspecified
- **J36**: Peritonsillar abscess
- **J37**: Chronic laryngitis and laryngotracheitis
    - **J37.0**: Chronic laryngitis
    - **J37.1**: Chronic laryngotracheitis
- **J38**: Diseases of vocal cords and larynx, not elsewhere classified
    - **J38.0**: Paralysis of vocal cords and larynx
    - **J38.1**: Polyp of vocal cord and larynx
    - **J38.2**: Nodules of vocal cords
    - **J38.3**: Other diseases of vocal cords
    - **J38.4**: Edema of larynx
    - **J38.5**: Laryngeal spasm
    - **J38.6**: Stenosis of larynx
    - **J38.7**: Other diseases of larynx
- **J39**: Other diseases of upper respiratory tract
    - **J39.0**: Retropharyngeal and parapharyngeal abscess
    - **J39.1**: Other abscess of pharynx
    - **J39.2**: Other diseases of pharynx
    - **J39.3**: Upper respiratory tract disease, unspecified
    - **J39.8**: Other specified diseases of upper respiratory tract
    - **J39.9**: Disease of upper respiratory tract, unspecified

Chronic lower respiratory diseases (J40-J47)

- **J40**: Bronchitis, not specified as acute or chronic
- **J41**: Simple and mucopurulent chronic bronchitis
    - **J41.0**: Simple chronic bronchitis
    - **J41.1**: Mucopurulent chronic bronchitis
    - **J41.8**: Mixed simple and mucopurulent chronic bronchitis
- **J42**: Unspecified chronic bronchitis
- **J43**: Emphysema
    - **J43.0**: MacLeod's syndrome
    - **J43.1**: Panlobular emphysema
    - **J43.2**: Centrilobular emphysema
    - **J43.8**: Other emphysema
    - **J43.9**: Emphysema, unspecified
- **J44**: Other chronic obstructive pulmonary disease
    - **J44.0**: Chronic obstructive pulmonary disease with acute lower respiratory infection
    - **J44.1**: Chronic obstructive pulmonary disease with acute exacerbation, unspecified
    - **J44.8**: Other specified chronic obstructive pulmonary disease
    - **J44.9**: Chronic obstructive pulmonary disease, unspecified
- **J45**: Asthma
    - **J45.0**: Predominantly allergic asthma
    - **J45.1**: Nonallergic asthma
    - **J45.8**: Mixed asthma
    - **J45.9**: Asthma, unspecified
- **J46**: Status asthmaticus
- **J47**: Bronchiectasis

Lung diseases due to external agents (J60-J70)

- **J60**: Coalworker's pneumoconiosis
- **J61**: Pneumoconiosis due to asbestos and other mineral fibers

[1] World Health Organization. International Statistical Classification of Diseases and Related Health Problems 10th Revision [Internet]. Available from: https://icd.who.int/browse10/2019/en

- **J62**: Pneumoconiosis due to dust containing silica
    - **J62.0**: Pneumoconiosis due to talc dust
    - **J62.8**: Pneumoconiosis due to other dust containing silica
- **J63**: Pneumoconiosis due to other inorganic dusts
    - **J63.0**: Aluminosis (of lung)
    - **J63.1**: Bauxite fibrosis (of lung)
    - **J63.2**: Berylliosis
    - **J63.3**: Graphite fibrosis (of lung)
    - **J63.4**: Siderosis
    - **J63.5**: Stannosis
    - **J63.8**: Pneumoconiosis due to other specified inorganic dusts
- **J64**: Unspecified pneumoconiosis
- **J65**: Pneumoconiosis associated with tuberculosis
- **J66**: Airway disease due to specific organic dust
    - **J66.0**: Byssinosis
    - **J66.1**: Flax-dresser's disease
    - **J66.2**: Cannabinosis
    - **J66.8**: Other airway disease due to specific organic dust
- **J67**: Hypersensitivity pneumonitis due to organic dust
    - **J67.0**: Farmer's lung
    - **J67.1**: Bagassosis
    - **J67.2**: Bird fancier's lung
    - **J67.3**: Suberosis
    - **J67.4**: Malt worker's lung
    - **J67.5**: Mushroom worker's lung
    - **J67.6**: Maple bark-stripper's lung
    - **J67.7**: Cheese washer's lung
    - **J67.8**: Hypersensitivity pneumonitis due to other organic dust
    - **J67.9**: Hypersensitivity pneumonitis, unspecified
- **J68**: Respiratory conditions due to inhalation of chemicals, gases, fumes, and vapors
    - **J68.0**: Bronchitis and pneumonitis due to chemicals, gases, fumes, and vapours
    - **J68.1**: Pulmonary edema due to chemicals, gases, fumes, and vapours
    - **J68.2**: Upper respiratory inflammation due to chemicals, gases, fumes and vapours, not elsewhere classified
    - **J68.3**: Other acute and subacute respiratory conditions due to chemicals, gases, fumes, and vapours
    - **J68.4**: Chronic respiratory conditions due to chemicals, gases, fumes, and vapors
    - **J68.8**: Other specified respiratory conditions due to chemicals, gases, fumes, and vapours
    - **J68.9**: Unspecified respiratory condition due to chemicals, gases, fumes and vapours
- **J69**: Pneumonitis due to solids and liquids
    - **J69.0**: Pneumonitis due to food and vomit
    - **J69.1**: Pneumonitis due to oils and essences
    - **J69.8**: Pneumonitis due to other solids and liquids
- **J70**: Respiratory conditions due to other external agents
    - **J70.0**: Acute pulmonary manifestations due to radiation
    - **J70.1**: Chronic and other pulmonary manifestations due to radiation
    - **J70.2**: Acute drug-induced interstitial lung disorders
    - **J70.3**: Chronic drug-induced interstitial lung disorders
    - **J70.4**: Drug-induced interstitial lung disorders, unspecified
    - **J70.8**: Other respiratory conditions due to other external agents
    - **J70.9**: Respiratory conditions due to unspecified external agent

Other respiratory diseases principally affecting the interstitium (J80-J84)

- **J80**: Adult respiratory distress syndrome (ARDS)
- **J81**: Pulmonary edema
- **J82**: Pulmonary eosinophilia, not elsewhere classified
- **J84**: Other interstitial pulmonary diseases
    - **J84.0**: Alveolar and parietoalveolar conditions
    - **J84.1**: Other interstitial pulmonary diseases with fibrosis
    - **J84.8**: Other specified interstitial pulmonary diseases
    - **J84.9**: Interstitial pulmonary disease, unspecified

Suppurative and necrotic conditions of the lower respiratory tract (J85-J86)

- **J85**: Abscess of lung and mediastinum
    - **J85.0**: Gangrene and necrosis of lung
    - **J85.1**: Abscess of lung with pneumonia

[1] World Health Organization. International Statistical Classification of Diseases and Related Health Problems 10th Revision [Internet]. Available from: https://icd.who.int/browse10/2019/en

- **J85.2**: Abscess of lung without pneumonia
- **J85.3**: Abscess of mediastinum

- **J86**: Pyothorax
  - **J86.0**: Pyothorax with fistula
  - **J86.9**: Pyothorax without fistula

Other diseases of pleura (J90-J94)

- **J90**: Pleural effusion, not elsewhere classified
- **J91***: Pleural effusion in conditions classified elsewhere
  - *Note : The asterisk indicates a condition that is secondary to diseases classified elsewhere in the ICD-10 system.*
- **J92**: Pleural plaque
  - **J92.0**: Pleural plaque with presence of asbestos
  - **J92.9**: Pleural plaques without asbestos
- **J93**: Pneumothorax
  - **J93.0**: Spontaneous tension pneumothorax
  - **J93.1**: Other spontaneous pneumothorax
  - **J93.8**: Other pneumothorax
  - **J93.9**: Pneumothorax, unspecified
- **J94**: Other pleural conditions
  - **J94.0**: Chylous pleural effusion
  - **J94.1**: Fibrothorax
  - **J94.2**: Haemothorax
  - **J94.8**: Other specified disorders of pleura conditions
  - **J94.9**: Pleural condition, unspecified

Other diseases of the respiratory system (J95-J99)

- **J95**: Postprocedural respiratory disorders, not elsewhere classified
  - **J95.0**: Tracheostomy malfunction
  - **J95.1**: Acute pulmonary insufficiency following thoracic surgery
  - **J95.2**: Acute pulmonary insufficiency following nonthoracic surgery
  - **J95.3**: Chronic pulmonary insufficiency following surgery
  - **J95.4**: Mendelson syndrome
  - **J95.5**: Postprocedural subglottic stenosis
  - **J95.8**: Other postprocedural respiratory disorders
  - **J95.9**: Postprocedural respiratory disorder, unspecified
- **J96**: Respiratory failure, not elsewhere classified
  - **J96.0**: Acute respiratory failure
  - **J96.1**: Chronic respiratory failure
  - **J96.9**: Respiratory failure, unspecified
- **J98**: Other respiratory disorders
  - **J98.0**: Diseases of bronchus, not elsewhere classified
  - **J98.1**: Pulmonary collapse
  - **J98.2**: Interstitial emphysema
  - **J98.3**: Compensatory emphysema
  - **J98.4**: Other disorders of lung
  - **J98.5**: Diseases of mediastinum, not elsewhere classified
  - **J98.6**: Disorders of diaphragm
  - **J98.7**: Respiratory infections, not elsewhere classified
  - **J98.8**: Other specified respiratory disorders
  - **J98.9**: Respiratory disorder, unspecified
- **J99***: Respiratory disorders in diseases classified elsewhere
  - **J99.0*:** Rheumatoid lung disease
  - **J99.1*:** Respiratory disorders in other diffuse connective tissue disorders
  - **J99.8* :** Respiratory disorders in other diseases classified elsewhere

  Note : Asterisks indicate conditions that are secondary to diseases classified elsewhere in the ICD-10 system.

[1] World Health Organization. International Statistical Classification of Diseases and Related Health Problems 10th Revision [Internet]. Available from: https://icd.who.int/browse10/2019/en

Table S1. Testing practices in countries participating in the study; Denmark, Iceland, Malta, Norway, Spain; week 21 of 2021 to week 39 of 2023.

Country	Selection of SARI cases for testing [1]	Testing approach [2]	Description
Denmark	At clinician's discretion for influenza virus and RSV<br><br>Mixed for SARS-CoV-2, varying from exhaustive selection to selection at the clinician's discretion over time	Mixed	In the beginning of the study period, extensive testing for SARS-CoV-2 was in place, including testing all patients with suspected COVID-19, all patients with an acute admission, and all asymptomatic patients with a planned admission expected to last at least 24 hours. As of March 2022, a gradual return to normal testing was implemented in the national testing strategy. However, all individuals showing symptoms of COVID-19 upon admission were still required to be tested, regardless of symptom severity. As of December 2022, the decision of testing individuals presenting with symptoms of COVID-19 upon admission were left at clinician's discretion. Guidelines recommended testing individuals where the result would have a clinical relevance or guide treatment decisions.<br><br>Most adult patients were tested in parallel for COVID-19 and influenza while most paediatric patients were tested in parallel for COVID-19 and RSV. Depending on local availability of equipment, some departments performed parallel testing for all three viruses on the adult population, but most commonly, adult patients were tested for RSV if the test for COVID-19 and influenza was negative.<br><br>RT-PCR tests were performed for all three pathogens: SARS-CoV-2, influenza virus, and RSV.
Iceland	At clinician's discretion	Parallel testing	Selection of patients tested for respiratory infections was based on clinician discretion for the largest duration of the study period.<br><br>RT-PCR tests for respiratory infections were performed at two laboratories in Iceland, the national reference laboratory at the National Hospital in Reykjavik and a smaller laboratory in the north of Iceland. The vast majority of the RT-PCR tests were performed at the National Hospital (approx. 90-95%) where parallel testing was used.<br><br>RT-PCR tests were performed for SARS-CoV-2 and influenza virus. Iceland did not contribute RSV results.
Malta	At clinician's discretion for influenza virus and RSV<br><br>Mixed for SARS-CoV-2, varying from exhaustive selection to selection at the clinician's discretion over time	Parallel testing for influenza and RSV<br><br>Mixed for SARS-CoV-2, varying from independent to parallel testing over time	RT-PCR tests for influenza virus and RSV were carried out using a multiplex kit at the clinician's discretion at the hospital level.<br><br>For SARS-CoV-2, RT-PCR tests were initially performed on all hospitalised patients at the beginning of the COVID-19 pandemic. However, these were gradually replaced by RATs, leading to a progressive decline in the number of patients tested, with selection increasingly based on the clinician's discretion. SARS-CoV-2 PCR testing dropped sharply around 2022-W22. By 2022-W37, fewer than 50% of hospitalised SARI patients were being tested using either RAT or PCR. From 2023-W12, parallel RT-PCR testing was used for all three pathogens, provided that swab samples allowed for it.<br><br>Among study participants tested for SARS-CoV-2: 82% received RT-PCR, 10% received RATs, and 8% underwent both.
Norway	At clinician's discretion for influenza virus and RSV<br><br>Mixed for SARS-CoV-2, varying from exhaustive testing (according to national guidelines) to selection at the clinician's discretion over time	Mixed	Up until April 2022, it was recommended that everyone with acute respiratory symptoms be tested for SARS-CoV-2, either by PCR or RAT, with RAT results requiring confirmation PCR until January 2022. Beyond this, testing was primarily based on clinical discretion. Hence, testing practices may have varied by age group and geographical area. Further details are provided elsewhere. [3]<br><br>For influenza viruses and RSV, only PCR test results were included in this study, while for SARS-CoV-2, a small number of RATs were included as well. The exact proportion of RATs is unavailable, but likely <1% of the samples were tested by RATs only.

Country	Selection of SARI cases for testing [1]	Testing approach [2]	Description
Spain	Mixed, depending on the region and the respiratory season.<br><br>Catalonia, Valencian Community, and Galicia.<br>- Season 2020-21: at the clinician's discretion.<br>- Season 2021-22: systematic in Catalonia, at the clinician's discretion in Galicia and Valencian Community.<br>- Season 2022-23: systematic.<br><br>Mixed in the Basque Country.<br>- Non-sentinel hospitals: at the clinician's discretion, with a rather exhaustive approach in epidemic periods.<br>- Sentinel hospital in season 2022-23: exhaustive.	Mixed, depending on the region and the respiratory season.<br><br>Catalonia, Valencian Community, and Galicia: parallel testing; except in season 2020-21, when only SARS-CoV-2 testing was performed.<br><br>Mixed in the Basque Country.<br>- Non-sentinel hospitals: parallel testing in epidemic periods and unspecified in non-epidemic periods.<br>- Sentinel hospital in season 2022-23: parallel testing.	Hospitals had designated weekdays in which all patients admitted with SARI were tested against the three pathogens: SARS-CoV-2, influenza virus, and RSV. These patients were tested at the regional reference laboratory using mainly RT-PCR. Additionally, clinicians could perform RATs as part of routine practice, and these were also recorded in the system.<br><br>While this protocol was fully implemented in the 2022-23 season, it was progressively implemented along the study period, leading to an in increase in systematic testing for the three viruses over time. Furthermore, regions joined sentinel surveillance for the three pathogens at different times, resulting in some heterogeneity in SARI case selection and testing approaches across regions and throughout the study period.<br><br>The designated testing days varied by region: Catalonia (Monday and Tuesday), Valencian Community and Galicia: every day of the week. In the Basque Country, testing practices varied by hospital and were influenced by the epidemiology of SARI rather than the weekday of admission. During the 2022-23 season, all SARI cases in sentinel hospitals were tested for all three pathogens, as described above. In non-sentinel hospitals and in earlier periods, testing was based on the clinician's judgment, with RT-PCR as the primary method upon admission. During epidemic periods, most SARI cases underwent RT-PCR testing for all three pathogens, while in non-epidemic periods, the proportion of cases tested may have decreased.<br><br>Among study participants tested for SARS-CoV-2, 95% received RT-PCR (ranging from 81% to 95% by region), while 5% received RAT. Among those tested for influenza virus, 98% underwent RT-PCR (ranging from 86% to 100% by region), and 2% received RAT. Information on testing methods for RSV is not available.<br><br>Only laboratory tests with an available sample date and valid results were included in the study for all regions.

[1] Selection of SARI cases for testing: a) Exhaustive: all patients meeting specific criteria (e.g., hospitalised, hospitalised with respiratory symptoms, hospitalised with SARI, etc.) are tested as part of the surveillance system; b) Systematic: laboratory tests are conducted following a structured, consistent, and repeatable approach; c) Clinician discretion: selection of cases based on the clinician's judgment and not defined by surveillance needs, d) mixed: several approaches are used for the selection of SARI cases, these may vary by hospital, region, or over time. [2] Testing approach: a) Parallel: testing is conducted simultaneously for all three pathogens (e.g., multiplex PCR is used to detect SARS-CoV-2, influenza, and RSV); b) Serial: testing is conducted sequentially, starting with one pathogen. If the result is negative, additional tests are performed for the remaining pathogens (e.g. samples are tested first for SARS-CoV-2, and if negative, subsequently tested for influenza and RSV); c) Selective: testing is performed for a specific pathogen, independent of whether the patient is tested for the other pathogens (e.g., some patients may only be tested for SARS-CoV-2, regardless of influenza or RSV testing); d) Unspecified: the testing approach is not clearly defined or reported; d) Mixed: several approaches are used for testing, these may vary by hospital, region, or over time. [3] Seppälä, E., Bøås, H., Dahl, J., Stålcrantz, J., Stecher, M., Tønnessen, R., et al. Registry-based surveillance of severe acute respiratory infections in Norway during 2021-2024. Influenza Other Respir Viruses [Internet]. 2025 Feb; Available from: https://onlinelibrary.wiley.com/doi/10.1111/irv.70080. Abbreviations; RAT: Rapid Antigen Test. RT-PCR: Reverse Transcription Reverse Transcription Polymerase Chain Reaction. RSV: Respiratory Syncytial Virus. SARI: Severe Acute Respiratory Infection.

Table S2. Periods of high and low SARI activity; Denmark, Iceland, Malta, Norway, Spain; week 21 of 2021 to week 39 of 2023.

Country	66th percentile of SARI admissions/100K pop.	Weeks of high SARI activity i.e., SARI admissions exceeding the 66th percentile, within the study period [1]	SARI case definition used to estimate weekly SARI admissions [2]
Denmark	35.5	2021-W36, 2021-W38, 2021-W39, 2021-W41 - 2022-W15, 2022-W04, 2022-W41, 2022-W43, 2022-W45, 2022-W48 - 2023-W01, 2023-W06, 2023-W11	A hospitalised patient with any of the following codes :<br>URIs: J00-J06<br>LRIs including Influenza and RSV infection: J09-J22<br>Country-specific COVID-19 codes : B34.2A, B97.2A, Z038PA1
Iceland	12.2	2021-W50- 2022-W02, 2022-W04 - W16, 2022-W23- W29, 2022-W42, 2022-W44 - 2023-W03, 2023-W11, 2023-W36	A hospitalised patient with any of the following codes :<br>LRIs including influenza virus and RSV: J09, J10, J11, J12, J13, J14, J15, J16, J17, J18, J20, J21, J22<br>COVID : U07.1, U07.2
Malta	8.3	2021-W50, 2021-W52, 2022-W14, 2022-W25, 2022-W26, 2022-W46, 2022-W50 - 2023-W02, 2023 - W06 - 2023-W13, 2023-W16, 2023-W22, 2023-W27, 2023-W29, 2023-W32	A hospitalised, acute respiratory patient with:<br>- Symptom onset 10 days or less prior to admission and<br>- Fever (38.0ºC <) or feverishness (37.0ºC – 37.9 ºC) and<br>- Cough or shortness of breath,<br>- Hospitalised for more than 24 hours
Norway	24.7	2021-W43 - W50, 2022-W07 - W16, 2022-W23 - W29, 2022-W43 - 2023-W05, 2023-W13	A hospitalised patient with any of the following codes:<br>URIs: J00-J06<br>LRIs including Influenza and RSV infection: J09-J22<br>ARDS: J80<br>COVID-19: U07.1, U07.2<br>Pertussis: A37<br>Diseases of middle ear and mastoid: H65-H67 (only the codes for acute or unspecified OM, excluding chronic OM
Spain	14.6	2021W29 - 31, 2021W45 - 2022W05, 2022W17 - 20, 2022W23 - 29, 2022W45 - 2023W10	A hospitalised, acute respiratory patient with symptom onset 10 days or less before admission with specific diagnostic impressions [3] or ICD-10 admission codes [4]

[1] Study period: from 2021-W21 to 2023-W39. [2] ICD-10 codes are defined in Annex 1. [3] Diagnostic impression included in Spain: Respiratory infection, Acute respiratory infection, Bronchiolitis, SARS infection, COVID infection, RSV infection, Flu infection; Dyspnea, Acute respiratory failure; Pneumonia, Right-sided pneumonia, Bilateral pneumonia, Community-acquired pneumonia, Pneumonia due to unspecified organism, Bronchopneumonia, Lung involvement, Pulmonary infiltrates, Pulmonary opacities; Asthma exacerbation, Exacerbated asthma, Asthmatic flare-up, Asthma crisis, Bronchial hyperreactivity, Bronchospasm; Exacerbated Chronic obstructive pulmonary disease (COPD), COPD with acute exacerbation. [4] ICD-10 codes included in the Spanish SARI case definition to determine activity: R06.0 – Dyspnea (shortness of breath), J80 – Acute Respiratory Distress Syndrome (ARDS), R06.89 – Other abnormalities of breathing, R06.9 – Unspecified abnormalities of breathing, J00-J06 – Acute upper respiratory infections, J09-J18 – Influenza and pneumonia, J20-J22 – Other acute lower respiratory infections, J40-J42 – Bronchitis, not specified as acute or chronic, J44.x – COPD, J45.x – Asthma, including status asthmaticus, J96.x – Respiratory failure, not classified elsewhere, J12.82 – COVID-19 pneumonia, U07.1 – COVID-19, virus identified. Abbreviations; ARDS: Acute Respiratory Distress Syndrome. LRIs: lower respiratory tract infections. OM: otitis media. RSV: Respiratory Syncytial Virus. SARS: Severe Acute Respiratory Syndrome. URIs: upper respiratory tract infections.

Table S3. Testing status and testing results for hospitalisations with respiratory disease and COVID-19 ICD-10 codes; stratified by SARI activity and age group; Denmark, Iceland, Malta, Norway, Spain; week 21 of 2021 to week 39 of 2023.

Strata		Hospitalisations meeting inclusion criteria [1]	SARS-CoV-2				Influenza virus				RSV				SARS-CoV-2, influenza virus, RSV			
			Tested		(+)		Tested		(+)		Tested		(+)		Tested for the three		(+) for at least one	
			N. tested	Prop. tested (%)	N. testing positive	Test positivity (%)	N. tested	Prop. tested (%)	N. testing positive	Test positivity (%)	N. tested	Prop. tested (%)	N. testing positive	Test positivity (%)	N. tested	Prop. tested (%)	N. testing positive	Test positivity (%)
Denmark	All ages	237,208	176,319	74.3	32,832	18.6	134,842	56.8	6,522	4.8	71,563	30.2	6,680	9.3	68,594	28.9	19,237	28.0
	High SARI[2]	98,047	82,873	84.5	19,819	23.9	60,344	61.5	4,060	6.7	32,443	33.1	3,779	11.6	30,821	31.4	10,531	34.2
	Low SARI[3]	139,161	93,446	67.1	13,013	13.9	74,498	53.5	2,462	3.3	39,120	28.1	2,901	7.4	37,773	27.1	8,706	23.0
	0-4	19,588	13,458	68.7	1,243	9.2	12,890	65.8	455	3.5	12,665	64.7	4,267	33.7	10,988	56.1	4,429	40.3
	5-14 y.o	4,684	2,546	54.4	243	9.5	2,028	43.3	331	16.3	1,626	34.7	67	4.1	1,507	32.2	370	24.6
	15-64 y.o.	64,860	44,135	68.0	9,243	20.9	30,393	46.9	2,123	7.0	14,670	22.6	536	3.7	14,322	22.1	3,905	27.3
	65+ y.o.	148,076	116,180	78.5	22,103	19.0	89,531	60.5	3,613	4.0	42,602	28.8	1,810	4.2	41,777	28.2	10,533	25.2
Iceland	All ages	7,052	4,268	60.5	1,632	38.2	1,834	26.0	179	9.8	*NA*	*NA*	*NA*	*NA*	*NA*	*NA*	*NA*	*NA*
	High SARI[2]	3,419	2,324	68.0	1,046	45.0	868	25.4	135	15.6	*NA*	*NA*	*NA*	*NA*	*NA*	*NA*	*NA*	*NA*
	Low SARI[3]	3,633	1,944	53.5	586	30.1	966	26.6	44	4.6	*NA*	*NA*	*NA*	*NA*	*NA*	*NA*	*NA*	*NA*
	0- 4 y.o	823	556	67.6	68	12.2	491	59.7	21	4.3	*NA*	*NA*	*NA*	*NA*	*NA*	*NA*	*NA*	*NA*
	5-14 y.o	131	80	61.1	15	18.8	66	50.4	7	10.6	*NA*	*NA*	*NA*	*NA*	*NA*	*NA*	*NA*	*NA*
	15-64 y.o.	1,962	1,169	59.6	548	46.9	373	19.0	47	12.6	*NA*	*NA*	*NA*	*NA*	*NA*	*NA*	*NA*	*NA*
	65+ y.o.	4,136	2,463	59.6	1,001	40.6	904	21.9	104	11.5	*NA*	*NA*	*NA*	*NA*	*NA*	*NA*	*NA*	*NA*
Malta	All ages	14,066	9,503	67.6	2,226	23.4	6,950	49.4	403	5.8	6,950	49.4	321	4.62	4,486	31.9	711	15.9
	High SARI[2]	3,280	1,468	44.8	432	29.4	1,978	60.3	124	6.3	1,978	60.3	75	3.79	877	26.7	156	17.8
	Low SARI[3]	10,786	8,035	74.5	1,794	22.3	4,972	46.1	279	5.6	4,972	46.1	246	4.95	3,609	33.5	555	15.4
	0- 4 y.o	1,138	646	56.8	131	20.3	582	51.1	26	4.5	582	51.1	146	25.1	269	23.6	101	37.5
	5-14 y.o	252	120	47.6	22	18.3	128	50.8	19	14.8	128	50.8	4	3.12	51	20.2	8	1.1
	15-64 y.o.	3,596	2,561	71.2	680	26.6	1,668	46.4	117	7.0	1,668	46.4	39	2.34	1,182	32.9	190	26.7
	65+ y.o.	9,080	6,176	68.0	1,393	22.6	4,572	50.4	241	5.3	4,572	50.4	132	2.89	2,984	32.9	412	58.0
Norway	All ages	271,097	176,593	65.1	37,918	21.5	159,354	58.8	8,908	5.6	112,802	41.6	8,453	7.5	111,687	41.2	34,301	30.7
	High SARI[2]	107,652	77,056	71.6	21,726	28.2	71,148	66.1	6,180	8.7	50,539	46.9	6,331	12.5	50,096	46.5	21,694	43.3
	Low SARI[3]	163,445	99,537	60.9	16,192	16.3	88,206	54.0	2,728	3.1	62,263	38.1	2,122	3.4	61,591	37.7	12,607	20.5
	0-4 y.o	21,227	16,645	78.4	1,348	8.1	16,208	76.4	735	4.5	15,257	71.9	4,586	30.1	15,094	71.1	6,203	41.1
	5-14 y.o	6,073	3,515	57.9	406	11.6	3,247	53.5	587	18.1	2,918	48	200	6.9	2,891	47.6	947	32.8

	15-64 y.o.	72,579	44,905	61.9	11,089	24.7	38,475	53.0	3,086	8.0	26,894	37.1	1,019	3.8	26,625	36.7	8,108	30.5
	65+ y.o.	171,218	111,528	65.1	25,075	22.5	101,424	59.2	4,500	4.4	67,733	39.6	2,648	3.9	67,077	39.2	19,043	28.4
**Spain**	All ages	35,161	28,480	81.0	6,435	22.6	10,438	29.7	1,677	16.1	1,621	4.6	280	17.3	1,584	4.5	649	41.0
	High SARI[2]	16,617	13,865	83.4	3,567	25.7	4,618	27.8	602	13.0	966	5.8	232	24.0	948	5.7	468	49.4
	Low SARI[3]	18,544	14,615	78.8	2,868	19.6	5,820	31.4	1,075	18.5	655	3.5	48	7.3	636	3.4	181	28.5
	0-4 y.o.	2,965	2,153	72.6	125	5.8	1,076	36.3	67	6.2	631	21.3	234	37.1	616	20.8	259	42.0
	5-14 y.o	812	626	77.1	24	3.8	346	42.6	44	12.7	73	9.0	4	5.5	71	8.7	14	19.7
	15-64 y.o.	8,045	6,169	76.7	1,247	20.2	2,158	26.8	423	19.6	214	2.7	10	4.7	209	2.6	82	39.2
	65+ y.o.	23,339	19,532	83.7	5,039	25.8	6,858	29.4	1,143	16.7	703	3.0	32	4.6	688	2.9	294	42.7

[1] Hospitalisations with respiratory disease ICD-10 codes: J00-J99 or COVID-19 ICD-10 codes: U07.1, U07.2, and Danish codes B34.2A, B97.2A, Z03.8PA1. [2] High SARI: high SARI activity period, defined by weeks where the number of SARI admissions exceeded the 66th percentile of the number of SARI admissions/100,000 population within the study period. [3] Low SARI: low SARI activity period, defined by weeks where the number of SARI admissions were below the 66th percentile threshold. Abbreviations; COVID-19: Coronavirus Disease 2019. N: Number. Prop: Proportion. NA: not available. RSV: Respiratory Syncytial Virus. ICD-10: 10th edition of the International Classification of Disease codes. SARI: severe acute respiratory infection. SARS-CoV-2: Severe Acute Respiratory Syndrome Coronavirus 2. y.o.: years old.

Table S4. Accuracy of the ICD-10 code-based case definitions for SARI associated with SARS-CoV-2, influenza virus, RSV or any of the three viruses; Denmark, Iceland, Malta, Norway, Spain; week 21 of 2021 to week 39 of 2023.

DENMARK

SARI-SARS-CoV-2		ICD 1	ICD 2	ICD 3	ICD 4	ICD 5	ICD 6	ICD 7	ICD 8	ICD 9	ICD 10	TP	FP	FN	TN	Se (%)	PPV (%)	CUI	
All ages	1	B34										27,206	1,373	5,626	142,114	82.9	95.2	0.789	G
	2	B34	B97									29,606	1,421	3,226	142,066	90.2	95.4	0.860	E
	3	B34	B97	Z03								30,031	16,632	2,801	126,855	91.5	64.4	0.589	S
	4	B34	B97	Z03	J18							31,092	63,608	1,740	79,879	94.7	32.8	0.311	P
	5	B34	B97	Z03	J18	J15						31,356	74,186	1,476	69,301	95.5	29.7	0.284	P
	6	B34	B97	Z03	J18	J15	J05					31,402	74,792	1,430	68,695	95.6	29.6	0.283	P
	7	B34	B97	Z03	J18	J15	J05	J96				31,626	84,989	1,206	58,498	96.3	27.1	0.261	P
	8	B34	B97	Z03	J18	J15	J05	J96	J84			31,662	86,101	1,170	57,386	96.4	26.9	0.259	P
	9	B34	B97	Z03	J18	J15	J05	J96	J84	J80		31,663	86,130	1,169	57,357	96.4	26.9	0.259	P
	10	B34	B97	Z03	J18	J15	J05	J96	J84	J80	J44	32,127	109,939	705	33,548	97.9	22.6	0.221	P
High SARI	1	B34										16,710	860	3,109	62,194	84.3	95.1	0.802	G
	2	B34	B97									18,087	891	1,732	62,163	91.3	95.3	0.870	E
	3	B34	B97	Z03								18,367	9,722	1,452	53,332	92.7	65.4	0.606	S
	4	B34	B97	Z03	J96							18,521	15,356	1,298	47,698	93.5	54.7	0.511	S
	5	B34	B97	Z03	J96	J05						18,549	15,585	1,270	47,469	93.6	54.3	0.509	S
	6	B34	B97	Z03	J96	J05	J15					18,702	20,294	1,117	42,760	94.4	48.0	0.453	P
	7	B34	B97	Z03	J96	J05	J15	J18				19,139	37,399	680	25,655	96.6	33.9	0.327	P
	8	B34	B97	Z03	J96	J05	J15	J18	J84			19,161	37,867	658	25,187	96.7	33.6	0.325	P
	9	B34	B97	Z03	J96	J05	J15	J18	J84	J69		19,180	38,371	639	24,683	96.8	33.3	0.323	P
	10	B34	B97	Z03	J96	J05	J15	J18	J84	J69	J44	19,414	48,308	405	14,746	98.0	28.7	0.281	P
Low SARI	1	B34										10,496	513	2,517	79,920	80.7	95.3	0.769	G
	2	B34	B97									11,519	530	1,494	79,903	88.5	95.6	0.846	E
	3	B34	B97	Z03								11,664	6,910	1,349	73,523	89.6	62.8	0.563	S
	4	B34	B97	Z03	J15							11,807	14,350	1,206	66,083	90.7	45.1	0.410	P
	5	B34	B97	Z03	J15	J18						12,370	41,349	643	39,084	95.1	23.0	0.219	P
	6	B34	B97	Z03	J15	J18	J14					12,379	41,596	634	38,837	95.1	22.9	0.218	P
	7	B34	B97	Z03	J15	J18	J14	J05				12,397	41,968	616	38,465	95.3	22.8	0.217	P
	8	B34	B97	Z03	J15	J18	J14	J05	J80			12,398	42,018	615	38,415	95.3	22.8	0.217	P
	9	B34	B97	Z03	J15	J18	J14	J05	J80	J96		12,496	47,839	517	32,594	96.0	20.7	0.199	P
	10	B34	B97	Z03	J15	J18	J14	J05	J80	J96	J84	12,510	48,483	503	31,950	96.1	20.5	0.197	P
0-4	1	B34										1,031	23	212	12,192	82.9	97.8	0.811	E
	2	B34	J05									1,074	490	169	11,725	86.4	68.7	0.593	S
	3	B34	J05	B97								1,095	492	148	11,723	88.1	69.0	0.608	S
	4	B34	J05	B97	Z03							1,119	1,468	124	10,747	90.0	43.3	0.389	P
	5	B34	J05	B97	Z03	J06						1,169	3,852	74	8,363	94.0	23.3	0.219	P
	6	B34	J05	B97	Z03	J06	J98					1,173	4,020	70	8,195	94.4	22.6	0.213	P
	7	B34	J05	B97	Z03	J06	J98	J04				1,176	4,064	67	8,151	94.6	22.4	0.212	P
	8	B34	J05	B97	Z03	J06	J98	J04	J45			1,190	5,246	53	6,969	95.7	18.5	0.177	P
	9	B34	J05	B97	Z03	J06	J98	J04	J45	J96		1,199	6,333	44	5,882	96.5	15.9	0.154	P
	10	B34	J05	B97	Z03	J06	J98	J04	J45	J96	J15	1,203	6,571	40	5,644	96.8	15.5	0.150	P
5-14	1	B34										205	17	38	2,286	84.4	92.3	0.779	G
	2	B34	B97									210	17	33	2,286	86.4	92.5	0.799	G
	3	B34	B97	J96								213	237	30	2,066	87.7	47.3	0.415	P

	4	B34	B97	J96	J05							216	326	27	1,977	88.9	39.9	0.354	P
	5	B34	B97	J96	J05	Z03						218	463	25	1,840	89.7	32.0	0.287	P
	6	B34	B97	J96	J05	Z03	J18					222	684	21	1,619	91.4	24.5	0.224	P
	7	B34	B97	J96	J05	Z03	J18	J09				226	814	17	1,489	93.0	21.7	0.202	P
	8	B34	B97	J96	J05	Z03	J18	J09	J12			226	833	17	1,470	93.0	21.3	0.198	P
	9	B34	B97	J96	J05	Z03	J18	J09	J12	J15		226	889	17	1,414	93.0	20.3	0.189	P
	10	B34	B97	J96	J05	Z03	J18	J09	J12	J15	J45	231	1,592	12	711	95.1	12.7	0.120	P
**15-64**	1	B34										7,901	1,003	1,342	33,889	85.5	88.7	0.759	G
	2	B34	B97									8,537	1,025	706	33,867	92.4	89.3	0.825	E
	3	B34	B97	Z03								8,668	5,215	575	29,677	93.8	62.4	0.586	S
	4	B34	B97	Z03	J96							8,754	8,910	489	25,982	94.7	49.6	0.469	P
	5	B34	B97	Z03	J96	J69						8,765	9,253	478	25,639	94.8	48.6	0.461	P
	6	B34	B97	Z03	J96	J69	J84					8,771	9,519	472	25,373	94.9	48.0	0.455	P
	7	B34	B97	Z03	J96	J69	J84	J06				8,782	9,919	461	24,973	95.0	47.0	0.446	P
	8	B34	B97	Z03	J96	J69	J84	J06	J80			8,782	9,941	461	24,951	95.0	46.9	0.446	P
	9	B34	B97	Z03	J96	J69	J84	J06	J80	J45		8,847	14,349	396	20,543	95.7	38.1	0.365	P
	10	B34	B97	Z03	J96	J69	J84	J06	J80	J45	J15	8,894	16,454	349	18,438	96.2	35.1	0.338	P
**65+**	1	B34										18,069	330	4,034	93,747	81.7	98.2	0.803	G
	2	B34	B97									19,806	353	2,297	93,724	89.6	98.2	0.880	E
	3	B34	B97	Z03								20,072	10,229	2,031	83,848	90.8	66.2	0.602	S
	4	B34	B97	Z03	J06							20,092	10,662	2,011	83,415	90.9	65.3	0.594	S
	5	B34	B97	Z03	J06	J15						20,333	20,263	1,770	73,814	92.0	50.1	0.461	P
	6	B34	B97	Z03	J06	J15	J18					21,191	55,062	912	39,015	95.9	27.8	0.266	P
	7	B34	B97	Z03	J06	J15	J18	J96				21,334	61,095	769	32,982	96.5	25.9	0.250	P
	8	B34	B97	Z03	J06	J15	J18	J96	J80			21,335	61,109	768	32,968	96.5	25.9	0.250	P
	9	B34	B97	Z03	J06	J15	J18	J96	J80	J06		21,391	63,460	712	30,617	96.8	25.2	0.244	P
	10	B34	B97	Z03	J06	J15	J18	J96	J80	J06	J16	21,395	63,497	708	30,580	96.8	25.2	0.244	P

DENMARK

SARI-Influenza		ICD 1	ICD 2	ICD 3	ICD 4	ICD 5	ICD 6	ICD 7	ICD 8	ICD 9	ICD 10	TP	FP	FN	TN	Se (%)	PPV(%)	CUI	
**All ages**	1	J09										4,807	66	1,715	128,254	73.7	98.6	0.727	G
	2	J09	J10									5,469	82	1,053	128,238	83.9	98.5	0.826	E
	3	J09	J10	J11								5,508	104	1,014	128,216	84.5	98.1	0.829	E
	4	J09	J10	J11	J45							5,642	8,298	880	120,022	86.5	40.5	0.350	P
	5	J09	J10	J11	J45	J46						5,652	8,790	870	119,530	86.7	39.1	0.339	P
	6	J09	J10	J11	J45	J46	Z03					5,730	20,835	792	107,485	87.9	21.6	0.190	P
	7	J09	J10	J11	J45	J46	Z03	J13				5,738	21,352	784	106,968	88.0	21.2	0.186	P
	8	J09	J10	J11	J45	J46	Z03	J13	J44			5,907	48,603	615	79,717	90.6	10.8	0.098	P
	9	J09	J10	J11	J45	J46	Z03	J13	J44	J18		6,208	82,533	314	45,787	95.2	7.0	0.067	P
	10	J09	J10	J11	J45	J46	Z03	J13	J44	J18	J15	6,264	90,097	258	38,223	96.0	6.5	0.062	P
**High SARI**	1	J09										3,199	47	861	56,237	78.8	98.6	0.777	G
	2	J09	J10									3,454	52	606	56,232	85.1	98.5	0.838	E
	3	J09	J10	J44								3,575	12,549	485	43,735	88.1	22.2	0.195	P
	4	J09	J10	J44	J45							3,654	15,531	406	40,753	90.0	19.0	0.171	P
	5	J09	J10	J44	J45	J11						3,673	15,544	387	40,740	90.5	19.1	0.173	P
	6	J09	J10	J44	J45	J11	J46					3,677	15,705	383	40,579	90.6	19.0	0.172	P
	7	J09	J10	J44	J45	J11	J46	J18				3,830	30,360	230	25,924	94.3	11.2	0.106	P
	8	J09	J10	J44	J45	J11	J46	J18	Z03			3,871	34,949	189	21,335	95.3	10.0	0.095	P
	9	J09	J10	J44	J45	J11	J46	J18	Z03	J06		3,908	36,182	152	20,102	96.3	9.7	0.094	P
	10	J09	J10	J44	J45	J11	J46	J18	Z03	J06	J14	3,909	36,335	151	19,949	96.3	9.7	0.094	P
**Low SARI**	1	J09										1,608	19	854	72,017	65.3	98.8	0.646	G
	2	J09	J10									2,015	30	447	72,006	81.8	98.5	0.806	G
	3	J09	J10	J11								2,035	39	427	71,997	82.7	98.1	0.811	E
	4	J09	J10	J11	J45							2,085	4,954	377	67,082	84.7	29.6	0.251	P
	5	J09	J10	J11	J45	J13						2,091	5,271	371	66,765	84.9	28.4	0.241	P
	6	J09	J10	J11	J45	J13	J14					2,098	5,662	364	66,374	85.2	27.0	0.230	P
	7	J09	J10	J11	J45	J13	J14	J15				2,136	12,211	326	59,825	86.8	14.9	0.129	P
	8	J09	J10	J11	J45	J13	J14	J15	J06			2,167	14,203	295	57,833	88.0	13.2	0.117	P
	9	J09	J10	J11	J45	J13	J14	J15	J06	J18		2,331	37,632	131	34,404	94.7	5.8	0.055	P
	10	J09	J10	J11	J45	J13	J14	J15	J06	J18	J46	2,337	37,910	125	34,126	94.9	5.8	0.055	P
**0-4**	1	J09										238	14	217	12,421	52.3	94.4	0.494	S
	2	J09	J10									339	17	116	12,418	74.5	95.2	0.709	G

	3	J09	J10	J18								367	1,432	88	11,003	80.7	20.4	0.165	P
	4	J09	J10	J18	J11							371	1,433	84	11,002	81.5	20.6	0.168	P
	5	J09	J10	J18	J11	J96						381	2,681	74	9,754	83.7	12.4	0.104	P
	6	J09	J10	J18	J11	J96	J05					388	3,139	67	9,296	85.3	11.0	0.094	P
	7	J09	J10	J18	J11	J96	J05	J03				391	3,396	64	9,039	85.9	10.3	0.089	P
	8	J09	J10	J18	J11	J96	J05	J03	J06			424	5,736	31	6,699	93.2	6.9	0.064	P
	9	J09	J10	J18	J11	J96	J05	J03	J06	J45		432	6,717	23	5,718	94.9	6.0	0.057	P
	10	J09	J10	J18	J11	J96	J05	J03	J06	J45	J112	434	7,264	21	5,171	95.4	5.6	0.054	P
**5-14**	1	J09										152	12	179	1,685	45.9	92.7	0.426	P
	2	J09	J10									260	13	71	1,684	78.5	95.2	0.748	G
	3	J09	J10	J04								265	22	66	1,675	80.1	92.3	0.739	G
	4	J09	J10	J04	J11							269	24	62	1,673	81.3	91.8	0.746	G
	5	J09	J10	J04	J11	J06						277	84	54	1,613	83.7	76.7	0.642	G
	6	J09	J10	J04	J11	J06	J05					289	220	42	1,477	87.3	56.8	0.496	S
	7	J09	J10	J04	J11	J06	J05	J18				299	450	32	1,247	90.3	39.9	0.361	P
	8	J09	J10	J04	J11	J06	J05	J18	J00			300	453	31	1,244	90.6	39.8	0.361	P
	9	J09	J10	J04	J11	J06	J05	J18	J00	Z03		302	525	29	1,172	91.2	36.5	0.333	P
	10	J09	J10	J04	J11	J06	J05	J18	J00	Z03	J69	304	530	27	1,167	91.8	36.5	0.335	P
**15-64**	1	J09										1,451	16	672	28,254	68.3	98.9	0.676	G
	2	J09	J10									1,749	19	374	28,251	82.4	98.9	0.815	E
	3	J09	J10	J45								1,828	3,668	295	24,602	86.1	33.3	0.286	P
	4	J09	J10	J45	J11							1,846	3,682	277	24,588	87.0	33.4	0.290	P
	5	J09	J10	J45	J11	J06						1,870	4,068	253	24,202	88.1	31.5	0.277	P
	6	J09	J10	J45	J11	J06	J46					1,879	4,321	244	23,949	88.5	30.3	0.268	P
	7	J09	J10	J45	J11	J06	J46	J18				1,991	11,976	132	16,294	93.8	14.3	0.134	P
	8	J09	J10	J45	J11	J06	J46	J18	J15			2,010	13,704	113	14,566	94.7	12.8	0.121	P
	9	J09	J10	J45	J11	J06	J46	J18	J15	J13		2,011	13,802	112	14,468	94.7	12.7	0.120	P
	10	J09	J10	J45	J11	J06	J46	J18	J15	J13	Z03	2,030	16,006	93	12,264	95.6	11.3	0.108	P
**65+**	1	J09										2,966	24	647	85,894	82.1	99.2	0.814	E
	2	J09	J10									3,121	33	492	85,885	86.4	99.0	0.855	E
	3	J09	J10	J06								3,138	671	475	85,247	86.9	82.4	0.716	G
	4	J09	J10	J06	J44							3,286	25,558	327	60,360	90.9	11.4	0.104	P
	5	J09	J10	J06	J44	Z03						3,311	31,984	302	53,934	91.6	9.4	0.086	P
	6	J09	J10	J06	J44	Z03	J13					3,316	32,260	297	53,658	91.8	9.3	0.086	P
	7	J09	J10	J06	J44	Z03	J13	J11				3,326	32,264	287	53,654	92.1	9.3	0.086	P
	8	J09	J10	J06	J44	Z03	J13	J11	J45			3,344	34,315	269	51,603	92.6	8.9	0.082	P
	9	J09	J10	J06	J44	Z03	J13	J11	J45	J14		3,351	34,689	262	51,229	92.7	8.8	0.082	P
	10	J09	J10	J06	J44	Z03	J13	J11	J45	J14	J15	3,391	41,361	222	44,557	93.9	7.6	0.071	P

DENMARK

SARI-RSV		ICD 1	ICD 2	ICD 3	ICD 4	ICD 5	ICD 6	ICD 7	ICD 8	ICD 9	ICD 10	TP	FP	FN	TN	Se (%)	PPV(%)	CUI	
**All ages**	1	J21										1,960	338	4,720	64,545	29.3	85.3	0.250	P
	2	J21	J20									3,000	2,279	3,680	62,604	44.9	56.8	0.255	P
	3	J21	J20	J12								3,935	2,870	2,745	62,013	58.9	57.8	0.341	P
	4	J21	J20	J12	J06							4,370	5,269	2,310	59,614	65.4	45.3	0.297	P
	5	J21	J20	J12	J06	J96						4,718	10,705	1,962	54,178	70.6	30.6	0.216	P
	6	J21	J20	J12	J06	J96	J00					4,736	10,823	1,944	54,060	70.9	30.4	0.216	P
	7	J21	J20	J12	J06	J96	J00	J98				4,760	11,113	1,920	53,770	71.3	30.0	0.214	P
	8	J21	J20	J12	J06	J96	J00	J98	J45			5,019	15,014	1,661	49,869	75.1	25.1	0.188	P
	9	J21	J20	J12	J06	J96	J00	J98	J45	J13		5,036	15,260	1,644	49,623	75.4	24.8	0.187	P
	10	J21	J20	J12	J06	J96	J00	J98	J45	J13	J39	5,043	15,371	1,637	49,512	75.5	24.7	0.186	P
**High SARI**	1	J21										1,012	208	2,767	28,456	26.8	83.0	0.222	P
	2	J21	J20									1,578	1,053	2,201	27,611	41.8	60.0	0.250	P
	3	J21	J20	J12								2,097	1,340	1,682	27,324	55.5	61.0	0.339	P
	4	J21	J20	J12	J06							2,312	2,371	1,467	26,293	61.2	49.4	0.302	P
	5	J21	J20	J12	J06	J96						2,521	4,645	1,258	24,019	66.7	35.2	0.235	P
	6	J21	J20	J12	J06	J96	J00					2,531	4,704	1,248	23,960	67.0	35.0	0.234	P
	7	J21	J20	J12	J06	J96	J00	J98				2,547	4,817	1,232	23,847	67.4	34.6	0.233	P
	8	J21	J20	J12	J06	J96	J00	J98	J18			3,055	12,405	724	16,259	80.8	19.8	0.160	P
	9	J21	J20	J12	J06	J96	J00	J98	J18	J45		3,186	13,783	593	14,881	84.3	18.8	0.158	P
	10	J21	J20	J12	J06	J96	J00	J98	J18	J45	J13	3,195	13,860	584	14,804	84.5	18.7	0.158	P

Low SARI	1	J21										948	130	1,953	36,089	32.7	87.9	0.287	P
	2	J21	J20									1,422	1,226	1,479	34,993	49.0	53.7	0.263	P
	3	J21	J20	J12								1,838	1,530	1,063	34,689	63.4	54.6	0.346	P
	4	J21	J20	J12	J06							2,058	2,898	843	33,321	70.9	41.5	0.295	P
	5	J21	J20	J12	J06	J96						2,197	6,060	704	30,159	75.7	26.6	0.202	P
	6	J21	J20	J12	J06	J96	J00					2,205	6,119	696	30,100	76.0	26.5	0.201	P
	7	J21	J20	J12	J06	J96	J00	J40				2,214	6,195	687	30,024	76.3	26.3	0.201	P
	8	J21	J20	J12	J06	J96	J00	J40	J98			2,222	6,372	679	29,847	76.6	25.9	0.198	P
	9	J21	J20	J12	J06	J96	J00	J40	J98	J45		2,329	8,650	572	27,569	80.3	21.2	0.170	P
	10	J21	J20	J12	J06	J96	J00	J40	J98	J45	Z03	2,415	11,475	486	24,744	83.2	17.4	0.145	P
0-4	1	J21										1,914	315	2,353	8,083	44.9	85.9	0.385	P
	2	J21	J12									2,412	638	1,855	7,760	56.5	79.1	0.447	P
	3	J21	J12	J96								2,680	1,276	1,587	7,122	62.8	67.7	0.425	P
	4	J21	J12	J96	J20							3,469	2,789	798	5,609	81.3	55.4	0.451	P
	5	J21	J12	J96	J20	Z03						3,499	3,252	768	5,146	82.0	51.8	0.425	P
	6	J21	J12	J96	J20	Z03	J15					3,535	3,427	732	4,971	82.8	50.8	0.421	P
	7	J21	J12	J96	J20	Z03	J15	J06				3,906	5,109	361	3,289	91.5	43.3	0.397	P
	8	J21	J12	J96	J20	Z03	J15	J06	J13			3,911	5,114	356	3,284	91.7	43.3	0.397	P
	9	J21	J12	J96	J20	Z03	J15	J06	J13	J98		3,930	5,194	337	3,204	92.1	43.1	0.397	P
	10	J21	J12	J96	J20	Z03	J15	J06	J13	J98	J18	4,067	5,930	200	2,468	95.3	40.7	0.388	P
5-14	1	J21										8	4	59	1,555	11.9	66.7	0.080	P
	2	J21	J96									20	134	47	1,425	29.9	13.0	0.039	P
	3	J21	J96	J12								25	156	42	1,403	37.3	13.8	0.052	P
	4	J21	J96	J12	J45							49	652	18	907	73.1	7.0	0.051	P
	5	J21	J96	J12	J45	J18						53	819	14	740	79.1	6.1	0.048	P
	6	J21	J96	J12	J45	J18	J15					57	858	10	701	85.1	6.2	0.053	P
	7	J21	J96	J12	J45	J18	J15	J20				59	890	8	669	88.1	6.2	0.055	P
	8	J21	J96	J12	J45	J18	J15	J20	J98			59	901	8	658	88.1	6.1	0.054	P
	9	J21	J96	J12	J45	J18	J15	J20	J98	J34		60	902	7	657	89.6	6.2	0.056	P
	10	J21	J96	J12	J45	J18	J15	J20	J98	J34	J06	62	998	5	561	92.5	5.8	0.054	P
15-64	1	J12										107	90	429	14,044	20.0	54.3	0.108	P
	2	J12	J21									124	100	412	14,034	23.1	55.4	0.128	P
	3	J12	J21	J45								214	1,828	322	12,306	39.9	10.5	0.042	P
	4	J12	J21	J45	J20							239	1,897	297	12,237	44.6	11.2	0.050	P
	5	J12	J21	J45	J20	J18						371	5,735	165	8,399	69.2	6.1	0.042	P
	6	J12	J21	J45	J20	J18	J44					432	7,361	104	6,773	80.6	5.5	0.045	P
	7	J12	J21	J45	J20	J18	J44	J13				435	7,410	101	6,724	81.2	5.5	0.045	P
	8	J12	J21	J45	J20	J18	J44	J13	J14			438	7,473	98	6,661	81.7	5.5	0.045	P
	9	J12	J21	J45	J20	J18	J44	J13	J14	J06		446	7,670	90	6,464	83.2	5.5	0.046	P
	10	J12	J21	J45	J20	J18	J44	J13	J14	J06	J15	471	8,572	65	5,562	87.9	5.2	0.046	P
65+	1	J12										374	195	1,436	40,597	20.7	65.7	0.136	P
	2	J12	J20									470	333	1,340	40,459	26.0	58.5	0.152	P
	3	J12	J20	J21								490	342	1,320	40,450	27.1	58.9	0.159	P
	4	J12	J20	J21	J44							981	11,720	829	29,072	54.2	7.7	0.042	P
	5	J12	J20	J21	J44	J45						1,037	12,655	773	28,137	57.3	7.6	0.043	P
	6	J12	J20	J21	J44	J45	J06					1,056	12,848	754	27,944	58.3	7.6	0.044	P
	7	J12	J20	J21	J44	J45	J06	J18				1,487	25,393	323	15,399	82.2	5.5	0.045	P
	8	J12	J20	J21	J44	J45	J06	J18	Z03			1,590	28,770	220	12,022	87.8	5.2	0.046	P
	9	J12	J20	J21	J44	J45	J06	J18	Z03	J96		1,641	30,026	169	10,766	90.7	5.2	0.047	P
	10	J12	J20	J21	J44	J45	J06	J18	Z03	J96	J13	1,646	30,112	164	10,680	90.9	5.2	0.047	P

DENMARK

SARI- any of the three		ICD 1	ICD 2	ICD 3	ICD 4	ICD 5	ICD 6	ICD 7	ICD 8	ICD 9	ICD 10	TP	FP	FN	TN	Se (%)	PPV(%)	CUI	
All ages	1	B34										8,688	178	10,549	49,179	45.2	98.0	0.443	P
	2	B34	J09									11,040	223	8,197	49,134	57.4	98.0	0.563	S
	3	B34	J09	J21								12,534	516	6,703	48,841	65.2	96.0	0.626	S
	4	B34	J09	J21	B97							13,228	529	6,009	48,828	68.8	96.2	0.661	G
	5	B34	J09	J21	B97	J20						14,105	2,291	5,132	47,066	73.3	86.0	0.631	S
	6	B34	J09	J21	B97	J20	J12					14,953	2,800	4,284	46,557	77.7	84.2	0.655	G

	7	B34	J09	J21	B97	J20	J12	J10				15,296	2,809	3,941	46,548	79.5	84.5	0.672	G
	8	B34	J09	J21	B97	J20	J12	J10	J96			15,764	7,519	3,473	41,838	81.9	67.7	0.555	S
	9	B34	J09	J21	B97	J20	J12	J10	J96	Z03		16,250	14,316	2,987	35,041	84.5	53.2	0.449	P
	10	B34	J09	J21	B97	J20	J12	J10	J96	Z03	J06	16,644	16,115	2,593	33,242	86.5	50.8	0.440	P
**High SARI**	1	B34										4,691	99	5,840	20,191	44.5	97.9	0.436	P
	2	B34	J09									6,162	132	4,369	20,158	58.5	97.9	0.573	S
	3	B34	J09	B97								6,561	141	3,970	20,149	62.3	97.9	0.610	S
	4	B34	J09	B97	J21							7,306	319	3,225	19,971	69.4	95.8	0.665	G
	5	B34	J09	B97	J21	J20						7,781	1,065	2,750	19,225	73.9	88.0	0.650	G
	6	B34	J09	B97	J21	J20	J12					8,246	1,310	2,285	18,980	78.3	86.3	0.676	G
	7	B34	J09	B97	J21	J20	J12	J10				8,359	1,314	2,172	18,976	79.4	86.4	0.686	G
	8	B34	J09	B97	J21	J20	J12	J10	J96			8,622	3,186	1,909	17,104	81.9	73.0	0.598	S
	9	B34	J09	B97	J21	J20	J12	J10	J96	J06		8,821	3,974	1,710	16,316	83.8	68.9	0.577	S
	10	B34	J09	B97	J21	J20	J12	J10	J96	J06	J13	8,837	4,048	1,694	16,242	83.9	68.6	0.576	S
**Low SARI**	1	B34										3,997	79	4,709	28,988	45.9	98.1	0.450	P
	2	B34	J09									4,878	91	3,828	28,976	56.0	98.2	0.550	S
	3	B34	J09	J21								5,626	206	3,080	28,861	64.6	96.5	0.623	S
	4	B34	J09	J21	B97							5,922	210	2,784	28,857	68.0	96.6	0.657	G
	5	B34	J09	J21	B97	J12						6,329	493	2,377	28,574	72.7	92.8	0.674	G
	6	B34	J09	J21	B97	J12	J20					6,707	1,490	1,999	27,577	77.0	81.8	0.630	S
	7	B34	J09	J21	B97	J12	J20	J10				6,937	1,495	1,769	27,572	79.7	82.3	0.656	G
	8	B34	J09	J21	B97	J12	J20	J10	J96			7,142	4,333	1,564	24,734	82.0	62.2	0.511	S
	9	B34	J09	J21	B97	J12	J20	J10	J96	J06		7,348	5,454	1,358	23,613	84.4	57.4	0.484	P
	10	B34	J09	J21	B97	J12	J20	J10	J96	J06	Z03	7,522	8,042	1,184	21,025	86.4	48.3	0.418	P
**0-4**	1	B34										631	11	3,798	6,548	14.2	98.3	0.140	P
	2	B34	J21									2,084	283	2,345	6,276	47.1	88.0	0.414	P
	3	B34	J21	J09								2,264	296	2,165	6,263	51.1	88.4	0.452	P
	4	B34	J21	J09	J12							2,697	571	1,732	5,988	60.9	82.5	0.503	S
	5	B34	J21	J09	J12	J10						2,766	574	1,663	5,985	62.5	82.8	0.517	S
	6	B34	J21	J09	J12	J10	J96					2,987	1,113	1,442	5,446	67.4	72.9	0.491	S
	7	B34	J21	J09	J12	J10	J96	J20				3,640	2,472	789	4,087	82.2	59.6	0.489	P
	8	B34	J21	J09	J12	J10	J96	J20	Z03			3,677	2,882	752	3,677	83.0	56.1	0.465	P
	9	B34	J21	J09	J12	J10	J96	J20	Z03	J06		4,019	4,264	410	2,295	90.7	48.5	0.440	P
	10	B34	J21	J09	J12	J10	J96	J20	Z03	J06	B97	4,029	4,265	400	2,294	91.0	48.6	0.442	P
**5-14**	1	J09										90	9	280	1,128	24.3	90.9	0.221	P
	2	J09	B34									170	17	200	1,120	45.9	90.9	0.418	P
	3	J09	B34	J10								244	18	126	1,119	65.9	93.1	0.614	S
	4	J09	B34	J10	B97							247	18	123	1,119	66.8	93.2	0.622	S
	5	J09	B34	J10	B97	J05						255	61	115	1,076	68.9	80.7	0.556	S
	6	J09	B34	J10	B97	J05	J21					261	65	109	1,072	70.5	80.1	0.565	S
	7	J09	B34	J10	B97	J05	J21	J18				286	253	84	884	77.3	53.1	0.410	P
	8	J09	B34	J10	B97	J05	J21	J18	J12			288	270	82	867	77.8	51.6	0.402	P
	9	J09	B34	J10	B97	J05	J21	J18	J12	J11		292	271	78	866	78.9	51.9	0.409	P
	10	J09	B34	J10	B97	J05	J21	J18	J12	J11	J96	300	353	70	784	81.1	45.9	0.373	P
**15-64**	1	J09										753	12	3,152	10,405	19.3	98.4	0.190	P
	2	J09	B34									2,723	75	1,182	10,342	69.7	97.3	0.679	G
	3	J09	B34	B97								2,901	80	1,004	10,337	74.3	97.3	0.723	G
	4	J09	B34	B97	J10							3,043	81	862	10,336	77.9	97.4	0.759	G
	5	J09	B34	B97	J10	J12						3,145	163	760	10,254	80.5	95.1	0.766	G
	6	J09	B34	B97	J10	J12	J20					3,171	244	734	10,173	81.2	92.9	0.754	G
	7	J09	B34	B97	J10	J12	J20	J45				3,298	1,700	607	8,717	84.5	66.0	0.557	S
	8	J09	B34	B97	J10	J12	J20	J45	Z03			3,411	3,307	494	7,110	87.3	50.8	0.444	P
	9	J09	B34	B97	J10	J12	J20	J45	Z03	J21		3,424	3,312	481	7,105	87.7	50.8	0.446	P
	10	J09	B34	B97	J10	J12	J20	J45	Z03	J21	J80	3,427	3,346	478	7,071	87.8	50.6	0.444	P
**65+**	1	B34										5,977	96	4,556	31,148	56.7	98.4	0.558	S
	2	B34	J09									7,335	107	3,198	31,137	69.6	98.6	0.686	G
	3	B34	J09	B97								7,831	114	2,702	31,130	74.3	98.6	0.733	G
	4	B34	J09	B97	J12							8,182	283	2,351	30,961	77.7	96.7	0.751	G
	5	B34	J09	B97	J12	J10						8,243	290	2,290	30,954	78.3	96.6	0.756	G
	6	B34	J09	B97	J12	J10	J20					8,333	419	2,200	30,825	79.1	95.2	0.753	G
	7	B34	J09	B97	J12	J10	J20	Z03				8,687	5,431	1,846	25,813	82.5	61.5	0.507	S

	8	B34	J09	B97	J12	J10	J20	Z03	J15			8,916	9,184	1,617	22,060	84.6	49.3	0.417	P
	9	B34	J09	B97	J12	J10	J20	Z03	J15	J96		9,107	11,602	1,426	19,642	86.5	44.0	0.380	P
	10	B34	J09	B97	J12	J10	J20	Z03	J15	J96	J45	9,192	12,404	1,341	18,840	87.3	42.6	0.371	P

ICELAND

SARI-SARS-CoV-2		ICD 1	ICD 2	ICD 3	ICD 4	ICD 5	ICD 6	ICD 7	ICD 8	ICD 9	ICD 10	TP	FP	FN	TN	Se (%)	PPV(%)	CUI	
All ages	1	U071										1,578	365	54	2,271	96.7	81.2	0.785	G
	2	U071	J96									1,583	616	49	2,020	97.0	72.0	0.698	G
	3	U071	J96	J15								1,592	944	40	1,692	97.5	62.8	0.612	S
	4	U071	J96	J15	J18							1,612	1,510	20	1,126	98.8	51.6	0.510	S
	5	U071	J96	J15	J18	J44						1,622	1,821	10	815	99.4	47.1	0.468	P
	6	U071	J96	J15	J18	J44	J16					1,622	1,821	10	815	99.4	47.1	0.468	P
	7	U071	J96	J15	J18	J44	J16	J90				1,623	1,845	9	791	99.4	46.8	0.465	P
	8	U7.1	J96	J15	J18	J44	J16	J90	J43			1,623	1,852	9	784	99.4	46.7	0.464	P
	9	U7.1	J96	J15	J18	J44	J16	J90	J43	J81		1,623	1,854	9	782	99.4	46.7	0.464	P
	10	U071	J96	J15	J18	J44	J16	J90	J43	J81	J13	1,623	1,875	9	761	99.4	46.4	0.461	P
High SARI	1	U071										1,016	280	30	998	97.1	78.4	0.761	G
	2	U071	J15									1,022	408	24	870	97.7	71.5	0.698	G
	3	U071	J15	J96								1,027	493	19	785	98.2	67.6	0.663	G
	4	U071	J15	J96	J13							1,027	501	19	777	98.2	67.2	0.660	G
	5	U071	J15	J96	J13	J16						1,027	502	19	776	98.2	67.2	0.659	G
	6	U071	J15	J96	J13	J16	J36					1,028	502	18	776	98.3	67.2	0.660	G
	7	U071	J15	J96	J13	J16	J36	J18				1,042	747	4	531	99.6	58.2	0.580	S
	8	U071	J15	J96	J13	J16	J36	J18	J43			1,042	751	4	527	99.6	58.1	0.579	S
	9	U071	J15	J96	J13	J16	J36	J18	J43	J81		1,042	752	4	526	99.6	58.1	0.579	S
	10	U071	J15	J96	J13	J16	J36	J18	J43	J81	J44	1,045	867	1	411	99.9	54.7	0.546	S
Low SARI	1	U071										562	85	24	1,273	95.9	86.9	0.833	E
	2	U071	J96									562	234	24	1,124	95.9	70.6	0.677	G
	3	U071	J96	J16								562	234	24	1,124	95.9	70.6	0.677	G
	4	U071	J96	J16	J01							562	237	24	1,121	95.9	70.3	0.675	G
	5	U U071	J96	J16	J01	J09						564	244	22	1,114	96.2	69.8	0.672	G
	6	U071	J96	J16	J01	J09	J32					564	246	22	1,112	96.2	69.6	0.670	G
	7	U071	J96	J16	J01	J09	J32	J18				571	572	15	786	97.4	50.0	0.487	P
	8	U071	J96	J16	J01	J09	J32	J18	J10			571	578	15	780	97.4	49.7	0.484	P
	9	U071	J96	J16	J01	J09	J32	J18	J10	U072		571	588	15	770	97.4	49.3	0.480	P
	10	U071	J96	J16	J01	J09	J32	J18	J10	U072	J44	579	802	7	556	98.8	41.9	0.414	P
0-4	1	U071										65	3	3	485	95.6	95.6	0.914	E
	2	U071	J10									65	12	3	476	95.6	84.4	0.807	G
	3	U071	J10	J02								65	22	3	466	95.6	74.7	0.714	G
	4	U071	J10	J02	J21							67	209	1	279	98.5	24.3	0.239	P
	5	U071	J10	J02	J21	J00						67	221	1	267	98.5	23.3	0.229	P
	6	U071	J10	J02	J21	J00	J09					67	227	1	261	98.5	22.8	0.225	P
	7	U071	J10	J02	J21	J00	J09	J05				67	236	1	252	98.5	22.1	0.218	P
	8	U071	J10	J02	J21	J00	J09	J05	J20			68	313	0	175	100.0	17.8	0.178	P
	9	U071	J10	J02	J21	J00	J09	J05	J20	J18		68	345	0	143	100.0	16.5	0.165	P
	10																		
5-14	1	U071										65	3	3	485	95.6	95.6	0.914	E
	2	U071	J12									65	44	3	444	95.6	59.6	0.570	S
	3	U071	J12	J15								65	91	3	397	95.6	41.7	0.398	P
	4																		
	5																		
	6																		
	7																		
	8																		
	9																		
	10																		
15-64	1	U071										534	83	14	538	97.4	86.5	0.843	E
	2	U071	J96									534	180	14	441	97.4	74.8	0.729	G
	3	U071	J96	J16								534	180	14	441	97.4	74.8	0.729	G
	4	U071	J96	J16	J15							536	255	12	366	97.8	67.8	0.663	G
	5	U071	J96	J16	J15	J69						537	258	11	363	98.0	67.5	0.662	G

	6	U071	J96	J16	J15	J69	J01					537	260	11	361	98.0	67.4	0.660	G
	7	U071	J96	J16	J15	J69	J01	J09				539	268	9	353	98.4	66.8	0.657	G
	8	U071	J96	J16	J15	J69	J01	J09	J81			539	269	9	352	98.4	66.7	0.656	G
	9	U071	J96	J16	J15	J69	J01	J09	J81	J94		540	270	8	351	98.5	66.7	0.657	G
	10	U071	J96	J16	J15	J69	J01	J09	J81	J94	J18	545	412	3	209	99.5	56.9	0.566	S
65+	1	U071										964	277	37	1,185	96.3	77.7	0.748	G
	2	U071	J96									969	428	32	1,034	96.8	69.4	0.671	G
	3	U071	J96	J16								969	429	32	1,033	96.8	69.3	0.671	G
	4	U071	J96	J16	J15							976	625	25	837	97.5	61.0	0.594	S
	5	U071	J96	J16	J15	J90						978	648	23	814	97.7	60.1	0.588	S
	6	U071	J96	J16	J15	J90	J13					978	658	23	804	97.7	59.8	0.584	S
	7	U071	J96	J16	J15	J90	J13	J18				991	1,001	10	461	99.0	49.7	0.493	S
	8	U071	J96	J16	J15	J90	J13	J18	J43			991	1,005	10	457	99.0	49.6	0.492	S
	9	U071	J96	J16	J15	J90	J13	J18	J43	J81		991	1,006	10	456	99.0	49.6	0.491	S
	10	U071	J96	J16	J15	J90	J13	J18	J43	J81	J02/ J80	991	1,008	10	454	99.0	49.6	0.491	S

ICELAND

SARI-Influenza		ICD 1	ICD 2	ICD 3	ICD 4	ICD 5	ICD 6	ICD 7	ICD 8	ICD 9	ICD 10	TP	FP	FN	TN	Se (%)	PPV(%)	CUI	
All ages	1	J10										66	40	179	1,615	36.9	62.3	0.230	P
	2	J10	J09									102	68	77	1,587	57.0	60.0	0.342	P
	3	J10	J09	J11								128	86	51	1,569	71.5	59.8	0.428	P
	4	J10	J09	J11	J18							149	483	30	1,172	83.2	23.6	0.196	P
	5	J10	J09	J11	J18	U071						153	568	26	1,087	85.5	21.2	0.181	P
	6	J10	J09	J11	J18	U071	J91					153	569	26	1,086	85.5	21.2	0.181	P
	7	J10	J09	J11	J18	U071	J91	J94				153	571	26	1,084	85.5	21.1	0.181	P
	8	J10	J09	J11	J18	U071	J91	J94	J43			153	579	26	1,076	85.5	20.9	0.179	P
	9	J10	J09	J11	J18	U071	J91	J94	J43	J04		154	583	25	1,072	86.0	20.9	0.180	P
	10	J10	J09	J11	J18	U071	J91	J94	J43	J04	J14/ J85	155	588	24	1,067	86.6	20.9	0.181	P
High SARI	1	J10										56	33	79	700	41.5	62.9	0.261	P
	2	J10	J09									82	54	53	679	60.7	60.3	0.366	P
	3	J10	J09	J11								104	68	31	665	77.0	60.5	0.466	P
	4	J10	J09	J11	J18							114	229	21	504	84.4	33.2	0.281	P
	5	J10	J09	J11	J18	J44						116	321	19	412	85.9	26.5	0.228	P
	6	J10	J09	J11	J18	J44	J96					120	347	15	386	88.9	25.7	0.228	P
	7	J10	J09	J11	J18	J44	J96	J43				120	350	15	383	88.9	25.5	0.227	P
	8	J10	J09	J11	J18	J44	J96	J43	J03			121	357	14	376	89.6	25.3	0.227	P
	9	J10	J09	J11	J18	J44	J96	J43	J03	J04		122	359	13	374	90.4	25.4	0.229	P
	10	J10	J09	J11	J18	J44	J96	J43	J03	J04	J14	123	361	12	372	91.1	25.4	0.232	P
Low SARI	1	J09										10	7	34	915	22.7	58.8	0.134	P
	2	J09	J10									20	14	24	908	45.5	58.8	0.267	P
	3	J09	J10	J11								24	18	20	904	54.5	57.1	0.312	P
	4	J09	J10	J11	J18							35	254	9	668	79.5	12.1	0.096	P
	5	J09	J10	J11	J18	J85						35	254	9	668	79.5	12.1	0.096	P
	6	J09	J10	J11	J18	J85	J94					35	255	9	667	79.5	12.1	0.096	P
	7	J09	J10	J11	J18	J85	J94	J91				35	256	9	666	79.5	12.0	0.096	P
	8	J09	J10	J11	J18	J85	J94	J91	J86			35	260	9	662	79.5	11.9	0.094	P
	9	JJ09	J10	J11	J18	J85	J94	J91	J86	U071		35	289	9	633	79.5	10.8	0.086	P
	10	J09	J10	J11	J18	J85	J94	J91	J86	U071	J44	38	456	6	466	86.4	7.7	0.066	P
0-4	1	J10										10	8	11	462	47.6	55.6	0.265	P
	2	J10	U071									11	10	10	460	52.4	52.4	0.274	P
	3	J10	U071	J09								16	15	5	455	76.2	51.6	0.393	P
	4	J10	U071	J09	J91							17	16	4	454	81.0	51.5	0.417	P
	5	J10	U071	J09	J91	J94						17	16	4	454	81.0	51.5	0.417	P
	6	J10	U071	J09	J91	J94	J18					17	66	4	404	81.0	20.5	0.166	P
	7	J10	U071	J09	J91	J94	J18	J85				17	68	4	402	81.0	20.0	0.162	P
	8	J10	U071	J09	J91	J94	J18	J85	J86			17	70	4	400	81.0	19.5	0.158	P
	9	J10	U071	J09	J91	J94	J18	J85	J86	J03		17	76	4	394	81.0	18.3	0.148	P
	10	J10	U071	J09	J91	J94	J18	J85	J86	J03	J15	19	120	2	350	90.5	13.7	0.124	P
5-14	1	J10										2	1	5	58	28.6	66.7	0.190	P
	2	J10	J11									3	1	4	58	42.9	75.0	0.321	P
	3	J10	J11	J03								4	2	3	57	57.1	66.7	0.381	P

	4	J10	J11	J03	J04							5	3	2	56	71.4	62.5	0.446	P
	5	J10	J11	J03	J04	J18						7	19	0	40	100.0	26.9	0.269	P
	6	J10	J11	J03	J04	J18	J06					7	22	0	37	100.0	24.1	0.241	P
	7																		
	8																		
	9																		
	10																		
15-64	1	J10										20	12	27	314	42.6	62.5	0.266	P
	2	J10	J09									29	17	18	309	61.7	63.0	0.389	P
	3	J10	J09	J11								33	20	14	306	70.2	62.3	0.437	P
	4	J10	J09	J11	J20							35	26	12	300	74.5	57.4	0.427	P
	5	J10	J09	J11	J20	J96						37	82	10	244	78.7	31.1	0.245	P
	6	J10	J09	J11	J20	J96	U071					38	98	9	228	80.9	27.9	0.226	P
	7	J10	J09	J11	J20	J96	U071	J14				39	99	8	227	83.0	28.3	0.235	P
	8	J10	J09	J11	J20	J96	U071	J14	J18			45	187	2	139	95.7	19.4	0.186	P
	9	J10	J09	J11	J20	J96	U071	J14	J18	J44		46	223	1	103	97.9	17.1	0.167	P
	10	J10	J09	J11	J20	J96	U071	J14	J18	J44	J90	46	228	1	98	97.9	16.8	0.164	P
65+	1	J10										34	19	70	781	32.7	64.2	0.210	P
	2	J10	J09									56	37	48	763	53.8	60.2	0.324	P
	3	J10	J09	J11								77	52	27	748	74.0	59.7	0.442	P
	4	J10	J09	J11	J21							77	52	27	748	74.0	59.7	0.442	P
	5	J10	J09	J11	J21	J18						89	288	15	512	85.6	23.6	0.202	P
	6	J10	J09	J11	J21	J18	J43					89	294	15	506	85.6	23.2	0.199	P
	7	J10	J09	J11	J21	J18	J43	J05				90	296	14	504	86.5	23.3	0.202	P
	8	J10	J09	J11	J21	J18	J43	J05	J20			92	324	0	476	100.0	22.1	0.221	P
	9	J10	J09	J11	J21	J18	J43	J05	J20	J44		96	531	8	269	92.3	15.3	0.141	P
	10	J10	J09	J11	J21	J18	J43	J05	J20	J44	J13	97	535	7	265	93.3	15.3	0.143	P

MALTA

SARI-SARS-CoV-2		ICD 1	ICD 2	ICD 3	ICD 4	ICD 5	ICD 6	ICD 7	ICD 8	ICD 9	ICD 10	TP	FP	FN	TN	Se (%)	PPV(%)	CUI	
All ages	1	U071										2,027	219	199	7,058	91.1	90.2	0.822	E
	2	U071	J18									2,078	1,867	148	5,410	93.4	52.7	0.492	S
	3	U071	J18	J15								2,079	1,885	147	5,392	93.4	52.4	0.490	P
	4	U071	J18	J15	J02							2,085	1,916	141	5,361	93.7	52.1	0.488	P
	5	U071	J18	J15	J02	J04						2,087	1,924	139	5,353	93.8	52.0	0.488	P
	6	U071	J18	J15	J02	J04	J69					2,125	2,559	101	4,718	95.5	45.4	0.433	P
	7	U071	J18	J15	J02	J04	J69	J45				2,140	3,115	86	4,162	96.1	40.7	0.391	P
	8	U071	J18	J15	J02	J04	J69	J45	J06			2,145	3,420	81	3,857	96.4	38.5	0.371	P
	9	U071	J18	J15	J02	J04	J69	J45	J06	J84		2,148	3,593	78	3,684	96.5	37.4	0.361	P
	10	U071	J18	J15	J02	J04	J69	J45	J06	J84	J80	2,148	3,607	78	3,670	96.5	37.3	0.360	P
High SARI	1	U071										399	38	33	998	92.4	91.3	0.843	E
	2	U071	J18									409	269	23	767	94.7	60.3	0.571	S
	3	U071	J18	J03								411	276	21	760	95.1	59.8	0.569	S
	4	U071	J18	J03	J02							413	279	19	757	95.6	59.7	0.571	S
	5	U071	J18	J03	J02	J69						421	352	11	684	97.5	54.5	0.531	S
	6	U071	J18	J03	J02	J69	J04					421	353	11	683	97.5	54.4	0.530	S
	7	U071	J18	J03	J02	J69	J04	J80				421	354	11	682	97.5	54.3	0.529	S
	8	U071	J18	J03	J02	J69	J04	J80	J34			422	356	10	680	97.7	54.2	0.530	S
	9	U071	J18	J03	J02	J69	J04	J80	J34	J84		422	376	10	660	97.7	52.9	0.517	S
	10	U071	J18	J03	J02	J69	J04	J80	J34	J84	J06	423	434	9	602	97.9	49.4	0.483	P
Low SARI	1	U071										1,628	181	166	6,060	90.7	90.0	0.817	E
	2	U071	J18									1,669	1,598	125	4,643	93.0	51.1	0.475	P
	3	U071	J18	J15								1,670	1,614	124	4,627	93.1	50.9	0.473	P
	4	U071	J18	J15	J02							1,674	1,642	120	4,599	93.3	50.5	0.471	P
	5	U071	J18	J15	J02	J04						1,676	1,649	118	4,592	93.4	50.4	0.471	P
	6	U071	J18	J15	J02	J04	J45					1,690	2,125	104	4,116	94.2	44.3	0.417	P
	7	U071	J18	J15	J02	J04	J45	J69				1,720	2,680	74	3,561	95.9	39.1	0.375	P
	8	U071	J18	J15	J02	J04	J45	J69	J93			1,724	2,763	70	3,478	96.1	38.4	0.369	P
	9	U071	J18	J15	J02	J04	J45	J69	J93	J34		1,725	2,771	69	3,470	96.2	38.4	0.369	P
	10	U071	J18	J15	J02	J04	J45	J69	J93	J34	J06	1,729	3,019	65	3,222	96.4	36.4	0.351	P
0-4	1	U071										125	4	6	511	95.4	96.9	0.925	E

	2	U071	J18									127	27	4	488	96.9	82.5	0.799	G
	3	U071	J18	J06								127	176	4	339	96.9	41.9	0.406	P
	4	U071	J18	J06	J69							128	178	3	337	97.7	41.8	0.409	P
	5	U071	J18	J06	J69	J05						128	200	3	315	97.7	39.0	0.381	P
	6	U071	J18	J06	J69	J05	J12					129	205	2	310	98.5	38.6	0.380	P
	7	U071	J18	J06	J69	J05	J12	J21				129	345	2	170	98.5	27.2	0.268	P
	8	U071	J18	J06	J69	J05	J12	J21	J02			129	364	2	151	98.5	26.2	0.258	P
	9	U071	J18	J06	J69	J05	J12	J21	J02	J03		130	402	1	113	99.2	24.4	0.242	P
	10	U071	J18	J06	J69	J05	J12	J21	J02	J03	J22	131	498	0	17	100.0	20.8	0.208	P
5-14	1	U071										22	0	0	98	100.0	100.0	1.000	E
	2	U071	J18									22	5	0	93	100.0	81.5	0.815	E
	3	U071	J18	J06								22	14	0	84	100.0	61.1	0.611	S
	4																		
	5																		
	6																		
	7																		
	8																		
	9																		
	10																		
15-64	1	U071										630	49	50	1,832	92.6	92.8	0.860	E
	2	U071	J18									635	422	45	1,459	93.4	60.1	0.561	S
	3	U071	J18	J02								639	432	41	1,449	94.0	59.7	0.561	S
	4	U071	J18	J02	J45							645	728	35	1,153	94.9	47.0	0.446	P
	5	U071	J18	J02	J45	J34						647	732	33	1,149	95.1	46.9	0.446	P
	6	U071	J18	J02	J45	J34	J15					647	736	33	1,145	95.1	46.8	0.445	P
	7	U071	J18	J02	J45	J34	J15	J96				650	821	30	1,060	95.6	44.2	0.422	P
	8	U071	J18	J02	J45	J34	J15	J96	J69			655	902	25	979	96.3	42.1	0.405	P
	9	U071	J18	J02	J45	J34	J15	J96	J69	J80		655	908	25	973	96.3	41.9	0.404	P
	10	U071	J18	J02	J45	J34	J15	J96	J69	J80	J04	656	913	24	968	96.5	41.8	0.403	P
65+	1	U071										1,250	166	143	4,617	89.7	88.3	0.792	G
	2	U071	J18									1,294	1,413	99	3,370	92.9	47.8	0.444	P
	3	U071	J18	J15								1,295	1,427	98	3,356	93.0	47.6	0.442	P
	4	U071	J18	J15	J02							1,297	1,427	96	3,356	93.1	47.6	0.443	P
	5	U071	J18	J15	J02	J05						1,297	1,427	96	3,356	93.1	47.6	0.443	P
	6	U071	J18	J15	J02	J05	J04					1,298	1,429	95	3,354	93.2	47.6	0.444	P
	7	U071	J18	J15	J02	J05	J04	J13				1,298	1,429	95	3,354	93.2	47.6	0.444	P
	8	U071	J18	J15	J02	J05	J04	J13	J69			1,330	1,970	63	2,813	95.5	40.3	0.385	P
	9	U071	J18	J15	J02	J05	J04	J13	J69	J45		1,339	2,184	54	2,599	96.1	38.0	0.365	P
	10	U071	J18	J15	J02	J05	J04	J13	J69	J45	J84	1,340	2,329	53	2,454	96.2	36.5	0.351	P

MALTA

SARI-Influenza		ICD 1	ICD 2	ICD 3	ICD 4	ICD 5	ICD 6	ICD 7	ICD 8	ICD 9	ICD 10	TP	FP	FN	TN	Se (%)	PPV(%)	CUI	
All ages	1	J10										182	11	221	6,536	45.2	94.3	0.426	P
	2	J10	J11									224	16	179	6,531	55.6	93.3	0.519	S
	3	J10	J11	J22								287	1,474	116	5,073	71.2	16.3	0.116	P
	4	J10	J11	J22	J06							311	1,779	92	4,768	77.2	14.9	0.115	P
	5	J10	J11	J22	J06	J01						313	1,782	90	4,765	77.7	14.9	0.116	P
	6	J10	J11	J22	J06	J01	J15					315	1,805	88	4,742	78.2	14.9	0.116	P
	7	J10	J11	J22	J06	J01	J15	J18				339	3,408	64	3,139	84.1	9.0	0.076	P
	8	J10	J11	J22	J06	J01	J15	J18	J44			366	4,427	37	2,120	90.8	7.6	0.069	P
	9	J10	J11	J22	J06	J01	J15	J18	J44	J45		381	4,926	22	1,621	94.5	7.2	0.068	P
	10	J10	J11	J22	J06	J01	J15	J18	J44	J45	J20	385	5,043	18	1,504	95.5	7.1	0.068	P
High SARI	1	J10										62	1	62	1,853	50.0	98.4	0.492	S
	2	J10	J11									75	4	49	1,850	60.5	94.9	0.574	S
	3	J10	J11	J06								85	120	39	1,734	68.5	41.5	0.284	P
	4	J10	J11	J06	J22							100	597	24	1,257	80.6	14.3	0.116	P
	5	J10	J11	J06	J22	J96						105	693	19	1,161	84.7	13.2	0.111	P
	6	J10	J11	J06	J22	J96	J01					106	695	18	1,159	85.5	13.2	0.113	P
	7	J10	J11	J06	J22	J96	J01	J32				106	697	18	1,157	85.5	13.2	0.113	P
	8	J10	J11	J06	J22	J96	J01	J32	J13			106	702	18	1,152	85.5	13.1	0.112	P
	9	J10	J11	J06	J22	J96	J01	J32	J13	J18		113	1,066	11	788	91.1	9.6	0.087	P
	10	J10	J11	J06	J22	J96	J01	J32	J13	J18	J84	113	1,100	11	754	91.1	9.3	0.085	P

**Low SARI**	1	J10										120	10	159	4,683	43.0	92.3	0.397	P
	2	J10	J11									149	12	130	4,681	53.4	92.5	0.494	S
	3	J10	J11	J22								197	993	82	3,700	70.6	16.6	0.117	P
	4	J10	J11	J22	J06							211	1,182	68	3,511	75.6	15.1	0.115	P
	5	J10	J11	J22	J06	J20						216	1,261	63	3,432	77.4	14.6	0.113	P
	6	J10	J11	J22	J06	J20	J15					218	1,278	61	3,415	78.1	14.6	0.114	P
	7	J10	J11	J22	J06	J20	J15	J44				240	2,071	39	2,622	86.0	10.4	0.089	P
	8	J10	J11	J22	J06	J20	J15	J44	J45			252	2,433	27	2,260	90.3	9.4	0.085	P
	9	J10	J11	J22	J06	J20	J15	J44	J45	J01		253	2,434	26	2,259	90.7	9.4	0.085	P
	10	J10	J11	J22	J06	J20	J15	J44	J45	J01	J18	268	3,590	11	1,103	96.1	6.9	0.067	P
**0-4**	1	J10										12	3	14	553	46.2	80.0	0.369	P
	2	J10	J06									18	112	8	444	69.2	13.8	0.096	P
	3	J10	J06	J11								19	112	7	444	73.1	14.5	0.106	P
	4	J10	J06	J11	J22							23	235	3	321	88.5	8.9	0.079	P
	5	J10	J06	J11	J22	J21						26	428	0	128	100.0	5.7	0.057	P
	6																		
	7																		
	8																		
	9																		
	10																		
**5-14**	1	J10										8	0	11	109	42.1	100.0	0.421	P
	2	J10	J11									11	0	8	109	57.9	100.0	0.579	S
	3	J10	J11	J22								15	24	4	85	78.9	38.5	0.304	P
	4	J10	J11	J22	J21							16	24	3	85	84.2	40.0	0.337	P
	5	J10	J11	J22	J21	J90						16	24	3	85	84.2	40.0	0.337	P
	6	J10	J11	J22	J21	J90	J12					16	25	3	84	84.2	39.0	0.329	P
	7	J10	J11	J22	J21	J90	J12	J06				18	40	1	69	94.7	31.0	0.294	P
	8	J10	J11	J22	J21	J90	J12	J06	J03			19	53	0	56	100.0	26.4	0.264	P
	9																		
	10																		
**15-64**	1	J10										55	3	62	1,548	47.0	94.8	0.446	P
	2	J10	J11									68	4	49	1,547	58.1	94.4	0.549	S
	3	J10	J11	J22								85	280	32	1,271	72.6	23.3	0.169	P
	4	J10	J11	J22	J01							87	283	30	1,268	74.4	23.5	0.175	P
	5	J10	J11	J22	J01	J80						87	288	30	1,263	74.4	23.2	0.173	P
	6	J10	J11	J22	J01	J80	J20					89	320	28	1,231	76.1	21.8	0.166	P
	7	J10	J11	J22	J01	J80	J20	J32				89	326	28	1,225	76.1	21.4	0.163	P
	8	J10	J11	J22	J01	J80	J20	J32	J15			89	332	28	1,219	76.1	21.1	0.161	P
	9	J10	J11	J22	J01	J80	J20	J32	J15	J21		90	339	27	1,212	76.9	21.0	0.161	P
	10	J10	J11	J22	J01	J80	J20	J32	J15	J21	J45	99	598	18	953	84.6	14.2	0.120	P
**65+**	1	J10										107	5	134	4,326	44.4	95.5	0.424	P
	2	J10	J11									132	9	109	4,322	54.8	93.6	0.513	S
	3	J10	J11	J22								170	1,044	71	3,287	70.5	14.0	0.099	P
	4	J10	J11	J22	J45							179	1,269	62	3,062	74.3	12.4	0.092	P
	5	J10	J11	J22	J45	J06						189	1,373	52	2,958	78.4	12.1	0.095	P
	6	J10	J11	J22	J45	J06	J44					212	2,216	29	2,115	88.0	8.7	0.077	P
	7	J10	J11	J22	J45	J06	J44	J18				227	3,353	14	978	94.2	6.3	0.060	P
	8	J10	J11	J22	J45	J06	J44	J18	J13			227	3,355	14	976	94.2	6.3	0.060	P
	9	J10	J11	J22	J45	J06	J44	J18	J13	J15		228	3,366	13	965	94.6	6.3	0.060	P
	10	J10	J11	J22	J45	J06	J44	J18	J13	J15	J20	231	3,448	10	883	95.9	6.3	0.060	P

MALTA

SARI-RSV		ICD 1	ICD 2	ICD 3	ICD 4	ICD 5	ICD 6	ICD 7	ICD 8	ICD 9	ICD 10	TP	FP	FN	TN	Se (%)	PPV(%)	CUI	
**All ages**	1	J21										111	117	210	6,512	34.6	48.7	0.168	P
	2	J21	J20									126	233	195	6,396	39.3	35.1	0.138	P
	3	J21	J20	J14								127	234	194	6,395	39.6	35.2	0.139	P
	4	J21	J20	J14	J12							134	305	187	6,324	41.7	30.5	0.127	P
	5	J21	J20	J14	J12	J22						206	1,759	115	4,870	64.2	10.5	0.067	P
	6	J21	J20	J14	J12	J22	J61					207	1,762	114	4,867	64.5	10.5	0.068	P
	7	J21	J20	J14	J12	J22	J61	J40				209	1,798	112	4,831	65.1	10.4	0.068	P
	8	J21	J20	J14	J12	J22	J61	J40	J06			226	2,107	95	4,522	70.4	9.7	0.068	P
	9	J21	J20	J14	J12	J22	J61	J40	J06	J38		226	2,119	95	4,510	70.4	9.6	0.068	P

	10	J21	J20	J14	J12	J22	J61	J40	J06	J38	J05	227	2,127	94	4,502	70.7	9.6	0.068	P
**High SARI**	1	J21										13	37	62	1,866	17.3	26.0	0.045	P
	2	J21	J22									32	520	43	1,383	42.7	5.8	0.025	P
	3	J21	J22	J14								33	520	42	1,383	44.0	6.0	0.026	P
	4	J21	J22	J14	J45							42	680	33	1,223	56.0	5.8	0.033	P
	5	J21	J22	J14	J45	J61						42	681	33	1,222	56.0	5.8	0.033	P
	6	J21	J22	J14	J45	J61	J20					47	719	28	1,184	62.7	6.1	0.038	P
	7	J21	J22	J14	J45	J61	J20	J44				58	1,009	17	894	77.3	5.4	0.042	P
	8	J21	J22	J14	J45	J61	J20	J44	J67			59	1,012	16	891	78.7	5.5	0.043	P
	9	J21	J22	J14	J45	J61	J20	J44	J67	J06		66	1,125	9	778	88.0	5.5	0.049	P
	10	J21	J22	J14	J45	J61	J20	J44	J67	J06	J05	67	1,127	8	776	89.3	5.6	0.050	P
**Low SARI**	1	J21										98	80	148	4,646	39.8	55.1	0.219	P
	2	J21	J20									108	156	138	4,570	43.9	40.9	0.180	P
	3	J21	J20	J12								114	198	132	4,528	46.3	36.5	0.169	P
	4	J21	J20	J12	J22							167	1,177	79	3,549	67.9	12.4	0.084	P
	5	J21	J20	J12	J22	J40						169	1,200	77	3,526	68.7	12.3	0.085	P
	6	J21	J20	J12	J22	J40	J45					184	1,583	62	3,143	74.8	10.4	0.078	P
	7	J21	J20	J12	J22	J40	J45	J06				194	1,770	52	2,956	78.9	9.9	0.078	P
	8	J21	J20	J12	J22	J40	J45	J06	J18			219	2,969	27	1,757	89.0	6.9	0.061	P
	9	J21	J20	J12	J22	J40	J45	J06	J18	J44		236	3,709	10	1,017	95.9	6.0	0.057	P
	10	J21	J20	J12	J22	J40	J45	J06	J18	J44	J38	236	3,717	10	1,009	95.9	6.0	0.057	P
**0-4**	1	J21										105	101	41	335	71.9	51.0	0.367	P
	2	J21	J22									128	202	18	234	87.7	38.8	0.340	P
	3	J21	J22	J12								132	206	14	230	90.4	39.1	0.353	P
	4	J21	J22	J12	J20							133	211	13	225	91.1	38.7	0.352	P
	5	J21	J22	J12	J20	J06						141	316	5	120	96.6	30.9	0.298	P
	6	J21	J22	J12	J20	J06	J18					145	338	1	98	99.3	30.0	0.298	P
	7	J21	J22	J12	J20	J06	J18	J05				146	344	0	92	100.0	29.8	0.298	P
	8	J21	J22	J12	J20	J06	J18	J05	J03			146	371	0	65	100.0	28.2	0.282	P
	9																		
	10																		
**5-14**	1	J06										2	15	2	109	50.0	11.8	0.059	P
	2	J06	J18									3	18	1	106	75.0	14.3	0.107	P
	3	J06	J18	J45								4	69	0	55	100.0	5.5	0.055	P
	4																		
	5																		
	6																		
	7																		
	8																		
	9																		
	10																		
**15-64**	1	J21										4	4	35	1,625	10.3	50.0	0.051	P
	2	J21	J45									13	283	26	1,346	33.3	4.4	0.015	P
	3	J21	J45	J40								13	295	26	1,334	33.3	4.2	0.014	P
	4	J21	J45	J40	J98							14	299	25	1,330	35.9	4.5	0.016	P
	5	J21	J45	J40	J98	J44						22	536	17	1,093	56.4	3.9	0.022	P
	6	J21	J45	J40	J98	J44	J13					23	539	16	1,090	59.0	4.1	0.024	P
	7	J21	J45	J40	J98	J44	J13	J67				24	544	15	1,085	61.5	4.2	0.026	P
	8	J21	J45	J40	J98	J44	J13	J67	J90			26	589	13	1,040	66.7	4.2	0.028	P
	9	J21	J45	J40	J98	J44	J13	J67	J90	J12		26	605	13	1,024	66.7	4.1	0.027	P
	10	J21	J45	J40	J98	J44	J13	J67	J90	J12	J22	31	865	8	764	79.5	3.5	0.028	P
**65+**	1	J20										13	75	119	4,365	9.8	14.8	0.015	P
	2	J20	J45									28	313	104	4,127	21.2	8.2	0.017	P
	3	J20	J45	J14								29	314	103	4,126	22.0	8.5	0.019	P
	4	J20	J45	J14	J22							71	1,352	61	3,088	53.8	5.0	0.027	P
	5	J20	J45	J14	J22	J21						73	1,361	59	3,079	55.3	5.1	0.028	P
	6	J20	J45	J14	J22	J21	J61					73	1,363	59	3,077	55.3	5.1	0.028	P
	7	J20	J45	J14	J22	J21	J61	J44				95	2,218	37	2,222	72.0	4.1	0.030	P
	8	J20	J45	J14	J22	J21	J61	J44	J40			97	2,244	35	2,196	73.5	4.1	0.030	P
	9	J20	J45	J14	J22	J21	J61	J44	J40	J38		97	2,252	35	2,188	73.5	4.1	0.030	P
	10	J20	J45	J14	J22	J21	J61	J44	J40	J38	J18	115	3,398	17	1,042	87.1	3.3	0.029	P

MALTA

SARI- any of the three		ICD 1	ICD 2	ICD 3	ICD 4	ICD 5	ICD 6	ICD 7	ICD 8	ICD 9	ICD 10	TP	FP	FN	TN	Se (%)	PPV(%)	CUI	
All ages	1	U071										334	80	377	3,695	47.0	80.7	0.379	P
	2	U071	J10									415	82	296	3,693	58.4	83.5	0.487	P
	3	U071	J10	J21								474	139	237	3,636	66.7	77.3	0.515	S
	4	U071	J10	J21	J11							496	141	215	3,634	69.8	77.9	0.543	S
	5	U071	J10	J21	J11	J18						548	1,152	163	2,623	77.1	32.2	0.248	P
	6	U071	J10	J21	J11	J18	J34					549	1,152	162	2,623	77.2	32.3	0.249	P
	7	U071	J10	J21	J11	J18	J34	J22				614	1,980	97	1,795	86.4	23.7	0.204	P
	8	U071	J10	J21	J11	J18	J34	J22	J06			633	2,136	78	1,639	89.0	22.9	0.204	P
	9	U071	J10	J21	J11	J18	J34	J22	J06	J01		635	2,138	76	1,637	89.3	22.9	0.205	P
	10	U071	J10	J21	J11	J18	J34	J22	J06	J01	J45	654	2,436	57	1,339	92.0	21.2	0.195	P
High SARI	1	U071										86	16	70	705	55.1	84.3	0.465	P
	2	U071	J10									104	16	52	705	66.7	86.7	0.578	S
	3	U071	J10	J11								110	17	46	704	70.5	86.6	0.611	S
	4	U071	J10	J11	J18							120	186	36	535	76.9	39.2	0.302	P
	5	U071	J10	J11	J18	J21						126	196	30	525	80.8	39.1	0.316	P
	6	U071	J10	J11	J18	J21	J34					127	196	29	525	81.4	39.3	0.320	P
	7	U071	J10	J11	J18	J21	J34	J06				134	242	22	479	85.9	35.6	0.306	P
	8	U071	J10	J11	J18	J21	J34	J06	J45			139	306	17	415	89.1	31.2	0.278	P
	9	U071	J10	J11	J18	J21	J34	J06	J45	J01		140	307	16	414	89.7	31.3	0.281	P
	10	U071	J10	J11	J18	J21	J34	J06	J45	J01	J02	140	308	16	413	89.7	31.3	0.280	P
Low SARI	1	U071										248	64	307	2,990	44.7	79.5	0.355	P
	2	U071	J10									311	66	244	2,988	56.0	82.5	0.462	P
	3	U071	J10	J21								364	113	191	2,941	65.6	76.3	0.500	S
	4	U071	J10	J21	J11							380	114	175	2,940	68.5	76.9	0.527	S
	5	U071	J10	J21	J11	J18						422	956	133	2,098	76.0	30.6	0.233	P
	6	U071	J10	J21	J11	J18	J22					479	1,587	76	1,467	86.3	23.2	0.200	P
	7	U071	J10	J21	J11	J18	J22	J15				480	1,597	75	1,457	86.5	23.1	0.200	P
	8	U071	J10	J21	J11	J18	J22	J15	J01			481	1,598	74	1,456	86.7	23.1	0.201	P
	9	U071	J10	J21	J11	J18	J22	J15	J01	J45		496	1,837	59	1,217	89.4	21.3	0.190	P
	10	U071	J10	J21	J11	J18	J22	J15	J01	J45	J20	506	1,881	49	1,173	91.2	21.2	0.193	P
0-4	1	U071										23	3	78	165	22.8	88.5	0.201	P
	2	U071	J21									77	50	24	118	76.2	60.6	0.462	P
	3	U071	J21	J22								91	90	10	78	90.1	50.3	0.453	P
	4	U071	J21	J22	J10							92	90	9	78	91.1	50.5	0.460	P
	5	U071	J21	J22	J10	J06						98	139	3	29	97.0	41.4	0.401	P
	6	U071	J21	J22	J10	J06	J12					99	140	2	28	98.0	41.4	0.406	P
	7	U071	J21	J22	J10	J06	J12	J20				99	141	2	27	98.0	41.3	0.404	P
	8	U071	J21	J22	J10	J06	J12	J20	J18			101	154	0	14	100.0	39.6	0.396	P
	9	U071	J21	J22	J10	J06	J12	J20	J18	J03		101	159	0	9	100.0	38.8	0.388	P
	10																		
5-14	1	J10										1	0	7	43	12.5	100.0	0.125	P
	2	J10	J11									2	0	6	43	25.0	100.0	0.250	P
	3	J10	J11	J21								3	0	5	43	37.5	100.0	0.375	P
	4	J10	J11	J21	U071							4	0	4	43	50.0	100.0	0.500	S
	5	J10	J11	J21	U071	J06						6	3	2	40	75.0	66.7	0.500	S
	6	J10	J11	J21	U071	J06	J18					7	4	1	39	87.5	63.6	0.557	S
	7	J10	J11	J21	U071	J06	J18	J45				8	26	0	17	100.0	23.5	0.235	P
	8																		
	9																		
	10																		
15-64	1	U071										97	23	93	969	51.1	80.8	0.413	P
	2	U071	J10									128	23	62	969	67.4	84.8	0.571	S
	3	U071	J10	J11								136	24	54	968	71.6	85.0	0.608	S
	4	U071	J10	J11	J18							147	272	43	720	77.4	35.1	0.271	P
	5	U071	J10	J11	J18	J34						148	272	42	720	77.9	35.2	0.274	P
	6	U071	J10	J11	J18	J34	J21					150	273	40	719	78.9	35.5	0.280	P

		ICD 1	ICD 2	ICD 3	ICD 4	ICD 5	ICD 6	ICD 7	ICD 8	ICD 9	ICD 10	TP	FP	FN	TN	Se (%)	PPV(%)	CUI	
	7	U071	J10	J11	J18	J34	J21	J22				163	448	27	544	85.8	26.7	0.229	P
	8	U071	J10	J11	J18	J34	J21	J22	J01			165	450	25	542	86.8	26.8	0.233	P
	9	U071	J10	J11	J18	J34	J21	J22	J01	J45		174	614	16	378	91.6	22.1	0.202	P
	10	U071	J10	J11	J18	J34	J21	J22	J01	J45	J80	174	616	16	376	91.6	22.0	0.202	P
65+	1	U071										213	54	199	2,518	51.7	79.8	0.412	P
	2	U071	J10									260	55	152	2,517	63.1	82.5	0.521	S
	3	U071	J10	J11								273	56	139	2,516	66.3	83.0	0.550	S
	4	U071	J10	J11	J18							312	803	100	1,769	75.7	28.0	0.212	P
	5	U071	J10	J11	J18	J13						312	803	100	1,769	75.7	28.0	0.212	P
	6	U071	J10	J11	J18	J13	J95					312	803	100	1,769	75.7	28.0	0.212	P
	7	U071	J10	J11	J18	J13	J95	J45				326	921	86	1,651	79.1	26.1	0.207	P
	8	U071	J10	J11	J18	J13	J95	J45	J15			327	930	85	1,642	79.4	26.0	0.206	P
	9	U071	J10	J11	J18	J13	J95	J45	J15	J20		335	970	77	1,602	81.3	25.7	0.209	P
	10	U071	J10	J11	J18	J13	J95	J45	J15	J20	J04	335	971	77	1,601	81.3	25.7	0.209	P

NORWAY

SARI-SARS-CoV-2		ICD 1	ICD 2	ICD 3	ICD 4	ICD 5	ICD 6	ICD 7	ICD 8	ICD 9	ICD 10	TP	FP	FN	TN	Se (%)	PPV(%)	CUI	
All ages	1	U071										33,538	435	4,380	138,240	88.4	98.7	0.873	E
	2	U071	J12									33,730	6,808	4,188	131,867	89.0	83.2	0.740	G
	3	U071	J12	J06								33,847	13,045	4,071	125,630	89.3	72.2	0.644	G
	4	U071	J12	J06	J00							33,857	13,647	4,061	125,028	89.3	71.3	0.636	S
	5	U071	J12	J06	J00	J22						33,953	16,817	3,965	121,858	89.5	66.9	0.599	S
	6	U071	J12	J06	J00	J22	J15					35,427	55,955	2,491	82,720	93.4	38.8	0.362	P
	7	U071	J12	J06	J00	J22	J15	J80				35,432	56,072	2,486	82,603	93.4	38.7	0.362	P
	8	U071	J12	J06	J00	J22	J15	J80	J-non			37,243	115,459	675	23,216	98.2	24.4	0.240	P
	9	U071	J12	J06	J00	J22	J15	J80	J-non	U072		37,321	115,537	597	23,138	98.4	24.4	0.240	P
	10	U071	J12	J06	J00	J22	J15	J80	J-non	U072	J02	37,336	116,141	582	22,534	98.5	24.3	0.240	P
High SARI	1	U071										19,221	230	2,505	55,100	88.5	98.8	0.874	E
	2	U071	J12									19,328	3,634	2,398	51,696	89.0	84.2	0.749	G
	3	U071	J12	J06								19,395	5,980	2,331	49,350	89.3	76.4	0.682	G
	4	U071	J12	J06	J00							19,400	6,210	2,326	49,120	89.3	75.8	0.676	G
	5	U071	J12	J06	J00	J80						19,408	6,281	2,318	49,049	89.3	75.5	0.675	G
	6	U071	J12	J06	J00	J80	J-non					20,772	34,686	954	20,644	95.6	37.5	0.358	P
	7	U071	J12	J06	J00	J80	J-non	J15				21,285	43,095	441	12,235	98.0	33.1	0.324	P
	8	U071	J12	J06	J00	J80	J-non	J15	J22			21,323	43,951	403	11,379	98.1	32.7	0.321	P
	9	U071	J12	J06	J00	J80	J-non	J15	J22	U072		21,365	43,977	361	11,353	98.3	32.7	0.322	P
	10	U071	J12	J06	J00	J80	J-non	J15	J22	U072	J02	21,372	44,179	354	11,151	98.4	32.6	0.321	P
Low SARI	1	U071										14,317	205	1,875	83,140	88.4	98.6	0.872	E
	2	U071	J12									14,402	3,174	1,790	80,171	88.9	81.9	0.729	G
	3	U071	J12	J06								14,452	7,065	1,740	76,280	89.3	67.2	0.599	S
	4	U071	J12	J06	J00							14,457	7,437	1,735	75,908	89.3	66.0	0.590	S
	5	U071	J12	J06	J00	J22						14,501	9,384	1,691	73,961	89.6	60.7	0.544	S
	6	U071	J12	J06	J00	J22	J80					14,507	9,560	1,685	73,785	89.6	60.3	0.540	S
	7	U071	J12	J06	J00	J22	J80	J-non				15,562	57,654	630	25,691	96.1	21.3	0.204	P
	8	U071	J12	J06	J00	J22	J80	J-non	J15			15,920	71,508	272	11,837	98.3	18.2	0.179	P
	9	U071	J12	J06	J00	J22	J80	J-non	J15	U072		15,956	71,560	236	11,785	98.5	18.2	0.180	P
	10	U071	J12	J06	J00	J22	J80	J-non	J15	U072	J02	15,964	71,962	228	11,383	98.6	18.2	0.179	P
0-4	1	U071										1,165	17	183	15,280	86.4	98.6	0.852	E
	2	U071	J06									1,214	3,513	134	11,784	90.1	25.7	0.231	P
	3	U071	J06	J04								1,227	3,898	121	11,399	91.0	23.9	0.218	P
	4	U071	J06	J04	J00							1,231	4,171	117	11,126	91.3	22.8	0.208	P
	5	U071	J06	J04	J00	J05						1,234	4,360	114	10,937	91.5	22.1	0.202	P
	6	U071	J06	J04	J00	J05	J-non					1,274	7,726	74	7,571	94.5	14.2	0.134	P
	7	U071	J06	J04	J00	J05	J-non	U072				1,277	7,735	71	7,562	94.7	14.2	0.134	P
	8	U071	J06	J04	J00	J05	J-non	U072	J12			1,291	8,863	57	6,434	95.8	12.7	0.122	P
	9	U071	J06	J04	J00	J05	J-non	U072	J12	J22		1,294	9,378	54	5,919	96.0	12.1	0.116	P

	10	U071	J06	J04	J00	J05	J-non	U072	J12	J22	J03	1,304	9,725	44	5,572	96.7	11.8	0.114	P
5-14	1	U071										343	7	63	3,102	84.5	98.0	0.828	E
	2	U071	J12									347	223	59	2,886	85.5	60.9	0.520	S
	3	U071	J12	U072								353	229	53	2,880	86.9	60.7	0.527	S
	4	U071	J12	U072	J06							359	780	47	2,329	88.4	31.5	0.279	P
	5	U071	J12	U072	J06	J22						362	924	44	2,185	89.2	28.1	0.251	P
	6	U071	J12	U072	J06	J22	J01					368	1,007	38	2,102	90.6	26.8	0.243	P
	7	U071	J12	U072	J06	J22	J01	J00				368	1,046	38	2,063	90.6	26.0	0.236	P
	8	U071	J12	U072	J06	J22	J01	J00	J-non			395	1,924	11	1,185	97.3	17.0	0.166	P
	9	U071	J12	U072	J06	J22	J01	J00	J-non	J17		395	1,925	11	1,184	97.3	17.0	0.166	P
	10	U071	J12	U072	J06	J22	J01	J00	J-non	J17	J10	400	2,346	6	763	98.5	14.6	0.144	P
15-64	1	U071										10,103	158	986	33,658	91.1	98.5	0.897	E
	2	U071	J12									10,152	1,469	937	32,347	91.6	87.4	0.800	G
	3	U071	J12	J00								10,154	1,652	935	32,164	91.6	86.0	0.788	G
	4	U071	J12	J00	J06							10,178	2,923	911	30,893	91.8	77.7	0.713	G
	5	U071	J12	J00	J06	J-non						10,678	20,601	411	13,215	96.3	34.1	0.329	P
	6	U071	J12	J00	J06	J-non	J80					10,680	20,619	409	13,197	96.3	34.1	0.329	P
	7	U071	J12	J00	J06	J-non	J80	J15				10,852	26,227	237	7,589	97.9	29.3	0.286	P
	8	U071	J12	J00	J06	J-non	J80	J15	J22			10,869	26,746	220	7,070	98.0	28.9	0.283	P
	9	U071	J12	J00	J06	J-non	J80	J15	J22	J02		10,882	27,124	207	6,692	98.1	28.6	0.281	P
	10	U071	J12	J00	J06	J-non	J80	J15	J22	J02	U072	10,912	27,171	177	6,645	98.4	28.7	0.282	P
65+	1	U071										21,927	253	3,148	86,200	87.4	98.9	0.864	E
	2	U071	J12									22,049	3,433	3,026	83,020	87.9	86.5	0.761	G
	3	U071	J12	J00								22,052	3,543	3,023	82,910	87.9	86.2	0.758	G
	4	U071	J12	J00	J06							22,090	4,490	2,985	81,963	88.1	83.1	0.732	G
	5	U071	J12	J00	J06	J22						22,154	6,067	2,921	80,386	88.4	78.5	0.694	G
	6	U071	J12	J00	J06	J22	J15					23,361	35,349	1,714	51,104	93.2	39.8	0.371	P
	7	U071	J12	J00	J06	J22	J15	U072				23,413	35,373	1,662	51,080	93.4	39.8	0.372	P
	8	U071	J12	J00	J06	J22	J15	U072	J-non			24,746	77,454	329	8,999	98.7	24.2	0.239	P
	9	U071	J12	J00	J06	J22	J15	U072	J-non	J80		24,746	77,463	329	8,990	98.7	24.2	0.239	P
	10	U071	J12	J00	J06	J22	J15	U072	J-non	J80	J02	24,747	77,532	328	8,921	98.7	24.2	0.239	P

NORWAY

SARI-Influenza		ICD 1	ICD 2	ICD 3	ICD 4	ICD 5	ICD 6	ICD 7	ICD 8	ICD 9	ICD 10	TP	FP	FN	TN	Se (%)	PPV(%)	CUI	
All ages	1	J10										7,430	210	1,478	150,236	83.4	97.3	0.811	E
	2	J10	J11									7,913	280	995	150,166	88.8	96.6	0.858	E
	3	J10	J11	J09								8,157	292	751	150,154	91.6	96.5	0.884	E
	4	J10	J11	J09	J-non							8,552	79,750	356	70,696	96.0	9.7	0.093	P
	5	J10	J11	J09	J-non	J15						8,668	103,800	240	46,646	97.3	7.7	0.075	P
	6	J10	J11	J09	J-non	J15	J13					8,679	105,000	229	45,446	97.4	7.6	0.074	P
	7	J10	J11	J09	J-non	J15	J13	J14				8,692	105,793	216	44,653	97.6	7.6	0.074	P
	8	J10	J11	J09	J-non	J15	J13	J14	J01			8,718	106,602	190	43,844	97.9	7.6	0.074	P
	9	J10	J11	J09	J-non	J15	J13	J14	J01	J18		8,761	113,222	147	37,224	98.3	7.2	0.071	P
	10	J10	J11	J09	J-non	J15	J13	J14	J01	J18	J04	8,770	113,870	138	36,576	98.5	7.2	0.070	P
High SARI	1	J10										5,173	93	1,007	64,875	83.7	98.2	0.822	E
	2	J10	J11									5,534	129	646	64,839	89.5	97.7	0.875	E
	3	J10	J11	J09								5,709	138	471	64,830	92.4	97.6	0.902	E
	4	J10	J11	J09	J15							5,846	17,556	334	47,412	94.6	25.0	0.236	P
	5	J10	J11	J09	J15	J-non						6,013	41,932	167	23,036	97.3	12.5	0.122	P
	6	J10	J11	J09	J15	J-non	J13					6,016	42,454	164	22,514	97.3	12.4	0.121	P
	7	J10	J11	J09	J15	J-non	J13	J14				6,025	42,837	155	22,131	97.5	12.3	0.120	P
	8	J10	J11	J09	J15	J-non	J13	J14	J01			6,045	43,197	135	21,771	97.8	12.3	0.120	P
	9	J10	J11	J09	J15	J-non	J13	J14	J01	J18		6,078	45,836	102	19,132	98.3	11.7	0.115	P
	10	J10	J11	J09	J15	J-non	J13	J14	J01	J18	J04	6,085	46,113	95	18,855	98.5	11.7	0.115	P
Low SARI	1	J10										2,257	117	471	85,361	82.7	95.1	0.787	G
	2	J10	J09									2,326	120	402	85,358	85.3	95.1	0.811	E
	3	J10	J09	J11								2,448	154	280	85,324	89.7	94.1	0.844	E
	4	J10	J09	J11	J-non							2,606	47,754	122	37,724	95.5	5.2	0.049	P
	5	J10	J09	J11	J-non	J15						2,655	61,868	73	23,610	97.3	4.1	0.040	P

	6	J10	J09	J11	J-non	J15	J13					2,663	62,546	65	22,932	97.6	4.1	0.040	P
	7	J10	J09	J11	J-non	J15	J13	J01				2,669	62,996	59	22,482	97.8	4.1	0.040	P
	8	J10	J09	J11	J-non	J15	J13	J01	J14			2,673	63,405	55	22,073	98.0	4.0	0.040	P
	9	J10	J09	J11	J-non	J15	J13	J01	J14	J18		2,683	67,386	45	18,092	98.4	3.8	0.038	P
	10	J10	J09	J11	J-non	J15	J13	J01	J14	J18	J04	2,685	67,757	43	17,721	98.4	3.8	0.038	P
**0-4**	1	J10										608	71	127	15,402	82.7	89.5	0.741	G
	2	J10	J09									624	71	111	15,402	84.9	89.8	0.762	G
	3	J10	J09	J11								647	78	88	15,395	88.0	89.2	0.786	G
	4	J10	J09	J11	J15							650	777	85	14,696	88.4	45.6	0.403	P
	5	J10	J09	J11	J15	J-non						673	4,251	62	11,222	91.6	13.7	0.125	P
	6	J10	J09	J11	J15	J-non	J06					693	7,589	42	7,884	94.3	8.4	0.079	P
	7	J10	J09	J11	J15	J-non	J06	J04				696	8,014	39	7,459	94.7	8.0	0.076	P
	8	J10	J09	J11	J15	J-non	J06	J04	J12			700	9,084	35	6,389	95.2	7.2	0.068	P
	9	J10	J09	J11	J15	J-non	J06	J04	J12	J05		702	9,279	33	6,194	95.5	7.0	0.067	P
	10	J10	J09	J11	J15	J-non	J06	J04	J12	J05	J01	707	9,302	28	6,171	96.2	7.1	0.068	P
**5-14**	1	J10										486	15	101	2,645	82.8	97.0	0.803	G
	2	J10	J11									526	17	61	2,643	89.6	96.9	0.868	E
	3	J10	J11	J09								536	17	51	2,643	91.3	96.9	0.885	E
	4	J10	J11	J09	J01							542	94	45	2,566	92.3	85.2	0.787	G
	5	J10	J11	J09	J01	J-non						556	1,099	31	1,561	94.7	33.6	0.318	P
	6	J10	J11	J09	J01	J-non	J15					559	1,294	28	1,366	95.2	30.2	0.287	P
	7	J10	J11	J09	J01	J-non	J15	J18				561	1,340	26	1,320	95.6	29.5	0.282	P
	8	J10	J11	J09	J01	J-non	J15	J18	J02			563	1,373	24	1,287	95.9	29.1	0.279	P
	9	J10	J11	J09	J01	J-non	J15	J18	J02	J06		575	1,795	12	865	98.0	24.3	0.238	P
	10	J10	J11	J09	J01	J-non	J15	J18	J02	J06	J03	578	1,940	9	720	98.5	23.0	0.226	P
**15-64**	1	J10										2,564	54	522	35,335	83.1	97.9	0.814	E
	2	J10	J11									2,753	92	333	35,297	89.2	96.8	0.863	E
	3	J10	J11	J09								2,856	98	230	35,291	92.5	96.7	0.895	E
	4	J10	J11	J09	J-non							2,951	17,013	135	18,376	95.6	14.8	0.141	P
	5	J10	J11	J09	J-non	J15						2,994	22,760	92	12,629	97.0	11.6	0.113	P
	6	J10	J11	J09	J-non	J15	J13					3,002	23,189	84	12,200	97.3	11.5	0.111	P
	7	J10	J11	J09	J-non	J15	J13	J01				3,017	23,738	69	11,651	97.8	11.3	0.110	P
	8	J10	J11	J09	J-non	J15	J13	J01	J14			3,021	23,983	65	11,406	97.9	11.2	0.110	P
	9	J10	J11	J09	J-non	J15	J13	J01	J14	J18		3,035	25,578	51	9,811	98.3	10.6	0.104	P
	10	J10	J11	J09	J-non	J15	J13	J01	J14	J18	J04	3,039	25,646	47	9,743	98.5	10.6	0.104	P
**65+**	1	J10										3,772	70	728	96,854	83.8	98.2	0.823	E
	2	J10	J11									4,003	93	497	96,831	89.0	97.7	0.869	E
	3	J10	J11	J09								4,118	99	382	96,825	91.5	97.7	0.894	E
	4	J10	J11	J09	J-non							4,381	57,967	119	38,957	97.4	7.0	0.068	P
	5	J10	J11	J09	J-non	J15						4,448	75,571	52	21,353	98.8	5.6	0.055	P
	6	J10	J11	J09	J-non	J15	J14					4,457	76,097	43	20,827	99.0	5.5	0.055	P
	7	J10	J11	J09	J-non	J15	J14	J13				4,460	76,825	40	20,099	99.1	5.5	0.054	P
	8	J10	J11	J09	J-non	J15	J14	J13	J18			4,481	81,650	19	15,274	99.6	5.2	0.052	P
	9	J10	J11	J09	J-non	J15	J14	J13	J18	U071		4,486	92,445	14	4,479	99.7	4.6	0.046	P
	10	J10	J11	J09	J-non	J15	J14	J13	J18	U071	J16	4,486	92,471	14	4,453	99.7	4.6	0.046	P

NORWAY

SARI-RSV		ICD 1	ICD 2	ICD 3	ICD 4	ICD 5	ICD 6	ICD 7	ICD 8	ICD 9	ICD 10	TP	FP	FN	TN	Se (%)	PPV(%)	CUI	
**All ages**	1	J21										3,233	1,317	5,220	103,032	38.2	71.1	0.272	P
	2	J21	J12									5,758	10,112	2,695	94,237	68.1	36.3	0.247	P
	3	J21	J12	J20								6,844	13,565	1,609	90,784	81.0	33.5	0.272	P
	4	J21	J12	J20	J06							7,225	19,545	1,228	84,804	85.5	27.0	0.231	P
	5	J21	J12	J20	J06	J_non						7,907	69,329	546	35,020	93.5	10.2	0.096	P
	6	J21	J12	J20	J06	J_non	J13					7,928	70,057	525	34,292	93.8	10.2	0.095	P
	7	J21	J12	J20	J06	J_non	J13	J15				8,098	84,862	355	19,487	95.8	8.7	0.083	P
	8	J21	J12	J20	J06	J_non	J13	J15	J22			8,196	86,632	257	17,717	97.0	8.6	0.084	P
	9	J21	J12	J20	J06	J_non	J13	J15	J22	J00		8,241	87,356	212	16,993	97.5	8.6	0.084	P
	10	J21	J12	J20	J06	J_non	J13	J15	J22	J00	J01	8,269	87,901	184	16,448	97.8	8.6	0.084	P
**High SARI**	1	J21										2,547	560	3,784	43,648	40.2	82.0	0.330	P
	2	J21	J12									4,394	4,897	1,937	39,311	69.4	47.3	0.328	P
	3	J21	J12	J20								5,241	6,080	1,090	38,128	82.8	46.3	0.383	P
	4	J21	J12	J20	J06							5,497	8,446	834	35,762	86.8	39.4	0.342	P

	5	J21	J12	J20	J06	J_non						5,961	28,158	370	16,050	94.2	17.5	0.165	P
	6	J21	J12	J20	J06	J_non	J13					5,974	28,483	357	15,725	94.4	17.3	0.164	P
	7	J21	J12	J20	J06	J_non	J13	J22				6,053	29,221	278	14,987	95.6	17.2	0.164	P
	8	J21	J12	J20	J06	J_non	J13	J22	J15			6,158	35,175	173	9,033	97.3	14.9	0.145	P
	9	J21	J12	J20	J06	J_non	J13	J22	J15	J01		6,182	35,421	149	8,787	97.6	14.9	0.145	P
	10	J21	J12	J20	J06	J_non	J13	J22	J15	J01	J00	6,214	35,755	117	8,453	98.2	14.8	0.145	P
**Low SARI**	1	J12										694	4,483	1,428	55,658	32.7	13.4	0.044	P
	2	J12	J21									1,364	5,215	758	54,926	64.3	20.7	0.133	P
	3	J12	J21	J20								1,603	7,485	519	52,656	75.5	17.6	0.133	P
	4	J12	J21	J20	J_non							1,836	38,007	286	22,134	86.5	4.6	0.040	P
	5	J12	J21	J20	J_non	J06						1,946	41,171	176	18,970	91.7	4.5	0.041	P
	6	J12	J21	J20	J_non	J06	J13					1,954	41,574	168	18,567	92.1	4.5	0.041	P
	7	J12	J21	J20	J_non	J06	J13	J04				1,964	41,884	158	18,257	92.6	4.5	0.041	P
	8	J12	J21	J20	J_non	J06	J13	J04	J22			1,987	42,964	135	17,177	93.6	4.4	0.041	P
	9	J12	J21	J20	J_non	J06	J13	J04	J22	J15		2,048	51,764	74	8,377	96.5	3.8	0.037	P
	10	J12	J21	J20	J_non	J06	J13	J04	J22	J15	J00	2,061	52,151	61	7,990	97.1	3.8	0.037	P
**0-4**	1	J21										3,098	1,127	1,488	9,544	67.6	73.3	0.495	S
	2	J21	J12									3,659	2,083	927	8,588	79.8	63.7	0.508	S
	3	J21	J12	J_non								3,836	4,169	750	6,502	83.6	47.9	0.401	P
	4	J21	J12	J_non	J20							4,181	5,021	405	5,650	91.2	45.4	0.414	P
	5	J21	J12	J_non	J20	J15						4,213	5,338	373	5,333	91.9	44.1	0.405	P
	6	J21	J12	J_non	J20	J15	J18					4,231	5,448	355	5,223	92.3	43.7	0.403	P
	7	J21	J12	J_non	J20	J15	J18	J06				4,452	8,250	134	2,421	97.1	35.0	0.340	P
	8	J21	J12	J_non	J20	J15	J18	J06	J22			4,503	8,657	83	2,014	98.2	34.2	0.336	P
	9	J21	J12	J_non	J20	J15	J18	J06	J22	J10		4,518	9,108	68	1,563	98.5	33.2	0.327	P
	10	J21	J12	J_non	J20	J15	J18	J06	J22	J10	J13	4,519	9,124	67	1,547	98.5	33.1	0.326	P
**5-14**	1	J12										56	159	144	2,559	28.0	26.0	0.073	P
	2	J12	J21									88	177	112	2,541	44.0	33.2	0.146	P
	3	J12	J21	J20								122	385	78	2,333	61.0	24.1	0.147	P
	4	J12	J21	J20	J_non							143	1,149	57	1,569	71.5	11.1	0.079	P
	5	J12	J21	J20	J_non	J06						175	1,533	25	1,185	87.5	10.2	0.090	P
	6	J12	J21	J20	J_non	J06	J04					179	1,602	21	1,116	89.5	10.1	0.090	P
	7	J12	J21	J20	J_non	J06	J04	J15				184	1,772	16	946	92.0	9.4	0.087	P
	8	J12	J21	J20	J_non	J06	J04	J15	J05			187	1,790	13	928	93.5	9.5	0.088	P
	9	J12	J21	J20	J_non	J06	J04	J15	J05	J18		188	1,831	12	887	94.0	9.3	0.088	P
	10	J12	J21	J20	J_non	J06	J04	J15	J05	J18	J01	190	1,895	10	823	95.0	9.1	0.087	P
**15-64**	1	J12										456	2,147	563	23,728	44.7	17.5	0.078	P
	2	J12	J20									668	2,993	351	22,882	65.6	18.2	0.120	P
	3	J12	J20	J21								695	3,068	324	22,807	68.2	18.5	0.126	P
	4	J12	J20	J21	J_non							842	14,285	177	11,590	82.6	5.6	0.046	P
	5	J12	J20	J21	J_non	J06						892	15,304	127	10,571	87.5	5.5	0.048	P
	6	J12	J20	J21	J_non	J06	J13					898	15,579	121	10,296	88.1	5.5	0.048	P
	7	J12	J20	J21	J_non	J06	J13	J01				918	15,965	101	9,910	90.1	5.4	0.049	P
	8	J12	J20	J21	J_non	J06	J13	J01	J00			934	16,184	85	9,691	91.7	5.5	0.050	P
	9	J12	J20	J21	J_non	J06	J13	J01	J00	J15		972	19,931	47	5,944	95.4	4.7	0.044	P
	10	J12	J20	J21	J_non	J06	J13	J01	J00	J15	J18	984	21,009	35	4,866	96.6	4.5	0.043	P
**65+**	1	J12										1,464	5,538	1,184	59,547	55.3	20.9	0.116	P
	2	J12	J20									1,902	6,846	746	58,239	71.8	21.7	0.156	P
	3	J12	J20	J21								1,961	6,935	687	58,150	74.1	22.0	0.163	P
	4	J12	J20	J21	J13							1,989	7,829	659	57,256	75.1	20.3	0.152	P
	5	J12	J20	J21	J13	J_non						2,401	44,010	247	21,075	90.7	5.2	0.047	P
	6	J12	J20	J21	J13	J_non	J15					2,499	54,606	149	10,479	94.4	4.4	0.041	P
	7	J12	J20	J21	J13	J_non	J15	J06				2,546	55,637	102	9,448	96.1	4.4	0.042	P
	8	J12	J20	J21	J13	J_non	J15	J06	J14			2,550	55,931	98	9,154	96.3	4.4	0.042	P
	9	J12	J20	J21	J13	J_non	J15	J06	J14	J18		2,586	58,998	62	6,087	97.7	4.2	0.041	P
	10	J12	J20	J21	J13	J_non	J15	J06	J14	J18	J22	2,619	59,897	29	5,188	98.9	4.2	0.041	P

NORWAY

SARI-any of the three	ICD 1	ICD 2	ICD 3	ICD 4	ICD 5	ICD 6	ICD 7	ICD 8	ICD 9	ICD 10	TP	FP	FN	TN	Se (%)	PPV(%)	CUI

**All ages**	1	U071										17,968	243	16,333	77,143	52.4	98.7	0.517	S
	2	U071	J10									22,615	395	11,686	76,991	65.9	98.3	0.648	G
	3	U071	J10	J12								25,257	3,584	9,044	73,802	73.6	87.6	0.645	G
	4	U071	J10	J12	J21							28,331	4,756	5,970	72,630	82.6	85.6	0.707	G
	5	U071	J10	J12	J21	J11						28,635	4,807	5,666	72,579	83.5	85.6	0.715	G
	6	U071	J10	J12	J21	J11	J06					29,164	9,441	5,137	67,945	85.0	75.5	0.642	G
	7	U071	J10	J12	J21	J11	J06	J20				30,243	12,542	4,058	64,844	88.2	70.7	0.623	S
	8	U071	J10	J12	J21	J11	J06	J20	J00			30,306	12,954	3,995	64,432	88.4	70.1	0.619	S
	9	U071	J10	J12	J21	J11	J06	J20	J00	J09		30,429	12,958	3,872	64,428	88.7	70.1	0.622	S
	10	U071	J10	J12	J21	J11	J06	J20	J00	J09	J_non	32,820	56,123	1,481	21,263	95.7	36.9	0.353	P
**High SARI**	1	U071										10,383	116	11,311	28,286	47.9	98.9	0.473	P
	2	U071	J10									13,553	177	8,141	28,225	62.5	98.7	0.617	S
	3	U071	J10	J12								15,455	1,387	6,239	27,015	71.2	91.8	0.654	G
	4	U071	J10	J12	J21							17,866	1,879	3,828	26,523	82.4	90.5	0.745	G
	5	U071	J10	J12	J21	J11						18,087	1,904	3,607	26,498	83.4	90.5	0.754	G
	6	U071	J10	J12	J21	J11	J06					18,429	3,507	3,265	24,895	84.9	84.0	0.714	G
	7	U071	J10	J12	J21	J11	J06	J20				19,265	4,510	2,429	23,892	88.8	81.0	0.720	G
	8	U071	J10	J12	J21	J11	J06	J20	J00			19,307	4,648	2,387	23,754	89.0	80.6	0.717	G
	9	U071	J10	J12	J21	J11	J06	J20	J00	J09		19,389	4,649	2,305	23,753	89.4	80.7	0.721	G
	10	U071	J10	J12	J21	J11	J06	J20	J00	J09	J_non	20,821	20,485	873	7,917	96.0	50.4	0.484	P
**Low SARI**	1	U071										7,585	127	5,022	48,857	60.2	98.4	0.592	S
	2	U071	J12									8,348	2,106	4,259	46,878	66.2	79.9	0.529	S
	3	U071	J12	J10								9,802	2,197	2,805	46,787	77.8	81.7	0.635	S
	4	U071	J12	J10	J21							10,465	2,877	2,142	46,107	83.0	78.4	0.651	G
	5	U071	J12	J10	J21	J06						10,652	5,908	1,955	43,076	84.5	64.3	0.543	S
	6	U071	J12	J10	J21	J06	J11					10,735	5,934	1,872	43,050	85.2	64.4	0.548	S
	7	U071	J12	J10	J21	J06	J11	J00				10,756	6,213	1,851	42,771	85.3	63.4	0.541	S
	8	U071	J12	J10	J21	J06	J11	J00	J80			10,763	6,341	1,844	42,643	85.4	62.9	0.537	S
	9	U071	J12	J10	J21	J06	J11	J00	J80	J22		10,828	7,659	1,779	41,325	85.9	58.6	0.503	S
	10	U071	J12	J10	J21	J06	J11	J00	J80	J22	J09	10,869	7,662	1,738	41,322	86.2	58.7	0.506	S
**0-4**	1	U071										1,014	9	5,189	8,882	16.3	99.1	0.162	P
	2	U071	J21									4,070	1,041	2,133	7,850	65.6	79.6	0.522	S
	3	U071	J21	J10								4,600	1,093	1,603	7,798	74.2	80.8	0.599	S
	4	U071	J21	J10	J_non							4,949	3,254	1,254	5,637	79.8	60.3	0.481	P
	5	U071	J21	J10	J_non	J12						5,358	3,883	845	5,008	86.4	58.0	0.501	S
	6	U071	J21	J10	J_non	J12	J20					5,694	4,699	509	4,192	91.8	54.8	0.503	S
	7	U071	J21	J10	J_non	J12	J20	J09				5,703	4,699	500	4,192	91.9	54.8	0.504	S
	8	U071	J21	J10	J_non	J12	J20	J09	J11			5,722	4,706	481	4,185	92.2	54.9	0.506	S
	9	U071	J21	J10	J_non	J12	J20	J09	J11	J15		5,755	4,995	448	3,896	92.8	53.5	0.497	S
	10	U071	J21	J10	J_non	J12	J20	J09	J11	J15	J06	6,028	7,427	175	1,464	97.2	44.8	0.435	P
**5-14**	1	J10										416	11	531	1,933	43.9	97.4	0.428	P
	2	J10	U071									639	16	308	1,928	67.5	97.6	0.658	G
	3	J10	U071	J11								672	18	275	1,926	71.0	97.4	0.691	G
	4	J10	U071	J11	J12							726	165	221	1,779	76.7	81.5	0.625	S
	5	J10	U071	J11	J12	J21						758	183	189	1,761	80.0	80.6	0.645	G
	6	J10	U071	J11	J12	J21	J09					766	183	181	1,761	80.9	80.7	0.653	G
	7	J10	U071	J11	J12	J21	J09	J20				802	380	145	1,564	84.7	67.9	0.575	S
	8	J10	U071	J11	J12	J21	J09	J20	J01			814	434	133	1,510	86.0	65.2	0.561	S
	9	J10	U071	J11	J12	J21	J09	J20	J01	J06		861	867	86	1,077	90.9	49.8	0.453	P
	10	J10	U071	J11	J12	J21	J09	J20	J01	J06	J80	861	867	86	1,077	90.9	49.8	0.453	P
**15-64**	1	U071										4,704	80	3,404	18,437	58.0	98.3	0.570	S
	2	U071	J10									6,269	119	1,839	18,398	77.3	98.1	0.759	G
	3	U071	J10	J12								6,747	846	1,361	17,671	83.2	88.9	0.739	G
	4	U071	J10	J12	J11							6,858	876	1,250	17,641	84.6	88.7	0.750	G
	5	U071	J10	J12	J11	J00						6,882	980	1,226	17,537	84.9	87.5	0.743	G
	6	U071	J10	J12	J11	J00	J06					6,973	1,799	1,135	16,718	86.0	79.5	0.684	G
	7	U071	J10	J12	J11	J00	J06	J_non				7,554	11,795	554	6,722	93.2	39.0	0.364	P
	8	U071	J10	J12	J11	J00	J06	J_non	J20			7,702	12,270	406	6,247	95.0	38.6	0.366	P
	9	U071	J10	J12	J11	J00	J06	J_non	J20	J80		7,703	12,283	405	6,234	95.0	38.5	0.366	P
	10	U071	J10	J12	J11	J00	J06	J_non	J20	J80	J09	7,749	12,285	359	6,232	95.6	38.7	0.370	P
**65 +**	1	U071										12,020	149	7,023	47,885	63.1	98.8	0.623	S

	2	U071	J10									14,135	199	4,908	47,835	74.2	98.6	0.732	G
	3	U071	J10	J12								15,617	1,600	3,426	46,434	82.0	90.7	0.744	G
	4	U071	J10	J12	J06							15,706	2,178	3,337	45,856	82.5	87.8	0.724	G
	5	U071	J10	J12	J06	J00						15,720	2,247	3,323	45,787	82.6	87.5	0.722	G
	6	U071	J10	J12	J06	J00	J11					15,861	2,259	3,182	45,775	83.3	87.5	0.729	G
	7	U071	J10	J12	J06	J00	J11	J20				16,302	3,400	2,741	44,634	85.6	82.7	0.708	G
	8	U071	J10	J12	J06	J00	J11	J20	J09			16,356	3,401	2,687	44,633	85.9	82.8	0.711	G
	9	U071	J10	J12	J06	J00	J11	J20	J09	J22		16,448	4,346	2,595	43,688	86.4	79.1	0.683	G
	10	U071	J10	J12	J06	J00	J11	J20	J09	J22	J80	16,459	4,436	2,584	43,598	86.4	78.8	0.681	G

SPAIN

SARI-SARS-CoV-2		ICD 1	ICD 2	ICD 3	ICD 4	ICD 5	ICD 6	ICD 7	ICD 8	ICD 9	ICD 10	TP	FP	FN	TN	Se (%)	PPV(%)	CUI	
All ages	1	U071										6,158	324	277	21,721	95.7	95.0	0.909	E
	2	U071	J12									6,169	603	266	21,442	95.9	91.1	0.873	E
	3	U071	J12	J80								6,169	633	266	21,412	95.9	90.7	0.869	E
	4	U071	J12	J80	J96							6,271	8,661	164	13,384	97.5	42.0	0.409	P
	5	U071	J12	J80	J96	J15						6,273	8,925	162	13,120	97.5	41.3	0.402	P
	6	U071	J12	J80	J96	J15	J84					6,277	9,151	158	12,894	97.5	40.7	0.397	P
	7	U071	J12	J80	J96	J15	J84	J98				6,304	11,059	131	10,986	98.0	36.3	0.356	P
	8	U071	J12	J80	J96	J15	J84	J98	J95			6,304	11,071	131	10,974	98.0	36.3	0.355	P
	9	U071	J12	J80	J96	J15	J84	J98	J95	J44		6,341	13,612	94	8,433	98.5	31.8	0.313	P
	10	U071	J12	J80	J96	J15	J84	J98	J95	J44	J22	6,357	14,250	78	7,795	98.8	30.8	0.305	P
High SARI	1	U071										3,441	183	126	10,115	96.5	95.0	0.916	E
	2	U071	J12									3,443	379	124	9,919	96.5	90.1	0.870	E
	3	U071	J12	J80								3,443	390	124	9,908	96.5	89.8	0.867	E
	4	U071	J12	J80	J15							3,446	569	121	9,729	96.6	85.8	0.829	E
	5	U071	J12	J80	J15	J96						3,497	4,273	70	6,025	98.0	45.0	0.441	P
	6	U071	J12	J80	J15	J96	J84					3,498	4,385	69	5,913	98.1	44.4	0.435	P
	7	U071	J12	J80	J15	J96	J84	J95				3,498	4,392	69	5,906	98.1	44.3	0.435	P
	8	U071	J12	J80	J15	J96	J84	J95	J69			3,499	4,543	68	5,755	98.1	43.5	0.427	P
	9	U071	J12	J80	J15	J96	J84	J95	J69	J98		3,509	5,529	58	4,769	98.4	38.8	0.382	P
	10	U071	J12	J80	J15	J96	J84	J95	J69	J98	J92	3,510	5,549	57	4,749	98.4	38.7	0.381	P
Low SARI	1	U071										2,717	141	151	11,606	94.7	95.1	0.901	E
	2	U071	J12									2,726	224	142	11,523	95.0	92.4	0.878	E
	3	U071	J12	J96								2,775	4,494	93	7,253	96.8	38.2	0.369	P
	4	U071	J12	J96	J98							2,793	5,421	75	6,326	97.4	34.0	0.331	P
	5	U071	J12	J96	J98	J15						2,794	5,561	74	6,186	97.4	33.4	0.326	P
	6	U071	J12	J96	J98	J15	J80					2,794	5,571	74	6,176	97.4	33.4	0.325	P
	7	U071	J12	J96	J98	J15	J80	J84				2,796	5,673	72	6,074	97.5	33.0	0.322	P
	8	U071	J12	J96	J98	J15	J80	J84	J95			2,796	5,678	72	6,069	97.5	33.0	0.322	P
	9	U071	J12	J96	J98	J15	J80	J84	J95	J22		2,814	6,192	54	5,555	98.1	31.2	0.307	P
	10	U071	J12	J96	J98	J15	J80	J84	J95	J22	J94	2,815	6,203	53	5,544	98.2	31.2	0.306	P
0-4	1	U071										112	13	13	2,015	89.6	89.6	0.803	G
	2	U071	J21									121	910	4	1,118	96.8	11.7	0.114	P
	3	U071	J21	J04								121	955	4	1,073	96.8	11.2	0.109	P
	4	U071	J21	J04	J12							122	1,055	3	973	97.6	10.4	0.101	P
	5	U071	J21	J04	J12	J06						122	1,126	3	902	97.6	9.8	0.095	P
	6	U071	J21	J04	J12	J06	J96					124	1,681	1	347	99.2	6.9	0.068	P
	7	U071	J21	J04	J12	J06	J96	J34				124	1,681	1	347	99.2	6.9	0.068	P
	8	U071	J21	J04	J12	J06	J96	J34	J98			125	1,737	0	291	100.0	6.7	0.067	P
	9	U071	J21	J04	J12	J06	J96	J34	J98	J20		125	1,823	0	205	100.0	6.4	0.064	P
	10	U071	J21	J04	J12	J06	J96	J34	J98	J20	J45	125	1,863	0	165	100.0	6.3	0.063	P
5-14	1	U071										20	8	4	594	83.3	71.4	0.595	S
	2	U071	J86									20	8	4	594	83.3	71.4	0.595	S
	3	U071	J86	J12								22	29	2	573	91.7	43.1	0.395	P
	4	U071	J86	J12	J95							22	29	2	573	91.7	43.1	0.395	P
	5	U071	J86	J12	J95	J99						22	29	2	573	91.7	43.1	0.395	P
	6	U071	J86	J12	J95	J99	J13					22	37	2	565	91.7	37.3	0.342	P
	7	U071	J86	J12	J95	J99	J13	J84				22	38	2	564	91.7	36.7	0.336	P
	8	U071	J86	J12	J95	J99	J13	J84	J15			22	44	2	558	91.7	33.3	0.306	P
	9	U071	J86	J12	J95	J99	J13	J84	J15	J04		22	51	2	551	91.7	30.1	0.276	P

	10	U071	J86	J12	J95	J99	J13	J84	J15	J04	J98	23	207	1	395	95.8	10.0	0.096	P
15-64	1	U071										1,208	90	39	4,832	96.9	93.1	0.902	E
	2	U071	J12									1,210	128	37	4,794	97.0	90.4	0.878	E
	3	U071	J12	J80								1,210	143	37	4,779	97.0	89.4	0.868	E
	4	U071	J12	J80	J96							1,221	1,441	26	3,481	97.9	45.9	0.449	P
	5	U071	J12	J80	J96	J84						1,221	1,482	26	3,440	97.9	45.2	0.442	P
	6	U071	J12	J80	J96	J84	J98					1,225	1,890	22	3,032	98.2	39.3	0.386	P
	7	U071	J12	J80	J96	J84	J98	J95				1,225	1,891	22	3,031	98.2	39.3	0.386	P
	8	U071	J12	J80	J96	J84	J98	J95	J15			1,225	1,959	22	2,963	98.2	38.5	0.378	P
	9	U071	J12	J80	J96	J84	J98	J95	J15	J22		1,226	2,187	21	2,735	98.3	35.9	0.353	P
	10	U071	J12	J80	J96	J84	J98	J95	J15	J22	J91	1,227	2,221	20	2,701	98.4	35.6	0.350	P
65+	1	U071										4,818	213	221	14,280	95.6	95.8	0.916	E
	2	U071	J12									4,824	323	215	14,170	95.7	93.7	0.897	E
	3	U071	J12	J15								4,828	608	211	13,885	95.8	88.8	0.851	E
	4	U071	J12	J15	J80							4,828	621	211	13,872	95.8	88.6	0.849	E
	5	U071	J12	J15	J80	J96						4,909	5,863	130	8,630	97.4	45.6	0.444	P
	6	U071	J12	J15	J80	J96	J95					4,909	5,874	130	8,619	97.4	45.5	0.444	P
	7	U071	J12	J15	J80	J96	J95	J98				4,932	7,284	107	7,209	97.9	40.4	0.395	P
	8	U071	J12	J15	J80	J96	J95	J98	J84			4,935	7,443	104	7,050	97.9	39.9	0.390	P
	9	U071	J12	J15	J80	J96	J95	J98	J84	J93		4,935	7,446	104	7,047	97.9	39.9	0.390	P
	10	U071	J12	J15	J80	J96	J95	J98	J84	J93	J69	4,939	7,761	100	6,732	98.0	38.9	0.381	P

SPAIN

SARI-Influenza		ICD 1	ICD 2	ICD 3	ICD 4	ICD 5	ICD 6	ICD 7	ICD 8	ICD 9	ICD 10	TP	FP	FN	TN	Se (%)	PPV(%)	CUI	
All ages	1	J10										1,441	27	236	8,734	85.9	98.2	0.843	E
	2	J10	J09									1,572	27	105	8,734	93.7	98.3	0.922	E
	3	J10	J09	J98								1,593	1,267	84	7,494	95.0	55.7	0.529	S
	4	J10	J09	J98	J11							1,614	1,267	63	7,494	96.2	56.0	0.539	S
	5	J10	J09	J98	J11	J45						1,621	2,137	56	6,624	96.7	43.1	0.417	P
	6	J10	J09	J98	J11	J45	J44					1,632	3,482	45	5,279	97.3	31.9	0.311	P
	7	J10	J09	J98	J11	J45	J44	J15				1,633	3,633	44	5,128	97.4	31.0	0.302	P
	8	J10	J09	J98	J11	J45	J44	J15	J22			1,638	3,928	39	4,833	97.7	29.4	0.287	P
	9	J10	J09	J98	J11	J45	J44	J15	J22	J96		1,648	5,478	29	3,283	98.3	23.1	0.227	P
	10	J10	J09	J98	J11	J45	J44	J15	J22	J96	J32	1,649	5,482	28	3,279	98.3	23.1	0.227	P
High SARI	1	J10										526	13	76	4,003	87.4	97.6	0.853	E
	2	J10	J09									570	13	32	4,003	94.7	97.8	0.926	E
	3	J10	J09	J98								578	685	24	3,331	96.0	45.8	0.439	P
	4	J10	J09	J98	J15							579	760	23	3,256	96.2	43.2	0.416	P
	5	J10	J09	J98	J15	J45						583	1,149	19	2,867	96.8	33.7	0.326	P
	6	J10	J09	J98	J15	J45	J11					584	1,149	18	2,867	97.0	33.7	0.327	P
	7	J10	J09	J98	J15	J45	J11	J13				587	1,404	15	2,612	97.5	29.5	0.287	P
	8	J10	J09	J98	J15	J45	J11	J13	J47			588	1,496	14	2,520	97.7	28.2	0.276	P
	9	J10	J09	J98	J15	J45	J11	J13	J47	J96		594	2,488	8	1,528	98.7	19.3	0.190	P
	10	J10	J09	J98	J15	J45	J11	J13	J47	J96	J44	596	2,772	6	1,244	99.0	17.7	0.175	P
Low SARI	1	J10										915	14	160	4,731	85.1	98.5	0.838	E
	2	J10	J09									1,002	14	73	4,731	93.2	98.6	0.919	E
	3	J10	J09	J11								1,026	14	49	4,731	95.4	98.7	0.942	E
	4	J10	J09	J11	J98							1,034	582	41	4,163	96.2	64.0	0.615	S
	5	J10	J09	J11	J98	J44						1,041	1,372	34	3,373	96.8	43.1	0.418	P
	6	J10	J09	J11	J98	J44	J45					1,045	1,800	30	2,945	97.2	36.7	0.357	P
	7	J10	J09	J11	J98	J44	J45	J22				1,047	1,933	28	2,812	97.4	35.1	0.342	P
	8	J10	J09	J11	J98	J44	J45	J22	J96			1,052	2,738	23	2,007	97.9	27.8	0.272	P
	9	J10	J09	J11	J98	J44	J45	J22	J96	J20		1,056	2,795	19	1,950	98.2	27.4	0.269	P
	10	J10	J09	J11	J98	J44	J45	J22	J96	J20	J30	1,056	2,803	19	1,942	98.2	27.4	0.269	P
0-4	1	J10										61	1	6	1,008	91.0	98.4	0.896	E
	2	J10	J96									66	651	1	358	98.5	9.2	0.091	P
	3	J10	J96	J98								66	687	1	322	98.5	8.8	0.086	P
	4	J10	J96	J98	J04							67	698	0	311	100.0	8.8	0.088	P
	5	J10	J96	J98	J04	J14						67	698	0	311	100.0	8.8	0.088	P
	6	J10	J96	J98	J04	J14	J03					67	700	0	309	100.0	8.7	0.087	P
	7	J10	J96	J98	J04	J14	J03	J18				67	794	0	215	100.0	7.8	0.078	P
	8	J10	J96	J98	J04	J14	J03	J18	J69			67	794	0	215	100.0	7.8	0.078	P

	9	J10	J96	J98	J04	J14	J03	J18	J69	J20		67	810	0	199	100.0	7.6	0.076	P
	10	J10	J96	J98	J04	J14	J03	J18	J69	J20	J21	67	899	0	110	100.0	6.9	0.069	P
5-14	1	J10										36	2	8	300	81.8	94.7	0.775	G
	2	J10	J91									37	12	7	290	84.1	75.5	0.635	S
	3	J10	J91	J90								38	21	6	281	86.4	64.4	0.556	S
	4	J10	J91	J90	J16							38	21	6	281	86.4	64.4	0.556	S
	5	J10	J91	J90	J16	J09						39	21	5	281	88.6	65.0	0.576	S
	6	J10	J91	J90	J16	J09	J96					42	164	2	138	95.5	20.4	0.195	P
	7	J10	J91	J90	J16	J09	J96	J45				42	216	2	86	95.5	16.3	0.155	P
	8	J10	J91	J90	J16	J09	J96	J45	J69			42	218	2	84	95.5	16.2	0.154	P
	9	J10	J91	J90	J16	J09	J96	J45	J69	J20		43	220	1	82	97.7	16.3	0.160	P
	10	J10	J91	J90	J16	J09	J96	J45	J69	J20	J13	43	222	1	80	97.7	16.2	0.159	P
15-64	1	J10										358	4	65	1,731	84.6	98.9	0.837	E
	2	J10	J09									389	4	34	1,731	92.0	99.0	0.910	E
	3	J10	J09	J11								402	4	21	1,731	95.0	99.0	0.941	E
	4	J10	J09	J11	J98							405	161	18	1,574	95.7	71.6	0.685	G
	5	J10	J09	J11	J98	J45						407	446	16	1,289	96.2	47.7	0.459	P
	6	J10	J09	J11	J98	J45	J44					411	657	12	1,078	97.2	38.5	0.374	P
	7	J10	J09	J11	J98	J45	J44	J15				411	688	12	1,047	97.2	37.4	0.363	P
	8	J10	J09	J11	J98	J45	J44	J15	J96			413	910	10	825	97.6	31.2	0.305	P
	9	J10	J09	J11	J98	J45	J44	J15	J96	J13		414	1,018	9	717	97.9	28.9	0.283	P
	10	J10	J09	J11	J98	J45	J44	J15	J96	J13	J22	415	1,050	8	685	98.1	28.3	0.278	P
65+	1	J10										986	20	157	5,695	86.3	98.0	0.845	E
	2	J10	J09									1,085	20	58	5,695	94.9	98.2	0.932	E
	3	J10	J09	J98								1,093	721	50	4,994	95.6	60.3	0.576	S
	4	J10	J09	J98	J45							1,098	1,188	45	4,527	96.1	48.0	0.461	P
	5	J10	J09	J98	J45	J96						1,109	2,722	34	2,993	97.0	28.9	0.281	P
	6	J10	J09	J98	J45	J96	J11					1,121	2,722	22	2,993	98.1	29.2	0.286	P
	7	J10	J09	J98	J45	J96	J11	J22				1,123	2,914	20	2,801	98.3	27.8	0.273	P
	8	J10	J09	J98	J45	J96	J11	J22	J44			1,127	3,522	16	2,193	98.6	24.2	0.239	P
	9	J10	J09	J98	J45	J96	J11	J22	J44	J20		1,129	3,598	14	2,117	98.8	23.9	0.236	P
	10	J10	J09	J98	J45	J96	J11	J22	J44	J20	J15	1,129	3,670	14	2,045	98.8	23.5	0.232	P

SPAIN

SARI-RSV		ICD 1	ICD 2	ICD 3	ICD 4	ICD 5	ICD 6	ICD 7	ICD 8	ICD 9	ICD 10	TP	FP	FN	TN	Se (%)	PPV(%)	CUI	
All ages	1	J21										139	99	141	1,242	49.6	58.4	0.290	P
	2	J21	J96									249	738	31	603	88.9	25.2	0.224	P
	3	J21	J96	J98								260	804	20	537	92.9	24.4	0.227	P
	4	J21	J96	J98	J95							260	806	20	535	92.9	24.4	0.226	P
	5	J21	J96	J98	J95	J12						261	866	19	475	93.2	23.2	0.216	P
	6	J21	J96	J98	J95	J12	J38					261	867	19	474	93.2	23.1	0.216	P
	7	J21	J96	J98	J95	J12	J38	J93				261	867	19	474	93.2	23.1	0.216	P
	8	J21	J96	J98	J95	J12	J38	J93	J22			267	944	13	397	95.4	22.0	0.210	P
	9	J21	J96	J98	J95	J12	J38	J93	J22	J05		267	945	13	396	95.4	22.0	0.210	P
	10	J21	J96	J98	J95	J12	J38	J93	J22	J05	J35	267	946	13	395	95.4	22.0	0.210	P
High SARI	1	J21										120	56	112	678	51.7	68.2	0.353	P
	2	J21	J96									213	426	19	308	91.8	33.3	0.306	P
	3	J21	J96	J95								213	428	19	306	91.8	33.2	0.305	P
	4	J21	J96	J95	J38							213	429	19	305	91.8	33.2	0.305	P
	5	J21	J96	J95	J38	J98						219	468	13	266	94.4	31.9	0.301	P
	6	J21	J96	J95	J38	J98	J12					219	504	13	230	94.4	30.3	0.286	P
	7	J21	J96	J95	J38	J98	J12	J05				219	504	13	230	94.4	30.3	0.286	P
	8	J21	J96	J95	J38	J98	J12	J05	J15			220	511	12	223	94.8	30.1	0.285	P
	9	J21	J96	J95	J38	J98	J12	J05	J15	J93		220	511	12	223	94.8	30.1	0.285	P
	10	J21	J96	J95	J38	J98	J12	J05	J15	J93	J35	220	511	12	223	94.8	30.1	0.285	P
Low SARI	1	J21										19	43	29	564	39.6	30.6	0.121	P
	2	J21	J96									36	312	12	295	75.0	10.3	0.078	P
	3	J21	J96	J98								41	338	7	269	85.4	10.8	0.092	P
	4	J21	J96	J98	J12							42	362	6	245	87.5	10.4	0.091	P
	5	J21	J96	J98	J12	J20						42	375	6	232	87.5	10.1	0.088	P
	6	J21	J96	J98	J12	J20	J04					42	378	6	229	87.5	10.0	0.088	P
	7	J21	J96	J98	J12	J20	J04	J18				44	465	4	142	91.7	8.6	0.079	P

	8	J21	J96	J98	J12	J20	J04	J18	J22			46	501	2	106	95.8	8.4	0.081	P
	9	J21	J96	J98	J12	J20	J04	J18	J22	J45		47	515	1	92	97.9	8.4	0.082	P
	10	J21	J96	J98	J12	J20	J04	J18	J22	J45	J44	47	538	1	69	97.9	8.0	0.079	P
0-4	1	J21										138	92	96	305	59.0	60.0	0.354	P
	2	J21	J12									157	103	77	294	67.1	60.4	0.405	P
	3	J21	J12	J96								219	336	15	61	93.6	39.5	0.369	P
	4	J21	J12	J96	J95							219	337	15	60	93.6	39.4	0.369	P
	5	J21	J12	J96	J95	J93						219	337	15	60	93.6	39.4	0.369	P
	6	J21	J12	J96	J95	J93	U071					220	337	14	60	94.0	39.5	0.371	P
	7	J21	J12	J96	J95	J93	U071	J38				220	337	14	60	94.0	39.5	0.371	P
	8	J21	J12	J96	J95	J93	U071	J38	J98			225	348	9	49	96.2	39.3	0.378	P
	9	J21	J12	J96	J95	J93	U071	J38	J98	J05		225	349	9	48	96.2	39.2	0.377	P
	10	J21	J12	J96	J95	J93	U071	J38	J98	J05	J85	225	349	9	48	96.2	39.2	0.377	P
5-14	1	J12										2	2	2	67	50.0	50.0	0.250	P
	2	J12	J95									2	2	2	67	50.0	50.0	0.250	P
	3	J12	J95	J13								2	3	2	66	50.0	40.0	0.200	P
	4	J12	J95	J13	J84							2	4	2	65	50.0	33.3	0.167	P
	5	J12	J95	J13	J84	J96						4	53	0	16	100.0	7.0	0.070	P
	6	J12	J95	J13	J84	J96	J98					4	59	0	10	100.0	6.3	0.063	P
	7	J12	J95	J13	J84	J96	J98	J45				4	61	0	8	100.0	6.2	0.062	P
	8	J12	J95	J13	J84	J96	J98	J45	J00			4	61	0	8	100.0	6.2	0.062	P
	9	J12	J95	J13	J84	J96	J98	J45	J00	J01		4	61	0	8	100.0	6.2	0.062	P
	10	J12	J95	J13	J84	J96	J98	J45	J00	J01	J02	4	61	0	8	100.0	6.2	0.062	P
15-64	1	J98										5	12	5	192	50.0	29.4	0.147	P
	2	J98	J96									7	86	3	118	70.0	7.5	0.053	P
	3	J98	J96	J22								8	104	2	100	80.0	7.1	0.057	P
	4	J98	J96	J22	J15							8	108	2	96	80.0	6.9	0.055	P
	5	J98	J96	J22	J15	J91						8	109	2	95	80.0	6.8	0.055	P
	6	J98	J96	J22	J15	J91	J45					9	119	1	85	90.0	7.0	0.063	P
	7	J98	J96	J22	J15	J91	J45	J44				9	124	1	80	90.0	6.8	0.061	P
	8	J98	J96	J22	J15	J91	J45	J44	J10			9	131	1	73	90.0	6.4	0.058	P
	9	J98	J96	J22	J15	J91	J45	J44	J10	J12		9	146	1	58	90.0	5.8	0.052	P
	10	J98	J96	J22	J15	J91	J45	J44	J10	J12	J18	10	177	0	27	100.0	5.3	0.053	P
65+	1	J98										15	82	17	589	46.9	15.5	0.072	P
	2	J98	J96									25	313	7	358	78.1	7.4	0.058	P
	3	J98	J96	J22								29	376	3	295	90.6	7.2	0.065	P
	4	J98	J96	J22	J45							29	396	3	275	90.6	6.8	0.062	P
	5	J98	J96	J22	J45	J20						29	406	3	265	90.6	6.7	0.060	P
	6	J98	J96	J22	J45	J20	J06					30	406	2	265	93.8	6.9	0.065	P
	7	J98	J96	J22	J45	J20	J06	J21				30	406	2	265	93.8	6.9	0.065	P
	8	J98	J96	J22	J45	J20	J06	J21	J44			31	455	1	216	96.9	6.4	0.062	P
	9	J98	J96	J22	J45	J20	J06	J21	J44	J12		32	483	0	188	100.0	6.2	0.062	P
	10	J98	J96	J22	J45	J20	J06	J21	J44	J12	J42	32	490	0	181	100.0	6.1	0.061	P

SPAIN

SARI- any of the three		ICD 1	ICD 2	ICD 3	ICD 4	ICD 5	ICD 6	ICD 7	ICD 8	ICD 9	ICD 10	TP	FP	FN	TN	Se (%)	PPV(%)	CUI	
All ages	1	U071										273	21	376	914	42.1	92.9	0.391	P
	2	U071	J21									411	109	238	826	63.3	79.0	0.501	S
	3	U071	J21	J12								437	121	212	814	67.3	78.3	0.527	S
	4	U071	J21	J12	J10							512	126	137	809	78.9	80.3	0.633	S
	5	U071	J21	J12	J10	J96						601	569	48	366	92.6	51.4	0.476	P
	6	U071	J21	J12	J10	J96	J09					606	569	43	366	93.4	51.6	0.482	P
	7	U071	J21	J12	J10	J96	J09	J84				606	578	43	357	93.4	51.2	0.478	P
	8	U071	J21	J12	J10	J96	J09	J84	J22			621	635	28	300	95.7	49.4	0.473	P
	9	U071	J21	J12	J10	J96	J09	J84	J22	J95		621	636	28	299	95.7	49.4	0.473	P
	10	U071	J21	J12	J10	J96	J09	J84	J22	J95	J98	629	667	20	268	96.9	48.5	0.470	P
High SARI	1	U071										179	10	289	470	38.2	94.7	0.362	P
	2	U071	J21									298	57	170	423	63.7	83.9	0.535	S
	3	U071	J21	J12								318	64	150	416	67.9	83.2	0.566	S

	4	U071	J21	J12	J10							367	68	101	412	78.4	84.4	0.662	G
	5	U071	J21	J12	J10	J96						444	310	24	170	94.9	58.9	0.559	S
	6	U071	J21	J12	J10	J96	J09					448	310	20	170	95.7	59.1	0.566	S
	7	U071	J21	J12	J10	J96	J09	J84				448	313	20	167	95.7	58.9	0.564	S
	8	U071	J21	J12	J10	J96	J09	J84	J95			448	314	20	166	95.7	58.8	0.563	S
	9	U071	J21	J12	J10	J96	J09	J84	J95	J05		448	314	20	166	95.7	58.8	0.563	S
	10	U071	J21	J12	J10	J96	J09	J84	J95	J05	J92	448	314	20	166	95.7	58.8	0.563	S
**Low SARI**	1	U071										94	11	87	444	51.9	89.5	0.465	P
	2	U071	J12									100	16	81	439	55.2	86.2	0.476	P
	3	U071	J12	J10								126	17	55	438	69.6	88.1	0.613	S
	4	U071	J12	J10	J22							137	65	44	390	75.7	67.8	0.513	S
	5	U071	J12	J10	J22	J96						164	276	17	179	90.6	37.3	0.338	P
	6	U071	J12	J10	J22	J96	J21					167	284	14	171	92.3	37.0	0.342	P
	7	U071	J12	J10	J22	J96	J21	J84				167	289	14	166	92.3	36.6	0.338	P
	8	U071	J12	J10	J22	J96	J21	J84	J09			168	289	13	166	92.8	36.8	0.341	P
	9	U071	J12	J10	J22	J96	J21	J84	J09	J98		173	299	8	156	95.6	36.7	0.350	P
	10	U071	J12	J10	J22	J96	J21	J84	J09	J98	J45	175	312	6	143	96.7	35.9	0.347	P
**0-4**	1	J10										22	0	237	357	8.5	100.0	0.085	P
	2	J10	J21									164	81	95	276	63.3	66.9	0.424	P
	3	J10	J21	J12								183	91	76	266	70.7	66.8	0.472	P
	4	J10	J21	J12	U071							188	92	71	265	72.6	67.1	0.487	P
	5	J10	J21	J12	U071	J96						246	297	13	60	95.0	45.3	0.430	P
	6	J10	J21	J12	U071	J96	J95					246	298	13	59	95.0	45.2	0.430	P
	7	J10	J21	J12	U071	J96	J95	J93				246	298	13	59	95.0	45.2	0.430	P
	8	J10	J21	J12	U071	J96	J95	J93	J38			246	298	13	59	95.0	45.2	0.430	P
	9	J10	J21	J12	U071	J96	J95	J93	J38	J98		250	308	9	49	96.5	44.8	0.432	P
	10	J10	J21	J12	U071	J96	J95	J93	J38	J98	J20	250	312	9	45	96.5	44.5	0.429	P
**5-14**	1	J12										4	0	10	57	28.6	100.0	0.286	P
	2	J12	U071									6	0	8	57	42.9	100.0	0.429	P
	3	J12	U071	J95								6	0	8	57	42.9	100.0	0.429	P
	4	J12	U071	J95	J99							6	0	8	57	42.9	100.0	0.429	P
	5	J12	U071	J95	J99	J10						9	1	5	56	64.3	90.0	0.579	S
	6	J12	U071	J95	J99	J10	J96					13	43	1	14	92.9	23.2	0.216	P
	7	J12	U071	J95	J99	J10	J96	J20				14	43	0	14	100.0	24.6	0.246	P
	8	J12	U071	J95	J99	J10	J96	J20	J13			14	44	0	13	100.0	24.1	0.241	P
	9	J12	U071	J95	J99	J10	J96	J20	J13	J84		14	44	0	13	100.0	24.1	0.241	P
	10	J12	U071	J95	J99	J10	J96	J20	J13	J84	J98	14	50	0	7	100.0	21.9	0.219	P
**15-64**	1	U071										53	7	29	120	64.6	88.3	0.571	S
	2	U071	J10									69	8	13	119	84.1	89.6	0.754	G
	3	U071	J10	J12								70	9	12	118	85.4	88.6	0.756	G
	4	U071	J10	J12	J96							75	54	7	73	91.5	58.1	0.532	S
	5	U071	J10	J12	J96	J84						75	55	7	72	91.5	57.7	0.528	S
	6	U071	J10	J12	J96	J84	J98					76	60	6	67	92.7	55.9	0.518	S
	7	U071	J10	J12	J96	J84	J98	J22				77	73	5	54	93.9	51.3	0.482	P
	8	U071	J10	J12	J96	J84	J98	J22	J09			78	73	4	54	95.1	51.7	0.491	S
	9	U071	J10	J12	J96	J84	J98	J22	J09	J68		78	73	4	54	95.1	51.7	0.491	S
	10	U071	J10	J12	J96	J84	J98	J22	J09	J68	J45	80	80	2	47	97.6	50.0	0.488	P
**65+**	1	U071										202	13	92	381	68.7	94.0	0.646	G
	2	U071	J12									207	14	87	380	70.4	93.7	0.659	G
	3	U071	J12	J10								244	17	50	377	83.0	93.5	0.776	G
	4	U071	J12	J10	J98							258	60	36	334	87.8	81.1	0.712	G
	5	U071	J12	J10	J98	J09						262	60	32	334	89.1	81.4	0.725	G
	6	U071	J12	J10	J98	J09	J84					262	73	32	321	89.1	78.2	0.697	G
	7	U071	J12	J10	J98	J09	J84	J96				275	197	19	197	93.5	58.3	0.545	S
	8	U071	J12	J10	J98	J09	J84	J96	J92			275	197	19	197	93.5	58.3	0.545	S
	9	U071	J12	J10	J98	J09	J84	J96	J92	J69		277	203	17	191	94.2	57.7	0.544	S
	10	U071	J12	J10	J98	J09	J84	J96	J92	J69	J22	289	237	5	157	98.3	54.9	0.540	S

Abbreviations; CUI: clinical utility index. CUI E: Excellent utility. CUI G: Good utility. CUI S: Satisfactory utility. CUI P: Poor utility. FN: false negative. FP: False Positive. ICD-10: 10th edition of the International Classification of Disease codes. PPV: positive predictive value. RSV: Respiratory Syncytial Virus. SARI: severe acute respiratory infection. SARI any of the three: SARI associated with at least one virus among influenza virus, RSV and SARS-CoV-2. SARI-influenza: SARI associated with influenza virus. SARI-RSV: SARI associated with RSV. SARI-SARS-CoV-2: SARI associated with SARS-CoV-2. SARS-CoV-2: Severe Acute Respiratory Syndrome Coronavirus 2. Se: sensitivity. Strata All ages: All hospitalisation episodes, regardless of

patient age, Strata High SARI: Period of high SARI activity, Strata Low SARI: Period of low SARI activity, Strata 0-4: Hospitalisation episodes for patients aged 0 to 4 years, Strata 5-14: Hospitalisation episodes for patients aged 5 to 14 years, Strata 15-64: Hospitalisation episodes for patients aged 15 to 64 years, Strata 65+: Hospitalisation episodes for patients aged 65 years and older. TN: true negative. TP: true positive. Please refer to Supplementary Text 1 for a description of the ICD-10 codes.
